# Metabolic, Physiological, and Transcriptomics Analysis of Batch Cultures of the Green Microalga *Chlamydomonas* Grown on Different Acetate Concentrations

**DOI:** 10.3390/cells8111367

**Published:** 2019-10-31

**Authors:** Kenny A. Bogaert, Emilie Perez, Judith Rumin, Axel Giltay, Michele Carone, Nadine Coosemans, Michele Radoux, Gauthier Eppe, Raphael D. Levine, Francoise Remacle, Claire Remacle

**Affiliations:** 1 Theoretical Physical Chemistry, MolSys Research Unit, University of Liège, 4000 Liège, Belgium; bogaert.kenny@gmail.com; 2 Genetics and Physiology of Microalgae, InBios/Phytosystems Research Unit, University of Liège, 4000 Liège, Belgiumjudith.rumin@gmail.com (J.R.); axelgiltay@gmail.com (A.G.); michele.carone@uliege.be (M.C.); nadine.coosemans@uliege.be (N.C.); m.radoux@uliege.be (M.R.); 3 Mass Spectrometry Laboratory, MolSys Research Unit, University of Liège, 4000 Liège, Belgium; g.eppe@uliege.be; 4 The Fritz Haber Research Center for Molecular Dynamics, Institute of Chemistry, Hebrew University of Jerusalem, Jerusalem 91904, Israel; rafi@fh.huji.ac.il; 5 Department of Molecular and Medical Pharmacology, David Geffen School of Medicine, University of California Los Angeles, Los Angeles, CA 90095, USA

**Keywords:** *Chlamydomonas*, growth, acetate concentration, transcriptomics, surprisal analysis

## Abstract

Acetate can be efficiently metabolized by the green microalga *Chlamydomonas* *reinhardtii*. The regular concentration is 17 mM, although higher concentrations are reported to increase starch and fatty acid content. To understand the responses to higher acetate concentrations, *Chlamydomonas* cells were cultivated in batch mode in the light at 17, 31, 44, and 57 mM acetate. Metabolic analyses show that cells grown at 57 mM acetate possess increased contents of all components analyzed (starch, chlorophylls, fatty acids, and proteins), with a three-fold increased volumetric biomass yield compared to cells cultivated at 17 mM acetate at the entry of stationary phase. Physiological analyses highlight the importance of photosynthesis for the low-acetate and exponential-phase samples. The stationary phase is reached when acetate is depleted, except for the cells grown at 57 mM acetate, which still divide until ammonium exhaustion. Surprisal analysis of the transcriptomics data supports the biological significance of our experiments. This allows the establishment of a model for acetate assimilation, its transcriptional regulation and the identification of candidates for genetic engineering of this metabolic pathway. Altogether, our analyses suggest that growing at high-acetate concentrations could increase biomass productivities in low-light and CO_2_-limiting air-bubbled medium for biotechnology.

## 1. Introduction

Microalgae such as *Chlamydomonas* are able to store energy in the form of starch and oil (triacylglycerol or TAG) under certain conditions. The oil has especially drawn interest as a potential source of renewable fuels. However, the molecular and cellular mechanisms underlying the regulation of the two carbon sinks are poorly understood. Identification of pathways and regulatory networks that underly oil production could guide genetic engineering for increased utilization of the TAG carbon sink and overproduction of oil.

Oil accumulation can be stimulated under N starvation and can be increased by additional carbon supply in the form of acetate [1]. Unlike the response to nitrogen deprivation, the response to additional acetate is not as well understood. The green microalga *Chlamydomonas reinhardtii* is able to use acetate for growth in the light (mixotrophy) and in the dark (heterotrophy). After transport into the cell, acetate is metabolized into acetyl-CoA. Acetyl-CoA enters the glyoxylate cycle which allows the formation of building blocks such as amino acids and glucose or enters the tricarboxylic acid (TCA) cycle to feed the respiratory chain with reducing equivalents. Both the glyoxylate cycle and the respiratory chain are essential for growth in the dark since *Chlamydomonas* mutants affected in one of the processes cannot grow in heterotrophy [2,3].

The usual concentration of acetate for *Chlamydomonas* growth is 17 mM [4], and most of the papers utilize this concentration for cultivation. Few papers have analyzed cell physiology using other acetate concentrations. Heifetz et al. (2000) [5] showed that photosynthesis is inhibited when *Chlamydomonas* cells are cultivated in the presence of 3.7 to 29.4 mM acetate in saturating light and CO_2_, showing that acetate is the preferred carbon source. Growth rate was unaffected despite the large reduction of photosynthesis. Moreover, cultivating cells for two days with 80 mM acetate in low light and air increases the starch amount per cell more than the levels of TAG, showing that starch is the main carbon sink in *Chlamydomonas* [1]. These facts make the case for an effective acetate assimilation in the light, but the detailed information explaining how cells adapt to acetate and whether this cultivation mode could be useful for biotechnological purposes is lacking.

Batch cultures typically comprise an exponential phase when nutrients are in sufficient amounts to sustain high growth rates, followed by a stationary phase where divisions cease and cell number is maintained. Subsequently, cells may enter a death phase [6]. Yeast cells cultivated in glucose shift from a fermentative to a respiratory metabolism upon depletion of the carbon source at the entry in the stationary phase, which is followed by post-translational modifications and protection against oxidative stress [7]. In addition, stationary phase is characterized by increased levels of transcripts encoding enzymes involved in sugar metabolism, the TCA cycle, and amino acid degradation [8]. In *Chlamydomonas*, entry into the stationary phase is marked by the activation of CrATG8 [9], the main marker of autophagy. RT-qPCR analyses also showed that cultures in stationary phase present increased activity and transcripts of some of the enzymes participating in the antioxidant system. Programmed cell death was detected by an increase of caspase activity [10]. It thus seems that yeast and *Chlamydomonas* cells present some common responses, such as the activation of antioxidant systems, but more details are needed to get a comprehensive view of the process.

With the aim of characterizing the response of *Chlamydomonas* cells to acetate and to the conditions of the different growth phases, we grew the cells in the presence of four acetate concentrations, the usual (17 mM) one and three higher concentrations (31, 44, and 57 mM) in batch cultures bubbled with air and grown under low light. We sampled the cultures at different time points of the exponential and stationary phase of growth and analyzed the metabolic, physiological, and transcriptomics responses. The transcript response to different acetate concentrations was analyzed using surprisal analysis (SA), a method grounded in thermodynamics [11,12,13,14,15,16]. Surprisal analysis allows for the determination of the constraints on the genomic response due to different acetate concentrations. Two different constraints were identified that led to two different phenotypes. The first one comprises pathways involved in exponential/stationary phase, and the second comprises pathways involved in the low/high-acetate growth mode. Combined with oxygen evolution and metabolic measurements, our analysis showed that cultivation in low light and air supplemented by higher acetate concentrations than usual could be relevant to biotechnological applications aiming at increasing volumetric biomass yields, for example, during early stage culture expansion. In addition, acetic acid is a key element for synergetic production of biohydrogen by both *Chlamydomonas* and bacteria during cocultivation [17].

## 2. Materials and Methods

### 2.1. Strains and Cultivation Conditions

Strains WT and *iclC* were described previously ([2,18] and are both derived from the 137 C strain of *C. reinhardtii* [19]. They were first adapted for 48 h in Tris-Phosphate medium [4], adjusted to pH 7.0 with HCl, with different acetate concentrations (17, 31, 44, 57, and 87 mM sodium acetate) in flasks or in small photobioreactors (Multi-Cultivator MC 1000-OD, Photon Systems Instruments). Experimental cultivations were started with an initial cell concentration around 2 × 10^5^ cells per mL, at 26 ± 1 °C, under moderate light (50 µmol.m^−2^.s^−1^), and followed for 145 h. Cells were counted using a Coulter counter (http://www.beckmancoulter.com). The number of divisions per day was calculated according to [20]. Cell size (maximal diameter, estimated on 100 cells) was determined under a microscope, using the Imaging Software, NIS Elements, version 4, for Nikon (Nikon Instruments Inc., Melville, NY, USA).

### 2.2. Samples and RNA Extraction

Four time points were selected for sampling the transcripts: 12, 28, 50, and 70 h, using the four acetate concentrations. Three biological replicates were made for each curve and each time point. Twenty milliliters were sampled at 12 h, corresponding approximately to 1.5 × 10^7^ cells; 15 mL at 28 h and 5 mL at each other time point, corresponding approximately to 5.5 × 10^7^ cells. Cells were harvested by centrifugation for 10 min at 1500× *g*. Total RNA extraction was performed according to [21]. Cells were washed in 500 µL of TEN buffer (10 mM Tris-HCl pH 8.0, 10 mM EDTA, and 150 mM NaCl) and suspended in 150 µL of RNase-free water before being stored at −20 °C. After all samples were collected, total RNA was extracted with 300 µL of SDS-EB buffer (2% SDS, 400 mM NaCl, 40 mM EDTA, and 100 mM Tris-HCl pH 8.0) and 350 µL of phenol/chloroform/isoamyl alcohol (25:24:1), and the aqueous phase was collected by centrifugation (5 min at 15,000× *g*). This aqueous phase was used for the second extraction, with 300 µL of chloroform/isoamyl alcohol (24:1), followed by centrifugation (5 min at 15,000× *g*). The aqueous phase was used to precipitate RNA with 125 µL of LiCl 8 M at 4 °C overnight. Finally, the total RNA pellets were obtained by removing the supernatant after centrifugation for 5 min, at 15,000× *g*, and washed with 300 µL of 70% ethanol. After drying, the total RNA was suspended in 50 µL of RNase-free water and stored at −20 °C.

### 2.3. Sequencing

Library preparation started with 500 ng total RNA per sample. Illumina Sequencing (PE 1 × 75 on a NextSeq500 machine) was performed at the GIGA-R Sequencing platform (University of Liège), following the manufacturer’s protocol (Illumina Inc, San Diego, CA, USA).

### 2.4. Read Trimming and Quality Filtering

Read quality was assessed with FastQC v.0.11.5 (www.bioinformatics.babraham.ac.uk/projects/). No significant problems were observed. Quality filtering of RNA-seq samples was done on single-end reads, using trimmomatic (v0.36) [22], removing low-quality sequences (average Q20 over a 4-base sliding window, minimum length = 50 bp, with a leading and trailing quality threshold of Q25).

### 2.5. Read Mapping

Mapping of the reads to the *Chlamydomonas reinhardtii* genome v5.5 assembly [23] was done using STAR [24], with default presets, except for intron size (-alignIntronMin 20 and -alignIntronMax 3000). The RNA-seq data are available under the project number PRJNA561092.

The unique mapping reads were assigned to the primary transcripts, using cuffquant and cuffdiff (v2.2.1), with the default fragment size of 200 and standard deviation of 80 [25]. Expression estimates were normalized to library size and gene length by cufflinks to calculate the FPKM values.

### 2.6. Surprisal Analysis

Surprisal analysis was carried out as described in [11] on 10,923 genes. In surprisal analysis, the natural logarithm of the gene expression values *X_i_*(*s*) of gene *i* in sample *s* is given as a sum of a balanced state value, a value common to all the samples and terms representing deviations from the balanced state. The deviations represent biological constraints particular to sample *s*:

(1) $$Y_{i}\;\left(s\right)=\;\ln X_{i}\;\left(s\right)=\;\ln X_{i}^{0}+\overset{N_{\alpha }}{\underset{\alpha =1}{\sum }}\;G_{i\alpha }\;\lambda_{\alpha }\;\left(s\right)$$

Here, α is the index of the deviation terms, the constraints, and $N_{a}$ is the number of constraints. Each constraint, *α*, corresponds to a given phenotype, defined by the weights $G_{i\alpha }$ of the different genes i. The phenotypes, $G_{i\alpha }$, and Lagrange multipliers, $\lambda_{\alpha }\left(s\right)$, are determined via singular value decomposition (SVD) [12]. For more details, see Appendix A. The first term in Equation (1) is the contribution of the balanced state, which is common to all the samples. The balanced state can also be written as $\ln X_{i}^{0}=\;G_{i0}\lambda_{0}$. Changes in the gene expression levels due to the successive constraints, α = 1, … $N_{\alpha }$, are expressed with respect to the balanced state. By plotting the values of the Lagrange multipliers of the different samples for a given constraint (*α*), one can identify different groups of samples that differ by the sign of their Lagrange multiplier, $\lambda_{\alpha }\left(s\right)$, within a given phenotype. In particular, we show below that, for the first constraint, *α* = 1, samples at stationary phase and those at exponential phase have an opposite sign of their Lagrange multipliers. For *α* =2, samples grown in low-acetate concentrations and samples grown in high-acetate concentrations are characterized by Lagrange multipliers of different signs. The analysis of the corresponding phenotype allows for the identification of the pathways that contribute most to the growth in low- and high-acetate concentrations, respectively. Error bars on the $\lambda_{\alpha }\left(s\right)$’s can be determined from the experimental error (see Appendix A).

### 2.7. Differential Gene Expression in the Constraint Vector G_i__α_

Genes of the phenotype corresponding to each constraint (α) were ranked according to the value of the weight (*G_i__α_*). According to this ranking, 100 smallest and largest values were considered differentially expressed for each phenotype. In the case of the balanced state, genes that correspond to a term, $G_{i0}\lambda_{0}>0$, are the most stable, and those for which $G_{i0}\lambda_{0}<0$ are unstable. The latter are the genes that will appear with the largest and the smaller weights in the phenotypes associated with the constraints and therefore will be the most differentially expressed in the constraints, α = 1, …, $N_{\alpha }$.

### 2.8. Gene Set Enrichment

Genes were categorized in gene sets, using the Kyoto Encyclopedia of Genes and Genomes (KEGG) (http://www.genome.jp/kegg/), and the functional annotation for *C. reinhardtii* v5.5 predicted proteins obtained from the correspondence table downloaded from Phytozome. Transcription factors, transcriptional regulators, and protein kinases were retrieved from the iTAK database (http://itak.feilab.net/). KEGG Orthology identifiers were mapped to KEGG pathways, using KEGGREST package [26]⁠. To assign a weight to pathways in a given phenotype α, we use the approach proposed in [11], which takes into account the weights of all the genes, *G_i__α_*, of the phenotype α. The weight assigned to a pathway therefore does not depend on the number of genes kept in the differential gene expression analysis.

The weights of the pathways are assigned as follows. For each constraint, α, each subset of *N_J_* genes corresponding to a pathway, *J*, was divided in two subsets, according to the sign of their weight (*G_i__α_*). The *G_i__α_* values for genes that are respectively larger or smaller than zero were summed together to get respectively the positive (P) and negative (N) weight of the pathway for constraint (α):

(2) $$P_{\alpha }^{J}=\;\overset{N_{j}}{\underset{i=1}{\sum }}G_{i\alpha }^{2}\;if\;G_{i\alpha }\;>0$$

(3) $$N_{\alpha }^{J}=\;\overset{N_{j}}{\underset{i=1}{\sum }}G_{i\alpha }^{2}\;if\;G_{i\alpha }\;<0$$

The set ratio of the pathway (*J*) is as follows:

(4) $$SR_{J}^{\alpha }=\;P_{\alpha }^{J}/\;N_{\alpha }^{J}$$

It is a measure for the contribution of the gene set of the pathway (*J*) to constraint (α). Set ratios, $SR_{J}^{a}$, were ordered according to their value for each phenotype. These gene sets where all values, *G_i__α_*, are either positive or negative, were subsequently ranked on $P_{\alpha }^{J}$ or $N_{\alpha }^{J}$, respectively. Pathways that correspond to a gene set with less than 10 genes were omitted from the dataset.

Pathways with both low and high ratios are predicted by surprisal analysis to be important for the phenotype and to be enriched. In the balanced state, genes that correspond to a term $G_{i0}\lambda_{0}\;>0$ are the most stable, and those for which $G_{i0}\;\lambda_{0}<0$ are unstable. In the first phenotype, α=1, genes which correspond to a term $G_{i1}\lambda_{1}\left(s\right)\;>0$ are over expressed for samples grown in exponential phase and under expressed for samples in stationary phase while for the second phenotype, α=2, genes for which $G_{i2}\lambda_{2}\left(s\right)\;>0$ are over expressed in high-acetate conditions and under expressed in low-acetate conditions. Since the values of the Lagrange multiplier, $\lambda_{1}\left(s\right)$, are positive for the samples in exponential phase and negative for those in stationary phase, high SR pathway ratios correspond to gene sets that are over expressed for samples in exponential phase and low SR ratios correspond to gene sets that are over expressed in stationary phase. For the second constraint, samples grown in high-acetate concentrations have a positive Lagrange multiplier $\lambda_{2}\left(s\right)$, while samples grown in low-acetate concentrations have negative $\lambda_{2}\left(s\right)$ values. Therefore, high SR pathway ratios correspond to gene sets that are over expressed for samples grown in high-acetate conditions, while low SR ratios values correspond to gene sets that are over expressed in low-acetate concentrations.

### 2.9. Metabolic Analyses

NH_4_^+^ and acetate concentrations were determined using kits from Megazyme (L-Arginine/urea/ammonium for NH_4_^+^ and Acetic Acid Assay kit for acetate). Fatty acids were quantified by GC–MS, as already described in [18]. Starch content and dry weight were measured as also described in [18].

### 2.10. Oxygen Evolution Measurements

Cells were collected at each time point, and measurements were made directly on 1 mL of the cell suspensions. Oxygen evolution rates (gross oxygen evolution) were measured using Clark Electrode (Hansatech Oxygraph). The assayed actinic light steps included 6, 14, 30, 45, 68, 103, 176, 301, 571, 1024, and 1931 μmol.m^−2^ s^−1^. Oxygen evolution rates were determined based on the sum of the net oxygen evolution rates over the last 45 seconds of each light step and the absolute value of the dark respiration rates.

## 3. Results

### 3.1. Growth Curves and Medium Composition of C. Reinhardtii Using Different Acetate Concentrations

**Figure 1:**
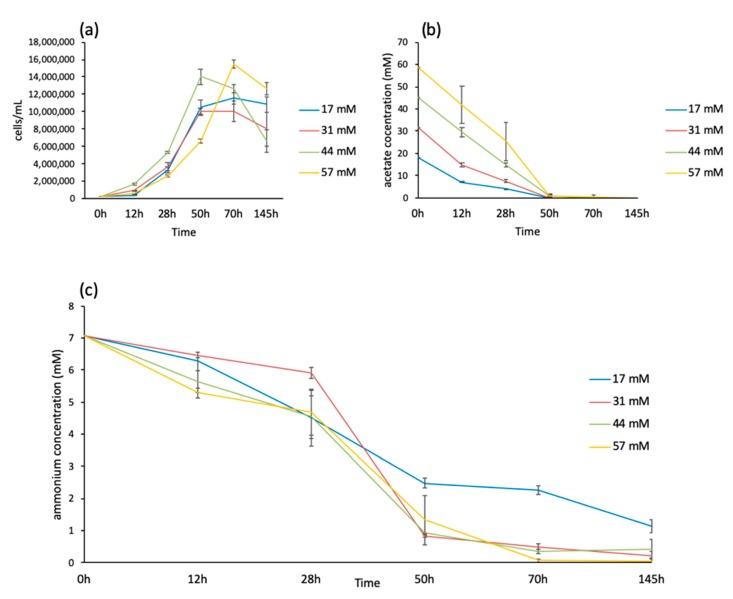
Growth curves of *Chlamydomonas (iclC* strain) and medium composition analysis at four acetate concentrations (17, 31, 44, and 57 mM) under continuous light (50 µmol·m^−2^·s^−1^) at 26 ± 1 °C. (**a**) Cell concentration (cells/mL), (**b**) acetate concentration (mM), and (**c**) ammonium concentration (NH_4_^+^) (mM). Mean of 3-4 biological replicates ± SD.

The starting growth experiments were conducted on WT (137C), and a complemented *icl* mutant strain of 137C (*iclC*), containing a wild-type version of the isocitrate lyase gene, one of the two unique enzymes of the glyoxylate cycle, because of availability of transcriptomic data for the *iclC* strain [11]. Both strains are equivalent because they both contain the *ICL1* gene and are indistinguishable at the level of their expression and physiology [11,18] (see Table S1). The cells were grown at five concentrations, 17, 31, 44, 57, and 87 mM, in triplicate, using small photobioreactors where light, temperature, and air bubbling were tightly controlled to obtain the most reproducible growth conditions. The 87 mM acetate concentration did not give reproducible growth curves and was abandoned. We thus focused on the four lowest concentrations, 17, 31, 44, and 57 mM, using the *iclC* strain as a reference. Cells were adapted to each condition for two days before starting the experiments. Cells were counted at five time points: 12, 28, 50, 70, and 145 h. The growth curves presented a pattern typical of batch cultures with an exponential and a stationary phase. Cell death can be observed between 70 and 145 h of growth at concentrations higher than 17 mM acetate (Figure 1a). A notable difference between the growth curves is that cells cultivated using 17, 31, and 44 mM as the initial acetate concentrations reached the stationary phase at time point 50 h while cells cultivated using 57 mM as initial acetate concentration reached the stationary phase at time point 70 h (Figure 1a).

In addition, the number of doublings per day is significantly higher for the cells cultivated at 17 mM compared to the three other acetate concentrations in exponential phase (Table 1).

Since stationary phase will be reached when nutrients are depleted, we measured acetate and ammonium concentrations in the medium in the four different cultures and at the five time points mentioned above (Figure 1b,c). As shown in Figure 1b, acetate is consumed at time point 50 h for all the cultures, whatever the cell concentration reached at this time, meaning that greater uptake of acetate has occurred in the cells cultivated at 57 mM acetate (Figure S1). The ammonium is also nearly entirely consumed at time point 70 h, except for the cells cultivated using 17 mM acetate, where around one-third of the initial ammonium concentration still remains in the medium (Figure 1c). These results suggest that the stationary phase begins when the carbon source is depleted, except for cells cultivated at 57 mM acetate, which reach the stationary phase when the ammonium source is consumed and thus still divide exponentially until 70 h of growth.

### 3.2. Biomass Composition Analysis

**Figure 2:**
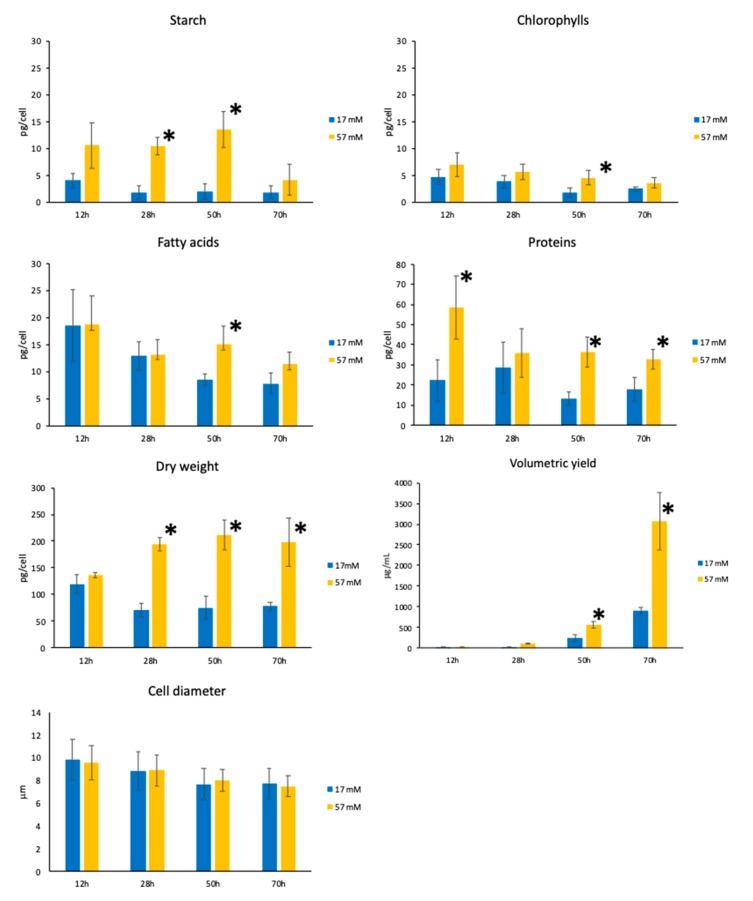
Biomass composition, dry weight, volumetric yield, and cell size (cell diameter) *(iclC* strain) during growth, using the two extreme concentrations (17 and 57 mM) of acetate for cultivation. Mean of at least three independent experiments ± SD. * Significant difference (*p* < 0.05) between 17 and 57 mM acetate time points.

Biomass composition, cell dry weight, biomass volumetric yield, and cell size of cells cultivated at the extreme acetate concentrations, 17 and 57 mM acetate, were analyzed at the first four time points (12, 28, 50, and 70 h) (Figure 2). The time point 145 h had to be discarded because of a variability probably that was too high due to variable cell death. Transesterification and GC analysis were used to determine fatty acid methyl ester (FAME) content and profile, while the contents of other components (chlorophyll, proteins, and starch) were determined spectrophotometrically (see Material and Methods). A general increase of all biomass components per cell is observed when cells are cultivated at 57 mM acetate, with significant differences present at least for one time point of the growth curves. Cell dry weight is also significantly higher, leading to a three-fold increase of biomass volumetric yield at the entry of the stationary phase for cells cultivated at 57 mM acetate. Cell size estimated by the longest diameter of the cells is not significantly different. The profiles of fatty acids were also examined and were found to be similar in both types of cultivation conditions: saturated fatty acids are major classes of the total fatty acids, whatever the concentration and the time point of the growth curve (Table S2 and Figure S2).

### 3.3. O_2_ Evolution

**Figure 3:**
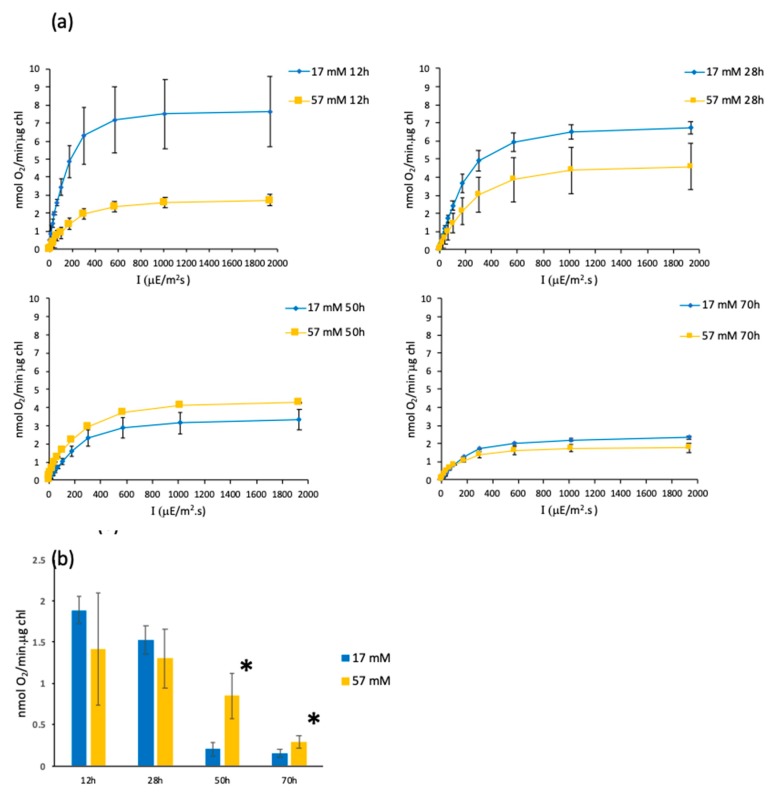
Oxygen evolution curves and respiration rates during growth using the two extreme acetate concentrations (17 and 57 mM) for the *iclC* strain. (**a**) Oxygen evolution at different light intensities for, respectively, 12, 28, 50, and 70 h after inoculation. (**b**) Respiration rates. Mean of at least three independent experiments ± SD. * Significant difference (*p* < 0.05).

Since photosynthesis and respiration are the two major processes yielding ATP production, we measured the oxygen evolution during growth (Figure 3) by determination of photosynthesis–irradiance curves, using low-acetate (17 mM) and high-acetate (57 mM) growth curves (Figure 3a).

The highest photosynthetic activities (Figure 3a) are found for the cells cultivated in low acetate (17 mM) and in exponential phase (12 and 28 h), attesting that these cells rely on energy produced by photosynthesis to divide. Cells in stationary phase show decreased photosynthetic activity compared to cells in exponential phase. Cells cultivated at 57 mM acetate demonstrate a lower (12–28 h) or about equal (50–70 h) oxygen evolution compared to cells at 17 mM acetate. Our results are in agreement with those of [5,27], which showed that the presence of acetate decreases photosynthetic activity. Respiration was also recorded (Figure 3b). The cells cultivated at 57 mM acetate present a similar (12–28 h) or significantly higher (50–70 h) respiration rate than cells cultivated at 17 mM acetate, which show a negligible respiration rate starting from time point 50 h, when acetate was totally consumed. Presumably, this is due to the utilization of the internal carbon reserve for biosynthesis and respiration which is present in cells cultivated at high-acetate concentrations and lacking in cells cultivated in 17 mM acetate.

### 3.4. Surprisal Analysis of Transcriptomics Data

#### 3.4.1. Constraints 1 and 2 Respectively Allow the Grouping of Samples Based on Their Physiological State or the Acetate Concentration

In order to understand in detail the changes in gene expression underlying these physiological adaptations, we performed transcriptomic analyses. For that purpose, RNA was extracted in three biological replicates at the four time points (12, 28, 50, and 70 h) of the growth curves cultivated at the four acetate concentrations (17, 31, 44, and 57 mM, Figure 1), and RNA-seq data were obtained. After quality checks of the reads, one replicate of time point 70 h (31 and 44 mM) had to be discarded due to too low a fraction of uniquely mapping reads and their position as outliers on a PCA, reflecting low quality libraries (Table S3 and Figure S3). The raw RNA-seq data of time points 12 and 18 h were published previously [11] (https://www.ncbi.nlm.nih.gov/Traces/study/?acc=SRP132684) and are included in our analysis. The new data generated are available under the project number PRJNA561092. In the analysis below, the samples are labeled by a concentration and a time index, accordingly, i.e., ac1t1 means an acetate concentration of 17 mM at time t1 = 12 h, …, ac4t4 means a concentration of 57 mM and a time t4 = 70 h.

**Figure 4:**
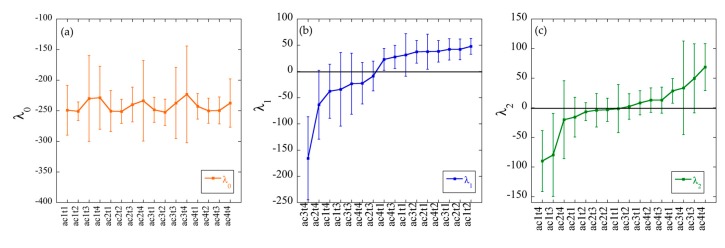
Lagrange multipliers values for (**a**) the balanced state $\left(\lambda_{0}\left(s\right)\right)$, (**b**) the first ($\left(\lambda_{1}\left(s\right)\right)$), and (**c**) the second $\left(\lambda_{2}\left(s\right)\right)$ constraint (iclC strain). The $\lambda_{0}$, $\lambda_{1}$, and $\lambda_{2}$ values and the corresponding error bars are determined using the 16 samples (see Appendix A). Note that the error bars are upper bound values (Equation (A7)). The actual error will be ≤ to the error bars shown. Overall, the sign of $\lambda_{1}$ and $\lambda_{2}$ can be considered to be reliably determined.

The values of the Lagrange multipliers and of the constraints were computed as described in [11,28] and in the Appendix. The *λ*_0_ values for each sample are plotted in Figure 4a. As expected, for the balanced state [11,28], the values of *λ*_0_*(s)* (where s stands for sample index) are constant within a range (25 units) smaller than the error bars that reflects small variations from sample to sample.

On the other hand, the values of the Lagrange multiplier of the first constraint, *λ*_1_*(s)* (Figure 4b), have different signs depending on whether the samples come from the two last time points (t3 and t4) (negative *λ*_1_: ac3t4, ac2t4, ac1t4, ac1t3, ac3t3, ac4t4, and ac2t3) or from the two first time points (t1 and t2) (positive *λ*_1_: ac4t1, ac4t3, ac1t1, ac3t1, ac2t2, ac4t2, ac3t2, ac2t1, and ac1t2), except for ac4t3 (see below). The first constraint therefore allows discrimination between the two stages of the growth curve: exponential (time points t1 and t2) or stationary (time points t3 and t4). Ac4t3 (57 mM acetate-50 h), although it is categorized as a ‘late’ sample, is found to belong to the phenotype of the exponential phase which is in accordance with the growth curve of Figure 1, where the time point 50 h of the 57 mM acetate growth curve is still in the exponential phase.

The second constraint (Figure 4c) allows the separation between the acetate concentrations of the samples, since the eight samples with low-acetate concentrations (ac1 and ac2) (ac1t4, ac1t3, ac2t4, ac2t2, ac1t2, ac2t3, ac2t1, and ac1t1) have negative values of *λ*_2_*(s)*, and the eight samples grown on high-acetate concentrations (ac3 and ac4) (ac3t1, ac3t2, ac4t2, ac4t3, ac4t1, ac3t4, ac3t3, and ac4t4) have positive values. The stationary/exponential and low/high-acetate phenotypes segregate from each other with different signs only in the plots of the Lagrange multipliers of the first and the second constraint, respectively, indicating that the gene expression profiles (phenotypes) describing these two conditions are circumscribed by the contribution of the first and the second constraint to the gene-expression levels (Table S4).

#### 3.4.2. Gene Set Enrichment Analysis Allows for the Description of the Biological Pathways Contributing to the Balanced State and to the First and Second Constraints

Surprisal analysis (see Methods) determines a gene transcript expression profile (a phenotype) associated with each constraint. This transcript expression profile is given by a vector (*G_α_*), where α is the index of the constraint. The components (*G_iα_*) of the vector (*G_α_*) determine the weight of transcript *i* in the phenotype associated with the constraint (α), whose Lagrange multiplier is λα. One can therefore rank the contribution of a transcript to a given phenotype according to its weight. As described in section ‘Gene Set Enrichment’ of Methods, the annotated genes [23] of *Chlamydomonas* were categorized in gene sets (KEGG). This categorization therefore allowed the identification of the gene sets that contribute most to the phenotype associated with a given constraint, α. From the Giα values computed for each transcript using surprisal analysis, we define weights of the pathways in each constraint and in the balanced state, Equations (2)–(4) [11,28], that quantifies the contribution to the phenotype of each gene set that defines a pathway. The pathways with the most negative weight (*N*_0_) dominate the phenotype of the balanced state (see 2.9 above), and those with the 10 most negative weights are listed in Table 2. These pathways mainly belong to primary carbon metabolism (oxidative phosphorylation, carbon fixation in photosynthetic organisms, citrate cycle, 2-oxocarboxylic acid metabolism, and pyruvate metabolism), as expected, since cells in any of the conditions analyzed need these basic pathways to survive and divide. The complete list of the KEGG pathways ranked according to the value of *N*_0_ is provided (Table S5). 22 pathways with a high negative value of *N*_0_ are added to the first ten of Table 2, which provides the fine-tuning of the balanced state.

The same analysis was carried out using the weight of each transcript, G_i1_, associated with the stationary/exponential growth phenotype of the first constraint. For this constraint, gene pathways with the highest positive weight, SR > 2 (see Equations (2)–(4)) should be considered for the characterization of the samples in exponential phase (Table S6); the 10 highest SR ratios are shown in Table 3, left. As expected from the O_2_ evolution curves, pathways in the exponential phase samples are mainly related to photosynthesis (photosynthesis, photosynthesis antenna proteins, porphyrin and chlorophyll metabolism, and carotenoid metabolism). Gene pathways with SR < 0.5 correspond to gene sets contributing most to the expression level of samples in stationary phase and the 10 lowest SR ratios are shown in Table 3, right. Pathways related to stress responses (regulation of autophagy, peroxisome, endocytosis, and selenocompound metabolism) and catabolism (valine, leucine, and isoleucine degradation) dominate in the stationary phase, which is in accordance with the physiological state of the cells at these time points of the growth curve, where some of them begin to die.

The second constraint allows for the identification of the gene pathways corresponding to acetate concentration, based on the *G_i_*_2_ values computed for each transcript. SR > 2 are those that are important for the high-acetate phenotype, and SR < 0.5 are those that are important for the low-acetate phenotype (Table S7).

The left side of Table 4 lists the ten pathways with the highest SR ratios prevailing in high-acetate concentrations. They are related to division (DNA replication), DNA damage repair, such as homologous recombination and nucleotide excision repair, and stress-related endogenous processes (proteasome and ubiquitin mediated proteolysis). The gene sets prevailing in the low-acetate phenotype, SR < 0.5, comprise pathways related to primary carbon metabolism (carbon fixation in photosynthetic organisms and pyruvate metabolism) and photosynthesis (photosynthesis, photosynthesis-antenna proteins, and carotenoid biosynthesis). They are listed in Table 4, to the right, and in Table S5.

In addition to categorizing KEGG gene sets according to their G_i0_, G_i1_, and G_i2_ values to define the biological pathways which are the most important for a specific phenotype, it is also possible to look individually at the genes contributing most to each phenotype. The first hundred genes contributing most to the balanced state (Table S8), the first phenotype (exponential phenotype or stationary) (Tables S9 and S10), and the second phenotype (high- or low-acetate phenotype) (Tables S11 and S12) are listed. Most of these have unknown functions. Those with identified functions are underlined. For the first phenotype, they mainly encode proteins participating in photosynthesis and to cell division for the exponential phase, and to transporters, stress, catabolism, and amino acid degradation for the stationary phase. For the second phenotype, they mainly encode proteins involved in ammonium starvation, but also gamete- and zygote-specific proteins, and histones for the high-acetate-phenotype. For the low-acetate phenotype, they are related to photosynthesis, low CO_2_ availability, and division. The presence of these transcripts in the corresponding phenotypes is expected. Those with unknown functions or with regulatory/specific functions are worth further investigation. For example, in the list of the first 100 genes contributing the most to the exponential phase (Table S9), Cre06.g257601, encoding a chloroplast 2-cys peroxiredoxin (PRX1) is found, which is probably involved in the redox regulation of chloroplast proteins. This enzyme deserves attention to define its targets in the regulation of the exponential phase. In the high-acetate phenotype (Table S11), Cre11.g479950, encoding a triose phosphate transporter (TPT17) is found. Five triose phosphate transporters are present in the *Chlamydomonas* genome (Cre06.g263850, TPT2; Cre08.g379350, TPT1; Cre09.g415900, TPT15; Cre12.g490100, TPT19; Cre12.g501000, TPT20; Cre16.g663800, TPT25). The fact that TPT17 is identified here and not the others suggests a role in the export of triose phosphate out of the chloroplast in the specific conditions of the high-acetate phenotype, which deserves further investigation.

In order to identify the regulatory proteins that contribute the most to the balanced state (G0), the exponential vs. stationary growth (G1), and low vs. high acetate (G2), the 100 regulatory genes contributing most to each phenotype according to their G_i_ values are summarized in Tables S13–S17. Of particular interest is the fact that, in the exponential (Table S15) and low-acetate (Table S16) phenotype, NAB1 (Cre06.g268600), which is ranked the first in these lists, was demonstrated to play a role in the adaptation to low light [29,30], while one transcription factor (Cre06.g278159/CON1) found in the low-acetate phenotype (Table S16) was recently characterized with respect to its role in the control of photoprotection during photosynthesis [31,32]. Cre09.g399552 (CCM1), which regulates CO_2_-responsive genes [33], is also ranked in the low-acetate phenotype (Table S16). These results suggest that these lists have biological significance and offer a better understanding of the regulation of central carbon metabolism. These lists thus provide candidates for genetic engineering, with the aim of upregulating or downregulating the acetate response, for example, by looking at the regulator genes in the high-acetate phenotype (Table S17), such as Cre04.g220700 encoding an Aurora like kinase (ALK2), which is ranked first in this table.

We also performed K-means clustering of expression values by using the same approach as described in [11]. Results (Figure S4) show that cluster 1 discriminates the stationary-phase samples, cluster 3 the exponential-phase samples, and cluster 2 the high-acetate samples.

### 3.5. Analysis of the Transcripts Encoding Components of the Carbon-Concentrating Mechanism (CCM)

**Figure 5:**
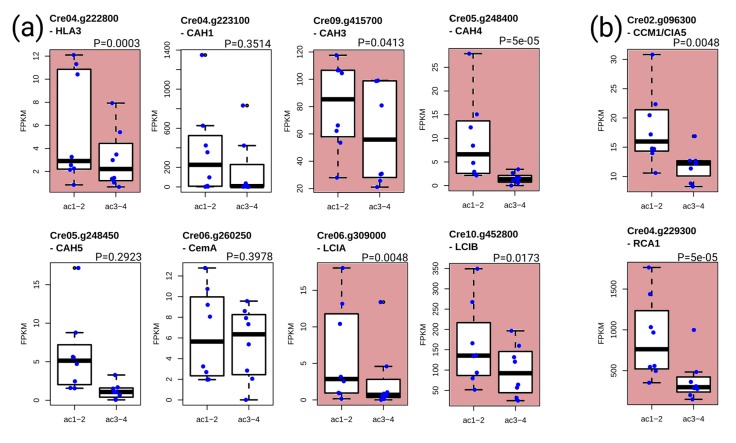
FPKM data of transcripts encoding components involved in low CO2 environment. Shown in the figure are the following. (**a**) Eight transcripts encoding components involved in hypothetical CCM/Ci transport: Cre04.g223100 (CAH1); Cre04.g222800 (HLA3); Cre05.g248400 (CAH4); Cre05.g248450 (CAH5); Cre06.g260250 (CemA); Cre06.g309000(LCIA); Cre10.g452800 (LCIB); and Cre09.g415700 (CAH3)]. (**b**) The CCM1/CIA5 (Cre02.g096300) transcript encoding the master regulator controlling the induction of the CCM mechanism, and the RCA1 (Cre04.g229300) transcript encoding rubisco activase. Ac1-2 represents the 17 and 31 mM acetate concentrations, and ac3-4 represents the 44 and 57 mM acetate concentrations. Red color denotes significant downregulation (cuffdiff, *p*-value).

Since respiration rates are higher for the cells cultivated in high acetate (Figure 3b), internal CO_2_ produced by the TCA cycle could also be increased, which could influence the expression of genes participating to the carbon-concentrating mechanism (CCM). The presence of CCM mechanisms in photosynthetic organisms is linked to the properties of the ribulose 1,5-bisphosphate carboxylase/oxygenase (Rubisco) enzyme, which cannot discriminate completely between CO_2_ and O_2_, making the oxygenation of ribulose bisphosphate competitive with the carboxylation reaction under atmospheric CO_2_. Of the eight transcripts encoding putative components of the CCM/Ci transport [34] found in our transcriptomics analysis, four show a significant decreased expression in the high-acetate cultivation conditions (ac3-4) compared to low-acetate cultivation (ac1-2) (Figure 5a). In addition, the transcript levels of CCM1/CIA5, the master regulator controlling the induction of the CCM mechanism [33] and of RCA1 encoding rubisco activase required for expression of Rubisco in low CO_2_ atmosphere [35] are also in significant lower amounts in the high-acetate phenotype (Figure 5b).

## 4. Discussion

We have analyzed the growth of *Chlamydomonas* cells cultivated at different acetate concentrations, the usual one (17 mM) and three levels above it (31, 44, and 57 mM), at four time points of the growth curves (12, 28, 50, and 70 h). Surprisal analysis of the transcriptomics data confirms the biological significance of our results. This confirms the predictive value of RNA-seq data to identify potential target genes/pathways for genetic engineering. Cells in low acetate rely on both acetate assimilation and photosynthesis for growth, which is stopped when acetate is consumed. Cells cultivated at 57 mM acetate accumulate higher amounts of starch and other biomass components (chlorophylls, fatty acids, and proteins), higher respiration rates, and lower photosynthetic activities. These observations are in line with those of [5,27], who reported higher respiration rates and decreased photosynthesis activity in acetate-grown (17 mM) cells under atmospheric CO_2_ compared to phototrophic cells. Terauchi et al. (2010) [27] also found higher chlorophyll content on a per cell basis in the acetate-grown cells compared to phototrophic-grown cells, although photosynthesis activity was decreased. Fan et al. (2012) [1] found a higher starch content when cells were cultivated with 80 mM acetate. These results and ours suggest that the excess of acetyl-CoA provided by acetate fuels carbon compound synthesis, accumulation, and degradation. This leads to an increase in biomass volumetric yields, which could be useful to boost growth for biotechnological purposes when cells are cultivated in low light and atmospheric CO_2_. Cells cultivated in high acetate seem to switch toward a heterotrophic growth mode, first by using external acetate and then by degrading their internal carbon storage compounds. Although these cells seem to suffer from stress (see gene pathways retrieved by surprisal analysis), probably because of ammonium exhaustion, they nevertheless divide using the ATP provided by the catabolic activities, and, as a consequence, cells in high acetate suffer less from the scarcity of CO_2_ due to internal recycling of CO_2_ supplied by the TCA cycle. CCM is not induced, which would also contribute to spare ATP for other biochemical pathways, such as cell division. These results corroborate those of [5,36], showing that inorganic CO_2_ transport is suppressed when chloroplasts or cells are cultivated in mixotrophic conditions.

Cells in low-acetate and exponential phase are characterized by gene and gene pathways associated with photosynthesis. Similar pathways are found in the stationary phase of *Chlamydomonas* and yeast cells, since both organisms are characterized by transcripts associated with stress-related pathways [7,8,37]. Cells enter the stationary phase when the carbon source is depleted, except for the highest acetate concentration, where the stationary phase is probably reached once ammonium is totally consumed. We also searched for transcripts involved in regulation of the stationary phase that would be common to both *Chlamydomonas* and yeast. We found that Cre08.g377550, which is ranked in the list of the first 100 genes most relevant to explain the stationary phase (Table S10), encodes a predicted Yippee-type zinc-binding protein that shares 40% identity and 61% conserved residues with yeast Moh1p, described as an essential yeast stationary-phase gene [7]. The expression of this gene is increased between 5 and 15 times at time points 50 and 70 h of the 17, 31, and 44 mM acetate growth curves compared to time points 12 and 28 h. At the last acetate concentration, only the last point shows an increase of this transcript, since time point 50 h is still in exponential phase (Figure S5). This protein is conserved among eukaryotes [38], and the human form has growth-inhibitory effects, inducing cell senescence in human cells [39]. To our knowledge, few data were reported concerning *Chlamydomonas* senescence: CrAPG8, one of the main markers for autophagy in *Chlamydomonas* was shown to be involved in the entry into the stationary phase [9], and, in fact, we find the gene pathway autophagy ranked first to explain this growth phase. Senescence was also shown to be characterized by variations in the transcript levels of some enzymes involved in the antioxidant response: Esperanza et al. (2017) [10] detected an increased level of transcripts encoding glutathione S-transferase (GST8, Cre12.g508850) and ascorbate peroxidase (APX1, Cre02.g087700) and a decreased level of those encoding catalase (CAT1, Cre09.g417150), glutathione peroxidase (GPX1, Cre02.g078300), and Mn-superoxide dismutase (SOD1, Cre02.g096150 MSD1). We found no significant variation in our database, except for APX1, whose transcript levels are decreased at the stationary phase (Figure S5). Variations in the gene regulation of antioxidant enzymes between the two experiments could be explained by different cultivation and sampling conditions, since cells in [10] were grown in minimal medium and analyzed after 96 h of growth.

## 5. Conclusion

**Figure 6:**
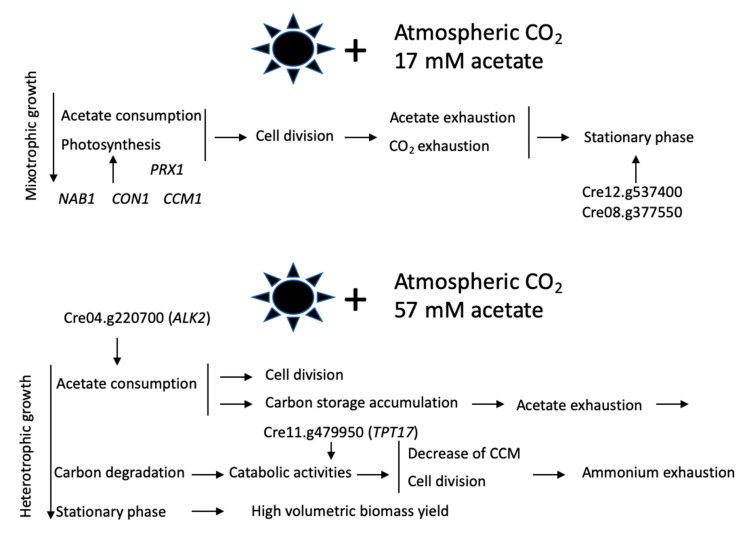
Growth mode of *Chlamydomonas* under low- and high-acetate concentrations. On the figure are shown transcripts encoding transcription factors with demonstrated function in photosynthesis (*NAB1*, *CON1*) and low CO_2_-regulating function *(CCM1*), transcripts encoding transcription factors ranked first in the list to explain the high-acetate (*ALK2*) and stationary (Cre12.g537400) phenotypes, and Cre08.g377550 encoding a Yippee-type zinc-binding protein implicated in the stationary phase in yeast. *PRX1* encodes a Cys2 peroxiredoxin, and *TPT17* encodes a triose phosphate transporter.

In conclusion, the response of *Chlamydomonas* cells to an increase of the acetate concentration in the light includes massive entrance of acetate into the cells, and its metabolization into starch and fatty acid compounds, responsible for an increase of biomass yield. We also propose that, together with the stimulation of respiration, internal CO_2_ increases, which in turn decreases the level of transcripts encoding components related to the CCM. Our findings concerning the comparison of growth mode in low and high acetate and the putative transcription factors/genes relevant for these phenotypes are summarized in Figure 6.

## Figures and Tables

**Table 1:** Number of doublings (*iclC* strain) per 24 h calculated between 12 and 50 h for the cells cultivated at 17, 31, and 44 mM acetate and between 12 and 70 h for the cells cultivated at 57 mM acetate. Mean of four biological replicates ± SD. * Significantly different (*p* < 0.05).

Acetate Concentration	Doubling/Day
17 mM	3.0 ± 0.3 *
31 mM	2.2 ± 0.3
44 mM	1.9 ± 0.1
57 mM	2.0 ± 0.1

**Table 2:** KEGG pathways contributing most to the balanced state.

KEGG Pathways	*P*_0_^1^	10^4^ × *N*_0_^2^
Oxidative phosphorylation	0	3.55
Carbon fixation in photosynthetic organisms	0	3.33
Phagosome	0	3.16
Citrate cycle (TCA cycle)	0	3.05
2-Oxocarboxylic acid metabolism	0	2.55
Valine, leucine and isoleucine biosynthesis	0	2.42
alpha-Linolenic acid metabolism	0	2.31
Lysine biosynthesis	0	2.04
Pyruvate metabolism	0	1.96
Fatty acid biosynthesis	0	1.84

^1^*P*_0_: Positive weight of the gene set in the balanced state (Equation (2) with α = 0). ^2^ *N*_0:_ Negative weight of the gene set in the balanced state (Equation (3) with α = 0).

**Table 3:** KEGG pathways contributing most to the first constraint.

Ten Most Positive Pathways (Exponential Phase)				Ten Most Negative Pathways (Stationary Phase)			
KEGG Pathways	$10^{5}\;\times \;P_{\alpha }$ ^1^	$10^{9}\;\times \;N_{\alpha }$ ^2^	$SR_{\alpha }$ ^3^	KEGG Pathways	$10^{6}\;\times \;P_{\alpha }$ ^1^	$10^{5}\;\times \;N_{\alpha }$ ^2^	$10^{2}\;\times \;SR_{\alpha }$ ^3^
Porphyrin and chlorophyll metabolism	23.50	9800	24	Regulation of autophagy	0	7.76	0
DNA replication	9.62	3930	24.5	Valine, leucine and isoleucine degradation	5.54	5.57	9.95
Carotenoid biosynthesis	4.82	1790	26.9	Endocytosis	1.88	1.37	13.7
N-Glycan biosynthesis	10.4	3700	28.2	Plant hormone signal transduction	1.92	1.36	14.1
RNA polymerase	5.85	1780	32.9	Peroxisome	8.78	3.63	24.2
Ubiquinone and other terpenoid-quinone biosynthesis	18.4	5050	36.4	Propanoate metabolism	14.5	4.61	31.4
Lysine biosynthesis	14.4	3690	38.9	beta-Alanine metabolism	20.9	5.70	36.7
Histidine metabolism	14.6	3330	43.9	SNARE interactions in vesicular transport	6.5	1.47	44.3
Photosynthesis—antenna proteins	94.2	222	4240	Selenocompound metabolism	20.4	3.74	54.5
Photosynthesis	48.4	9.37	51700	Pentose and glucuronate interconversions	7.65	1.24	61.6

^1^*P_α_*: positive weight of the gene set for constraint *α* (Equation (2)), ^2^ *N_α_*: negative weight of the gene set for constraint *α* (Equation (3)), ^3^ SR (Equation (4)): set ratios reflecting the contribution of the gene set to the phenotype. See Methods for more details about the methodology.

**Table 4:** KEGG pathways contributing most to the second constraint.

Ten Most Positive Pathways—High-Acetate-Grown Samples				Ten Most Negative Pathways—Low-Acetate-Grown Samples			
KEGG pathways	$10^{5}\;\times \;P_{\alpha }$ ^1^	10^7^ × $N_{\alpha }$ ^2^	$SR_{\alpha }$ ^3^	KEGG pathways	$10^{6}\;\times \;P_{\alpha }$ ^1^	10^5^ × $N_{\alpha }$ ^2^	$10^{3}\;\times \;SR_{\alpha }$ ^3^
Nucleotide excision repair	8.52	23.30	36.6	alpha-Linolenic acid metabolism	0	3.72	0
Sphingolipid metabolism	6.58	17.80	36.9	Photosynthesis—antenna proteins	1.30	22.30	5.83
Ubiquitin mediated proteolysis	5.30	11.80	45	Fatty acid biosynthesis	2.54	8.82	28.80
N-Glycan biosynthesis	10.30	21.90	47.3	Carotenoid biosynthesis	2.07	4.22	49.10
Base excision repair	9.49	8.16	116.0	Photosynthesis	14.9	17.40	85.60
beta-Alanine metabolism	16.90	9.19	184.0	Ribosome	4.46	3.90	114.00
Homologous recombination	9.31	2.06	452.0	Pyruvate metabolism	4.53	3.15	144.00
DNA replication	29.90	0	Inf	Lysine biosynthesis	5.84	3.25	179.00
Proteasome	7.32	0	Inf	Porphyrin and chlorophyll metabolism	10.3	4.88	211.00
SNARE interactions in vesicular transport	3.90	0	Inf	Carbon fixation in photosynthetic organisms	11.7	5.41	216.00

^1^*P_α_*: positive weight of the gene set for constraint *α* (Equation (2)), ^2^ *N_α_*: negative weight of the gene set for constraint *α* (Equation (3)), ^3^ SR (Equation (4)): set ratios reflecting the contribution of the gene set to the phenotype. See Methods for more details about the methodology.

## Supplementary Materials

The following are available online at https://www.mdpi.com/2073-4409/8/11/1367/s1. Figure S1. Measurement of acetate assimilation. Figure S2. Fatty acid classes. Figure S3. Boxplots of standard deviations. Figure S4. Comparison of 250 top-contributing genes according to surprisal analysis and K-means clustering of transcripts. Figure S5. FPKM values for transcripts Cre08.g377550 encoding a Yippee-type zinc-binding protein and *APX1* (Cre02.g087700). Table S1. Growth rates of WT and *iclC*. Table S2. Percentage of the main fatty acids of *C. reinhardtii (iclC* strain). Table S3. Number of sequenced reads for 4 acetate concentrations and 4 time points. Table S4. Variation in λ_α_ explained by the variables growth mode (stationary/exponential) and acetate. Table S5. KEGG pathways ranked from low SR or high negative sum (more stable) to high SR (less stable). Table S6. KEGG pathways ranked from low SR (stationary state) to high SR (exponential state) from the first constraint. Table S7. KEGG pathways ranked from low SR (low acetate) to high SR (high acetate) from the second constraint. Table S8. The 100 genes contributing most to the stable state including annotation. Table S9. The 100 genes contributing the most to the exponential state, including annotation. Table S10. The 100 genes contributing the most to the stationary state, including annotation. Table S11. The 100 genes contributing the most to the high-acetate state, including annotation. Table S12. The 100 genes contributing the most to the low-acetate state, including annotation. Table S13. The 100 most stable regulatory genes (Protein Kinase (PPC), transcriptional regulators, and transcription factors from the iTAK database), including annotation. Table S14. The 100 regulatory genes (Protein Kinase (PPC), transcriptional regulators, and transcription factors from iTAK) contributing the most to the stationary state, including annotation. Table S15. The 100 regulatory genes (Protein Kinase (PPC), transcriptional regulators, and transcription factors from the iTAK database) contributing the most to the exponential state, including annotation. Table S16. The 100 regulatory genes (Protein Kinase (PPC), transcriptional regulators, and transcription factors from the iTAK database) contributing the most to the low-acetate state, including annotation. Table S17. The 100 regulatory genes (Protein Kinase (PPC), transcriptional regulators, and transcription factors from the iTAK database) contributing the most to the high-acetate state, including annotation.

## Appendix A

In editing the data for surprisal analysis, all transcripts with an average FPKM value, averaged over all samples, values lower than 1 were removed because most of the noise is due to low expression values, particularly those below 1 FPKM [40]. In total, 10923 genes were kept in the data set. Values lower than 0.01 FPKM were substituted with 0.01 FPKM, to allow the computation of logarithms and expression ratios.

Surprisal analysis [12,13,14,15,16] was carried out on the natural logarithm gene expression value, where *X_i_*(*s*) is the expression level of gene *i* in sample *s*. For recent applications to the transcriptomics of *C.* *reinhardtii,* see [11,28]. The values *Y_i_*(*s*) are arranged in an *N* × *N_s_* rectangular matrix **Y**, where *N* is the number of genes and *N*_s_ the number of samples, *N>>N_s_*.

Surprisal analysis provides compaction of the data given by Equation (A1):

(A1) $$Y_{i}\;\left(s\right)=\ln X_{i}\;\left(s\right)=\ln X_{i}^{0}+\;\overset{N_{\alpha }}{\underset{\alpha =1}{\sum }}G_{i\alpha }\lambda_{\alpha }\left(s\right)$$

In Equation (1), α is the index of constraints, $N_{a}$ is the number of constraints, and $G_{i\alpha }$ is the weight of gene i in phenotype α. For a given constraint, *α*, surprisal analysis allows a factorization between the weight of the constraint, $G_{i\alpha }$, and the weight $\lambda_{\alpha }\left(s\right)$ (the Lagrange multiplier) of sample s in the phenotype α. The phenotypes, $G_{i\alpha }$, and Lagrange multipliers, $\lambda_{\alpha }\left(s\right)$, are determined via the singular value decomposition (SVD) of the matrix **Y**, as described in reference [12]:

(A2) $$G_{i\alpha }=U_{i\alpha }\;\mathrm{and}\;\lambda_{\alpha }\left(s\right)=\omega_{\alpha }V_{\alpha s}$$

where **U** and **V** are respectively the left and right eigenvectors of the **Y** matrix, as determined by the SVD procedure and ω*_α_* the singular values. The eigenvalues of the **Y** matrix are ordered by decreasing order. When all the *N_s_* terms are kept in Equation (A1) ($N_{\alpha }=\;N_{s}$), the surprisal expression of the transcript levels is an *exact* representation to the data. Usually, just a few terms in Equation (1) ($N_{\alpha }<\;N_{s}$) suffice to describe the data, which provide a determination of the main phenotypes present in the data.

The error on the Lagrange multipliers are computed as in [41]. The Lagrange multipliers, $\lambda_{\alpha }\left(s\right)$, are the weight of constraints $\alpha ,\;\alpha_{1}=0,1,2,\ldots$, for each sample *s*. Equation (1) can also be written as the following:

(A3) $$\ln X_{i}\;\left(s\right)=\ln X_{i}^{0}\;\left(p\right)+\;\underset{\alpha =1,2,\ldots }{\sum }G_{i\alpha }\;\lambda_{\alpha }\left(s\right)=\underset{\alpha =0,1,2,\ldots }{\sum }G_{i\alpha }\;\lambda_{\alpha }\;\left(s\right)$$

$X_{i}\left(p\right)$ is the level of transcript i in sample s, and α=0 is the stable state. The different vectors, $G_{\alpha }$, are the left eigenvectors obtained by the SVD decomposition of the matrix **Y** and are orthogonal for two different constraints, *α* and β:

(A4) $$\underset{i}{\sum }G_{i\alpha }G_{i\beta }=\;\delta_{\alpha ,\beta }$$

It follows from Equation (A4) that an explicit result for the weight $\lambda_{\alpha }\left(s\right)$ is the following:

(A5) $$\lambda_{\alpha }\left(s\right)=\;\underset{i}{\sum }G_{i\alpha }\ln X_{i}\left(s\right)$$

An uncertainty in the weight due to a measurement error of each transcript is the following:

(A6) $$\delta \;\lambda_{\alpha }\;\left(s\right)=\;\underset{i}{\sum }G_{i\alpha }\delta \;\left(\ln X_{i}\;\left(s\right)\right)$$

The Schwarz inequality of vector algebra yields an upper bound:

(A7) $$\delta \;\lambda_{\alpha }\left(s\right)\leq \;{\left(\overset{N}{\underset{i=1}{\sum }}G_{i\alpha }{}^{2}\right)}^{\frac{1}{2}}\;{\left(\overset{N}{\underset{i=1}{\sum }}{\left(\frac{\delta \;X_{i}^{s}}{X_{i}^{s}}\right)}^{2}\right)}^{\frac{1}{2}}$$

where $\delta \;X_{i}^{s}$ is the standard deviation obtained for the replicates of each sample. From Equation (A4), the first sum in Equation (A7) is unity. We can write the second term as av(δ X/X), and, for N = 10923, $\sqrt{N}$ = 30.38. As expected, the upper bound of the error $\delta \lambda_{\alpha }\left(s\right)$ on samples ac2t4 and particularly on ac3t4 is very large (Table A1).

**Table A1:** Upper bound of the error on the Lagrange multipliers $\lambda_{\alpha }\left(s\right)$.

Sample		
ac1t1	0.38910	40.666
ac1t2	0.14485	15.139
ac1t3	0.67040	70.066
ac1t4	0.49241	51.463
ac2t1	0.32035	33.481
ac2t2	0.18783	19.631
ac2t3	0.27087	28.309
ac2t4	0.62907	65.746
ac3t1	0.19546	20.428
ac3t2	0.20691	21.625
ac3t3	0.55731	58.246
ac3t4	0.75679	79.094
ac4t1	0.19919	20.818
ac4t2	0.19710	20.600
ac4t3	0.21022	21.971
ac4t4	0.37858	39.567
