# Supplementary material for: Metabolic, Physiological, and Transcriptomics Analysis of Batch Cultures of the Green Microalga Chlamydomonas Grown on Different Acetate Concentrations
Source: Cells. 2019 Oct 31;8(11):1367. doi: 10.3390/cells8111367 (PMC6912441; doi:10.3390/cells8111367)
Supplement: Supplementary file 1 [file cells-08-01367-s001.pdf]

**Figure S1. Measurement of acetate assimilation.** Acetate concentration (g/ L) in the medium was measured for the cells cultivated at the 4 acetate concentrations. Three biological replicates were included in the experiments. Error bars indicate standard deviations.

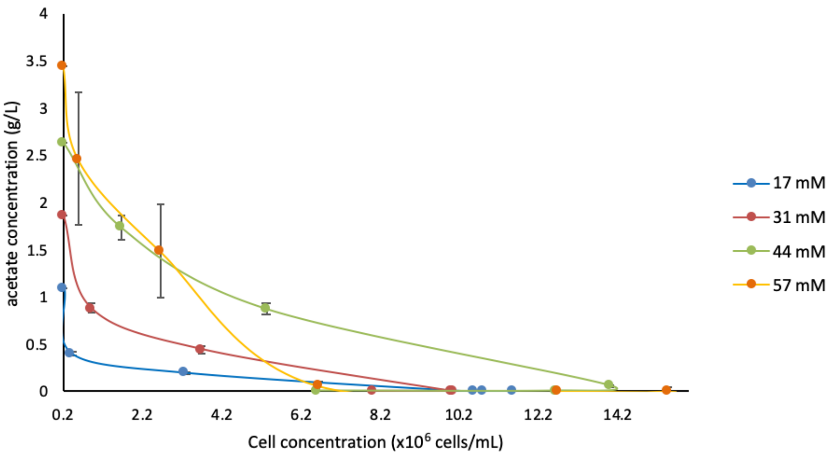

**Figure S2. Fatty acid classes.** Saturated (SAT), monounsaturated (MONOUNSAT), polyunsaturated (POLYUNSAT)

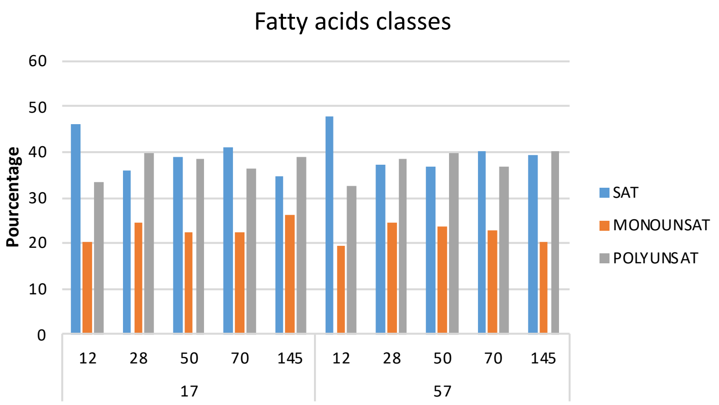

**Figure S3. Boxplots of standard deviations.** Between replicate variability of mean FPKM estimates per sample. Boxplots of log transformed standard deviation on the FPKM value per sample are depicted.

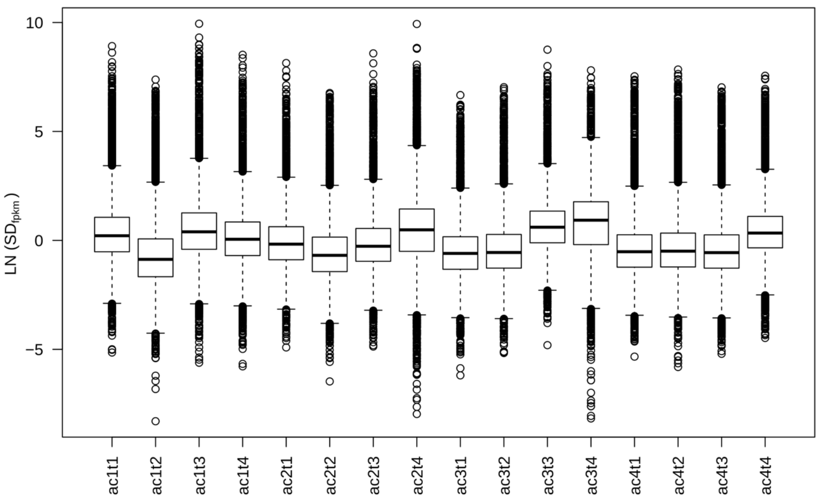

**Figure S4. Comparison of 250 top-contributing genes according to surprisal analysis and K-means clustering of transcripts.** (a) Centroid plots with mean and standard deviation of the expression values [Ln(meancenteredFPKM)] of the different genes belonging to the different clusters for each sample. Stationary-upregulated (cluster 1), high acetate (cluster 2), exponential-upregulated (cluster 3) and cluster 4. (b) Cumulative barplot describing how many of the 250 most contributing genes to the different phenotypes described according to the first 2 constraints belong to the 4 different clusters according to K-means clustering.

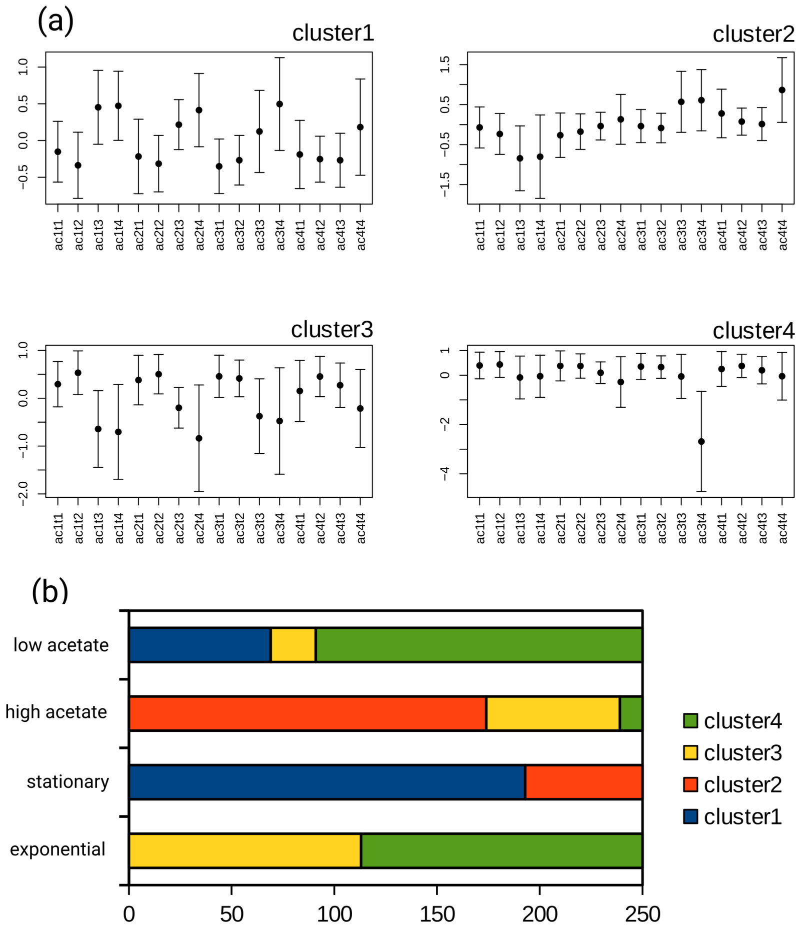

**Figure S5.** FPKM values for transcripts Cre08.g377550 encoding a Yippee-type zinc-binding protein and *APX1* (Cre02.g087700).

Formatted: Font: Italic

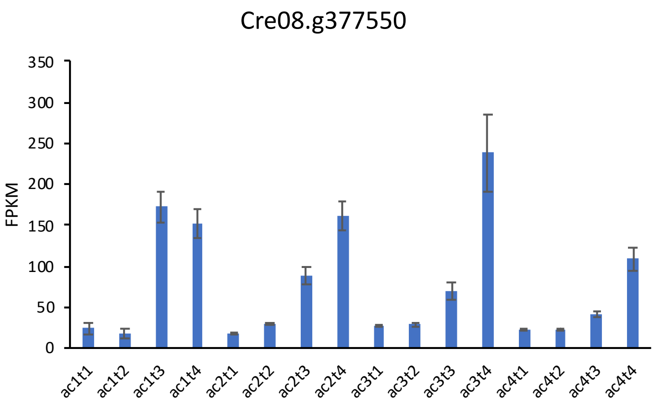


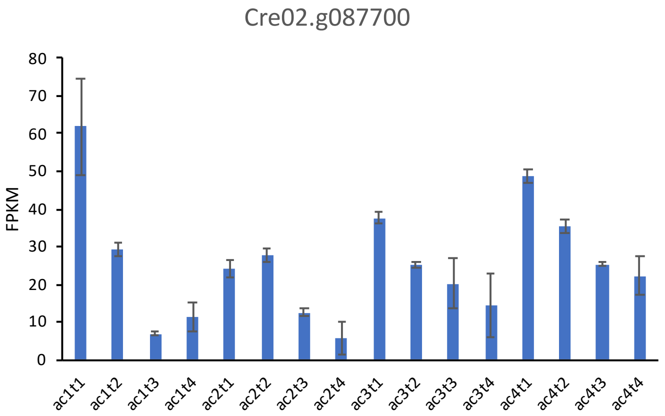

**Table S1. Growth rates of WT and *iclC*.** Number of doublings per day in exponential phase for cells cultivated in flasks at 50 µmol. $m^{-2}.s^{-1}$.

| **acetate** | **WT** | ***iclc*** |
|---|---|---|
| 17 mM | 1.9 (1.9-1.9) | 2.0 (1.8-2.3) |
| 57 mM | 1.0 (1.1-1.0) | 1.1 (1.1-1.2) |

**Table S2. Percentage of the main fatty acids of *C. reinhardtii (iclC* strain*)*.** Cells are cultivated in 17 and 57 mM acetate, and sampled at time points 12h, 28h, 50h and 70h of the growth curve (mean ± SD, on 3 biological replicates).

Formatted: Font: Not Italic

| Time point | [Acetate] (mM) | C16:0 | C16:1 | C16:3 | C18:0 | C18:1 | C18:2 | C18:3 | C20:0 |
|---|---|---|---|---|---|---|---|---|---|
| 12h | 17 | 31.7±7.7 | 3.6±0.2 | 2.2±0.7 | 15.1±5.3 | 17.3±5.7 | 7.2±0.6 | 20.3±8.6 | 2.5±1.0 |
| | 57 | 31.4±4.3 | 4.1±0.2 | 2.0±0.3 | 15.3±4.5 | 17.0±4.0 | 7.7±0.6 | 19.5±4.7 | 2.9±1.0 |
| 28h | 17 | 27.4±5.1 | 3.9±0.3 | 3.4±0.1 | 8.3±1.7 | 21.8±3.0 | 9.4±0.4 | 24.8±4.0 | 1.1±0.4 |
| | 57 | 28.1±4.7 | 3.9±0.3 | 3.0±0.3 | 8.6±0.9 | 21.8±2.7 | 9.6±0.6 | 23.9±3.7 | 1.1±0.5 |
| 50h | 17 | 25.8±4.5 | 4.8±0.4 | 3.0±0.3 | 10.2±1.1 | 20.3±1.9 | 10.5±0.8 | 22.2±2.4 | 3.3±1.2 |
| | 57 | 27.7±5.3 | 6.2±0.9 | 3.2±0.6 | 8.5±1.7 | 19.8±2.4 | 14.5±0.2 | 17.9±5.8 | 2.1±1.3 |
| 70h | 17 | 28.9±5.2 | 4.2±0.3 | 2.5±0.1 | 10.8±1.7 | 20.2±2.8 | 8.1±0.4 | 22.7±4.6 | 2.5±0.8 |
| | 57 | 32.7±5.0 | 4.8±0.6 | 3.1±0.3 | 7.3±1.5 | 20.4±3.1 | 13.5±0.2 | 17.0±4.5 | 1.3±0.5 |

**Table S3. Number of sequenced reads for 4 acetate concentrations and 4 timepoints** (ac1=17 mM, ac2=31 mM, ac3=44 mM, ac4=57 mM) and 4 times points (t1=12h, t2=28h, t3=50h, t4=70h). Samples in italics were removed from the analysis, because they had too few uniquely mapping reads to the genome. *data available in [25] (https://www.ncbi.nlm.nih.gov/Traces/study/?acc=SRP132684).

| Sample | Biological replicate | Acetate concentration (mM) | Time (hours) | Number of sequenced reads | number of trimmed reads | Number of uniquely mapped reads | Fraction of uniquely mapped reads |
|---|---|---|---|---|---|---|---|
| **ac1t1*** | 1 | 17 | 12 | 27596703 | 25600013 | 22269444 | 86.99% |
| **ac1t1*** | 2 | 17 | 12 | 30114986 | 28063350 | 22460172 | 80.03% |
| **ac1t1*** | 3 | 17 | 12 | 28741589 | 26807561 | 22575552 | 84.21% |
| **ac1t2*** | 1 | 17 | 28 | 26218763 | 24652570 | 22412128 | 90.91% |
| **ac1t2*** | 2 | 17 | 28 | 27889515 | 26249741 | 23698788 | 90.28% |
| **ac1t2*** | 3 | 17 | 28 | 26630091 | 24994681 | 22769422 | 91.10% |
| **ac1t3** | 1 | 17 | 50 | 24818061 | 22130171 | 17485954 | 79.01% |
| **ac1t3** | 2 | 17 | 50 | 26992479 | 25028973 | 22613353 | 90.35% |
| **ac1t3** | 3 | 17 | 50 | 29937141 | 27743622 | 22533450 | 81.22% |
| **ac1t4** | 1 | 17 | 70 | 28969256 | 26884722 | 15303367 | 56.92% |
| **ac1t4** | 2 | 17 | 70 | 34291726 | 31593263 | 15336658 | 48.54% |
| **ac1t4** | 3 | 17 | 70 | 28602705 | 26371812 | 14452537 | 54.80% |
| **ac2t1** | 1 | 57 | 12 | 17490336 | 14345681 | 11605784 | 64.31% |
| **ac2t1*** | 2 | 57 | 12 | 18295960 | 15367211 | 15345326 | 82.76% |
| **ac2t1*** | 3 | 57 | 12 | 17594155 | 12572740 | 15349330 | 85.50% |
| **ac2t2*** | 1 | 57 | 28 | 17636204 | 15865151 | 15595713 | 79.18% |
| **ac2t2*** | 2 | 57 | 28 | 18105831 | 16393941 | 16736724 | 90.70% |
| **ac2t2*** | 3 | 57 | 28 | 19490295 | 15448228 | 13787210 | 70.88% |
| **ac2t3** | 1 | 57 | 50 | 20236476 | 14428833 | 16153442 | 85.78% |
| **ac2t3** | 2 | 57 | 50 | 19987724 | 12434769 | 16327473 | 85.55% |
| **ac2t3** | 3 | 57 | 50 | 19455896 | 16421430 | 16842275 | 92.62% |
| **ac2t4** | 1 | 57 | 70 | 17159711 | 10394197 | 13754355 | 69.32% |
| **ac2t4** | 2 | 57 | 70 | 18376883 | 11037934 | 10688319 | 57.52% |
| ***ac2t4*** | *3* | *57* | *70* | *18042360* | *7104147* | *5663700* | *31.22%* |
| **ac3t1*** | 1 | 31 | 12 | 18047009 | 15595713 | 19335327 | 93.14% |
| **ac3t1*** | 2 | 31 | 12 | 18541383 | 16736724 | 17216882 | 91.68% |
| **ac3t1*** | 3 | 31 | 12 | 17952421 | 13787210 | 17147077 | 93.58% |
| **ac3t2*** | 1 | 31 | 28 | 19695743 | 11605784 | 16323251 | 86.84% |
| **ac3t2*** | 2 | 31 | 28 | 18453200 | 15345326 | 14403003 | 74.24% |
| **ac3t2*** | 3 | 31 | 28 | 19452219 | 15349330 | 14137670 | 75.26% |
| **ac3t3** | 1 | 31 | 50 | 18831301 | 16153442 | 10982341 | 71.86% |
| **ac3t3** | 2 | 31 | 50 | 19085569 | 16327473 | 12882313 | 71.85% |
| **ac3t3** | 3 | 31 | 50 | 18184329 | 16842275 | 16201118 | 89.03% |
| **ac3t4** | 1 | 31 | 70 | 19843257 | 13754355 | 10638497 | 81.09% |
| **ac3t4** | 2 | 31 | 70 | 18580719 | 10688319 | 6568536 | 42.59% |
| ***ac3t4*** | *3* | *31* | *70* | *18141774* | *5663700* | *3875397* | *24.90%* |
| **ac4t1*** | 1 | 44 | 12 | 20759397 | 16323251 | 15865151 | 90.71% |
| **ac4t1*** | 2 | 44 | 12 | 18779332 | 14403003 | 16393941 | 89.60% |
| **ac4t1*** | 3 | 44 | 12 | 18322514 | 14137670 | 15448228 | 87.80% |
| **ac4t2*** | 1 | 44 | 28 | 18797355 | 19335327 | 14345681 | 81.34% |
| **ac4t2*** | 2 | 44 | 28 | 19400482 | 17216882 | 15367211 | 84.87% |
| **ac4t2*** | 3 | 44 | 28 | 18784045 | 17147077 | 12572740 | 64.51% |
| **ac4t3** | 1 | 44 | 50 | 15281988 | 10982341 | 14428833 | 71.30% |
| **ac4t3** | 2 | 44 | 50 | 17929166 | 12882313 | 12434769 | 62.21% |

| | | | | | | | |
|---|---|---|---|---|---|---|---|
| **ac4t3** | 3 | 44 | 50 | 18197263 | 16201118 | 16421430 | 84.40% |
| **ac4t4** | 1 | 44 | 70 | 13119628 | 10638497 | 10394197 | 60.57% |
| **ac4t4** | 2 | 44 | 70 | 15421089 | 6568536 | 11037934 | 60.06% |
| **ac4t4** | 3 | 44 | 70 | 15565195 | 3875397 | 7104147 | 39.37% |

**Table S4. Variation in $\lambda_\alpha$ explained by the variables growth mode (stationary/exponential) and acetate.** Asterisks denote a significant relationship between $\lambda_\alpha$ and the environmental (acetate) or biological (growth mode) variable (One Way ANOVA, F-value, Df=3, P < 0.05).

| **time phenotype** | **F-statistic** | **p value** |
|---|---|---|
| Constraint 1 | 8.7678 | **0.00237*** |
| Constraint 2 | 0.0481 | 0.98536 |
| Constraint 3 | 1.3264 | 0.31160 |
| Constraint 4 | 0.5494 | 0.65808 |
| Constraint 5 | 0.8671 | 0.48483 |
| **acetate phenotype** | **statistic** | **p value** |
| Constraint 1 | 0.3747 | 0.77285 |
| Constraint 2 | 5.5548 | **0.01263*** |
| Constraint 3 | 0.3321 | 0.80238 |
| Constraint 4 | 1.4531 | 0.27647 |
| Constraint 5 | 0.6338 | 0.60733 |

**Table S5. KEGG pathways ranked from low SR or high negative sum (more stable) to high SR (less stable). Bold pathways denote top 10 stable pathways.**

| Rank Stability | names | Set size | Positive G subset | Positive sum(G²) | Negative G subsetsize | Negative sum(G²) | SR |
|---|---|---|---|---|---|---|---|
| 1 | **Oxidative phosphorylation** | 66 | 0 | 0 | 66 | 0.000355350995421 | 0 |
| 2 | **Carbon fixation in photosynthetic organisms** | 37 | 0 | 0 | 37 | 0.000332510146488 | 0 |
| 3 | **Phagosome** | 29 | 0 | 0 | 29 | 0.000316364886226 | 0 |
| 4 | **Citrate cycle (TCA cycle)** | 33 | 0 | 0 | 33 | 0.000304635822267 | 0 |
| 5 | **2-Oxocarboxylic acid metabolism** | 30 | 0 | 0 | 30 | 0.000255010881357 | 0 |
| 6 | **Valine, leucine and isoleucine biosynthesis** | 12 | 0 | 0 | 12 | 0.000242451697368 | 0 |
| 7 | **alpha-Linolenic acid metabolism** | 10 | 0 | 0 | 10 | 0.000231253920433 | 0 |
| 8 | **Lysine biosynthesis** | 10 | 0 | 0 | 10 | 0.000204109091004 | 0 |
| 9 | **Pyruvate metabolism** | 45 | 0 | 0 | 45 | 0.000195837928556 | 0 |
| 10 | **Fatty acid biosynthesis** | 19 | 0 | 0 | 19 | 0.000183795645287 | 0 |
| 11 | Phenylalanine, tyrosine and tryptophan biosynthesis | 21 | 0 | 0 | 21 | 0.000172490951702 | 0 |
| 12 | Proteasome | 34 | 0 | 0 | 34 | 0.000172007302702 | 0 |
| 13 | Fructose and mannose metabolism | 13 | 0 | 0 | 13 | 0.000155514506612 | 0 |
| 14 | Sulfur metabolism | 21 | 0 | 0 | 21 | 0.000147669898776 | 0 |
| 15 | Pentose and glucuronate interconversions | 10 | 0 | 0 | 10 | 0.000144653113935 | 0 |
| 16 | Valine, leucine and isoleucine degradation | 20 | 0 | 0 | 20 | 0.000139348141097 | 0 |
| 17 | Steroid biosynthesis | 11 | 0 | 0 | 11 | 0.000139320875242 | 0 |
| 18 | Plant hormone signal transduction | 11 | 0 | 0 | 11 | 0.000134375917837 | 0 |
| 19 | Selenocompound metabolism | 14 | 0 | 0 | 14 | 0.000130176048551 | 0 |
| 20 | Pantothenate and CoA biosynthesis | 19 | 0 | 0 | 19 | 0.000127134335054 | 0 |
| 21 | Spliceosome | 106 | 0 | 0 | 106 | 0.000123788577504 | 0 |
| 22 | Protein export | 29 | 0 | 0 | 29 | 0.000122962639836 | 0 |
| 23 | mRNA surveillance pathway | 42 | 0 | 0 | 42 | 0.000120693674893 | 0 |
| 24 | Histidine metabolism | 11 | 0 | 0 | 11 | 0.000118329890379 | 0 |
| 25 | SNARE interactions in vesicular transport | 14 | 0 | 0 | 14 | 0.00011803194921 | 0 |
| 26 | Ascorbate and aldarate metabolism | 17 | 0 | 0 | 17 | 0.000103718029108 | 0 |
| 27 | Terpenoid backbone biosynthesis | 21 | 0 | 0 | 21 | 9.80703801866992E-05 | 0 |
| 28 | Regulation of autophagy | 11 | 0 | 0 | 11 | 8.37812440043619E-05 | 0 |
| 29 | Aminoacyl-tRNA biosynthesis | 42 | 0 | 0 | 42 | 8.17559119495921E-05 | 0 |
| 30 | Carotenoid biosynthesis | 12 | 0 | 0 | 12 | 7.02912559110478E-05 | 0 |
| 31 | Nucleotide excision repair | 30 | 0 | 0 | 30 | 4.58930871398967E-05 | 0 |
| 32 | Mismatch repair | 17 | 0 | 0 | 17 | 4.39849088741505E-05 | 0 |
| 33 | Base excision repair | 13 | 0 | 0 | 13 | 4.34949898251257E-05 | 0 |
| 34 | Glycerolipid metabolism | 23 | 1 | 3.5097645464952E-12 | 22 | 6.45336431669079E-05 | 5.43865861937725E-08 |
| 35 | Inositol phosphate metabolism | 24 | 1 | 5.8689138694192E-12 | 23 | 7.79941753971541E-05 | 7.52481046120958E-08 |
| 36 | Phosphatidylinositol signaling system | 35 | 2 | 6.34994573690113E-12 | 33 | 8.38130119063243E-05 | 7.57632447811124E-08 |
| 37 | Fatty acid metabolism | 35 | 1 | 1.35100799446707E-10 | 34 | 0.000180398860024 | 7.48900516492723E-07 |
| 38 | Biosynthesis of unsaturated fatty acids | 16 | 1 | 3.2086439868593E-10 | 15 | 0.000196849537302 | 1.62999823664201E-06 |
| 39 | Nicotinate and nicotinamide metabolism | 17 | 1 | 2.61011084719169E-10 | 16 | 8.06066179301011E-05 | 3.23808505333281E-06 |
| 40 | N-Glycan biosynthesis | 20 | 1 | 2.11220645643598E-10 | 19 | 6.46800031775959E-05 | 3.26562515873161E-06 |
| 41 | Ribosome | 124 | 1 | 4.72303160637398E-09 | 123 | 0.000866811096156 | 5.44874382356074E-06 |
| 42 | Carbon metabolism | 131 | 2 | 5.70294205970845E-09 | 129 | 0.000235277792368 | 2.42391855274987E-05 |
| 43 | Propanoate metabolism | 18 | 1 | 5.54414498365616E-09 | 17 | 0.000183457568984 | 3.02203120555556E-05 |
| 44 | Basal transcription factors | 20 | 1 | 1.46466437276582E-09 | 19 | 4.77283335166987E-05 | 3.06875238426955E-05 |
| 45 | Photosynthesis - antenna proteins | 23 | 1 | 2.02441726380731E-08 | 22 | 0.000460832981165 | 4.39295221164656E-05 |
| 46 | Protein processing in endoplasmic reticulum | 80 | 3 | 6.27418290808014E-09 | 77 | 0.000134897169935 | 4.65108564627932E-05 |
| 47 | Arachidonic acid metabolism | 10 | 1 | 3.33546317662889E-09 | 9 | 6.41143784123809E-05 | 5.20236374308324E-05 |

| | | | | | | | |
|---|---|---|---|---|---|---|---|
| 48 | RNA transport | 89 | 1 | 7.45691588374356E-09 | 88 | 0.00013141471952 | 5.67433839299559E-05 |
| 49 | Ubiquitin mediated proteolysis | 54 | 1 | 5.12329571780646E-09 | 53 | 8.225902918128E-05 | 6.22824724385683E-05 |
| 50 | RNA degradation | 53 | 1 | 5.41173505621507E-09 | 52 | 8.16377759907414E-05 | 6.62895943763683E-05 |
| 51 | Peroxisome | 47 | 4 | 1.66419929907805E-08 | 43 | 0.000154269100457 | 0.000107876385754 |
| 52 | Endocytosis | 53 | 3 | 1.25609275370062E-08 | 50 | 0.000115238113204 | 0.000108999767418 |
| 53 | Porphyrin and chlorophyll metabolism | 40 | 1 | 1.18867896458742E-08 | 39 | 0.000104156816259 | 0.00011412397261 |
| 54 | Fatty acid degradation | 17 | 1 | 2.33817017604982E-08 | 16 | 0.000198615854243 | 0.000117723239414 |
| 55 | Ribosome biogenesis in eukaryotes | 57 | 1 | 1.16132296550105E-08 | 56 | 9.63850864350657E-05 | 0.000120487827366 |
| 56 | Glutathione metabolism | 32 | 2 | 2.15000277517205E-08 | 30 | 0.000118117246006 | 0.000182022765334 |
| 57 | Biosynthesis of amino acids | 120 | 1 | 4.11713195458188E-08 | 119 | 0.000195681099858 | 0.000210400082459 |
| 58 | Arginine and proline metabolism | 32 | 1 | 3.65316803913018E-08 | 31 | 0.00013282595145 | 0.000275034208243 |
| 59 | Glycolysis / Gluconeogenesis | 45 | 2 | 6.34994237357837E-08 | 43 | 0.000203203255585 | 0.000312492157436 |
| 60 | Pentose phosphate pathway | 24 | 1 | 1.0438395512019E-07 | 23 | 0.000217727580251 | 0.000479424586448 |
| 61 | Glyoxylate and dicarboxylate metabolism | 36 | 2 | 1.51040789762267E-07 | 34 | 0.000311460980794 | 0.000484942895181 |
| 62 | Amino sugar and nucleotide sugar metabolism | 36 | 2 | 7.34055378678352E-08 | 34 | 0.00013798279026 | 0.000531990531062 |
| 63 | Alanine, aspartate and glutamate metabolism | 27 | 1 | 1.92132824547154E-07 | 26 | 0.000234687309359 | 0.000818675816225 |
| 64 | Homologous recombination | 13 | 1 | 3.53177467327263E-08 | 12 | 4.09222528783088E-05 | 0.000863045024372 |
| 65 | One carbon pool by folate | 14 | 1 | 1.20974535093299E-07 | 13 | 0.000126319251077 | 0.000957688824643 |
| 66 | RNA polymerase | 26 | 1 | 7.88033409662853E-08 | 25 | 6.91206363966133E-05 | 0.001140084135136 |
| 67 | Pyrimidine metabolism | 76 | 4 | 1.06043114944306E-07 | 72 | 9.00796388985311E-05 | 0.001177215142522 |
| 68 | Starch and sucrose metabolism | 41 | 4 | 1.55042790314208E-07 | 37 | 0.000125609029266 | 0.001234328385624 |
| 69 | Sphingolipid metabolism | 15 | 1 | 7.37314758910123E-08 | 14 | 4.91164076160397E-05 | 0.001501157748901 |
| 70 | Arginine biosynthesis | 21 | 2 | 3.09653655088573E-07 | 19 | 0.000201555016631 | 0.001536323234539 |
| 71 | Glycerophospholipid metabolism | 32 | 2 | 1.32723750078467E-07 | 30 | 6.10950457180683E-05 | 0.002172414285291 |
| 72 | ABC transporters | 12 | 2 | 9.2529112951528E-08 | 10 | 3.54705573915424E-05 | 0.002608617393015 |
| 73 | Purine metabolism | 101 | 8 | 2.48508622430845E-07 | 93 | 8.95674656925117E-05 | 0.002774541185345 |
| 74 | **Photosynthesis** | 33 | 2 | 1.63228474516247E-06 | 31 | 0.000519266443881 | 0.003143443533466 |
| 75 | **Galactose metabolism** | 13 | 2 | 4.33469468910448E-07 | 11 | 9.41729568103144E-05 | 0.004602908134058 |
| 76 | **Ubiquinone and other terpenoid-quinone biosynthesis** | 17 | 2 | 4.14602087947335E-07 | 15 | 6.85879235348066E-05 | 0.006044826356887 |
| 77 | **DNA replication** | 28 | 4 | 3.30550443727272E-07 | 24 | 3.53124674172003E-05 | 0.009360729167463 |
| 78 | **Nitrogen metabolism** | 10 | 2 | 1.65656726365839E-06 | 8 | 0.000154399166856 | 0.010729120482935 |
| 79 | **Glycine, serine and threonine metabolism** | 35 | 1 | 2.83265308139074E-06 | 34 | 0.000158284511702 | 0.017895958681777 |
| 80 | **Cysteine and methionine metabolism** | 42 | 2 | 3.33441854992681E-06 | 40 | 0.000172904498725 | 0.019284741429649 |
| 81 | **Tyrosine metabolism** | 14 | 2 | 6.92943494401046E-06 | 12 | 0.000110182223635 | 0.062890679779266 |
| 82 | **Phenylalanine metabolism** | 10 | 1 | 9.20612251451991E-06 | 9 | 0.000131671732299 | 0.069917227895429 |
| 83 | **beta-Alanine metabolism** | 12 | 2 | 9.21490074407737E-06 | 10 | 0.000118507289164 | 0.077758092426824 |

**Table S6. KEGG pathways ranked from low SR (stationary state) to high SR (exponential state) from the first constraint.** Bold pathways denote the top 10 pathways contributing the most to either state. SR values <0.5 or >2 (grey background) indicate KEGG pathways where the sum of the positive contributions ($G^2$) of containing genes are at least double or less than half of the sum of the negative contributions.

| Rank Stationary | Rank Exponential | names | Set size | Positive G subset | Positive sum($G^2$) | Negative G subsetsize | Negative sum($G^2$) | SR |
|---|---|---|---|---|---|---|---|---|
| 1 | 83 | Regulation of autophagy | 11 | 0 | 0 | 11 | 7.7566114627055E-05 | 0 |
| 2 | 82 | Valine, leucine and isoleucine degradation | 20 | 5 | 5.54342207234786E-06 | 15 | 5.57378531207342E-05 | 0.09945524920632 |
| 3 | 81 | Endocytosis | 53 | 17 | 1.87791840126372E-06 | 36 | 1.37474291350847E-05 | 0.136601424368946 |
| 4 | 80 | Plant hormone signal transduction | 11 | 4 | 1.92311671378083E-06 | 7 | 1.36469201698926E-05 | 0.140919466798344 |
| 5 | 79 | Peroxisome | 47 | 8 | 8.77502736124042E-06 | 39 | 3.62611942208702E-05 | 0.241994990782458 |
| 6 | 78 | Propanoate metabolism | 18 | 6 | 1.44650270373447E-05 | 12 | 4.60629635148019E-05 | 0.314027277743355 |
| 7 | 77 | beta-Alanine metabolism | 12 | 3 | 2.08791962473789E-05 | 9 | 5.69587536722822E-05 | 0.366566943643279 |
| 8 | 76 | SNARE interactions in vesicular transport | 14 | 5 | 6.49822069890119E-06 | 9 | 1.46704504769261E-05 | 0.442946227801367 |
| 9 | 75 | **Selenocompound metabolism** | 14 | 5 | 2.03738375099447E-05 | 9 | 3.73587275321166E-05 | 0.54535683776782 |
| 10 | 74 | **Pentose and glucuronate interconversions** | 10 | 4 | 7.64833874271331E-06 | 6 | 1.24103030098983E-05 | 0.616289444070226 |
| 11 | 73 | alpha-Linolenic acid metabolism | 10 | 2 | 1.44060522561005E-05 | 8 | 1.90411801952735E-05 | 0.756573495359099 |
| 12 | 72 | Cysteine and methionine metabolism | 42 | 25 | 3.64701686794292E-05 | 17 | 4.11246031796287E-05 | 0.886821169316352 |
| 13 | 71 | Base excision repair | 13 | 6 | 1.46887558288418E-05 | 7 | 1.54808386346216E-05 | 0.948834631994138 |
| 14 | 70 | Ribosome | 124 | 42 | 2.96973606304024E-05 | 82 | 2.98595361187222E-05 | 0.994568720435743 |
| 15 | 69 | Glyoxylate and dicarboxylate metabolism | 36 | 16 | 1.1915428378493E-05 | 20 | 1.19625489466162E-05 | 0.996060992658552 |
| 16 | 68 | Fatty acid degradation | 17 | 2 | 3.0847376956461E-05 | 15 | 3.07043819700765E-05 | 1.00465715240658 |
| 17 | 67 | Arginine and proline metabolism | 32 | 10 | 3.2666908337155E-05 | 22 | 2.76203486283993E-05 | 1.18271165859097 |
| 18 | 66 | Ubiquitin mediated proteolysis | 54 | 17 | 2.60044362425567E-05 | 37 | 1.74108892173597E-05 | 1.49357312644484 |
| 19 | 65 | Starch and sucrose metabolism | 41 | 16 | 3.35644223717635E-05 | 25 | 2.18872178100128E-05 | 1.53351708120749 |
| 20 | 64 | Pyruvate metabolism | 45 | 27 | 2.68270534836927E-05 | 18 | 1.57083171023985E-05 | 1.70782479808715 |
| 21 | 63 | Galactose metabolism | 13 | 9 | 7.90391604290231E-05 | 4 | 3.92662146792397E-05 | 2.01290501451905 |
| 22 | 62 | Tyrosine metabolism | 14 | 9 | 0.000118426799386 | 5 | 5.50685574518761E-05 | 2.15053389567942 |
| 23 | 61 | Valine, leucine and isoleucine biosynthesis | 12 | 6 | 1.87433765392686E-05 | 6 | 7.95943819599509E-06 | 2.35486174749113 |
| 24 | 60 | Arachidonic acid metabolism | 10 | 5 | 6.64840530608746E-05 | 5 | 2.5064580856565E-05 | 2.65251006754661 |
| 25 | 59 | Nitrogen metabolism | 10 | 3 | 3.22038383352851E-05 | 7 | 1.18469954621349E-05 | 2.71831270959917 |
| 26 | 58 | ABC transporters | 12 | 6 | 7.45209220923073E-05 | 6 | 2.61099901604096E-05 | 2.85411528822798 |
| 27 | 57 | Ribosome biogenesis in eukaryotes | 57 | 32 | 2.433559720558E-05 | 25 | 8.51607186942815E-06 | 2.85760824693629 |
| 28 | 56 | Fructose and mannose metabolism | 13 | 10 | 2.76752976509033E-05 | 3 | 9.58767394836722E-06 | 2.88654973040843 |
| 29 | 55 | Phagosome | 29 | 19 | 1.61703872464896E-05 | 10 | 5.5922498850236E-06 | 2.89157093816479 |
| 30 | 54 | Alanine, aspartate and glutamate metabolism | 27 | 13 | 4.77656226486358E-05 | 14 | 1.62093398091941E-05 | 2.94679630453196 |
| 31 | 53 | Carbon fixation in photosynthetic organisms | 37 | 23 | 5.96445524354843E-05 | 14 | 1.9737212234638E-05 | 3.02193398573333 |
| 32 | 52 | Glycerophospholipid metabolism | 32 | 17 | 4.86915715689258E-05 | 15 | 1.60794985090323E-05 | 3.0281772495314 |
| 33 | 51 | Nucleotide excision repair | 30 | 13 | 2.08062507113927E-05 | 17 | 6.75041590676193E-06 | 3.08221759944465 |
| 34 | 50 | mRNA surveillance pathway | 42 | 27 | 1.81637909145652E-05 | 15 | 5.71911185339432E-06 | 3.17598105793033 |
| 35 | 49 | Phenylalanine metabolism | 10 | 6 | 8.88670517134741E-05 | 4 | 2.73532745278459E-05 | 3.24886337184261 |
| 36 | 48 | 2-Oxocarboxylic acid metabolism | 30 | 13 | 3.15678884257914E-05 | 17 | 9.03612529045881E-06 | 3.49352044278578 |
| 37 | 47 | Pantothenate and CoA biosynthesis | 19 | 6 | 6.74461193408085E-05 | 13 | 1.91458628188293E-05 | 3.52275162415129 |
| 38 | 46 | Citrate cycle (TCA cycle) | 33 | 16 | 2.92184470137691E-05 | 17 | 8.26915338333957E-06 | 3.53342665920764 |
| 39 | 45 | Mismatch repair | 17 | 6 | 2.04404535571122E-05 | 11 | 5.4584051943244E-06 | 3.74476661761314 |
| 40 | 44 | Fatty acid biosynthesis | 19 | 12 | 4.06595622686739E-05 | 7 | 1.04501746664177E-05 | 3.89080216997098 |
| 41 | 43 | One carbon pool by folate | 14 | 9 | 7.84068716710907E-05 | 5 | 1.93900986805211E-05 | 4.04365511300139 |
| 42 | 42 | Glycerolipid metabolism | 23 | 11 | 9.19121709764475E-05 | 12 | 2.22041889575659E-05 | 4.13940680977357 |
| 43 | 41 | Fatty acid metabolism | 35 | 15 | 6.41467760254326E-05 | 20 | 1.47509476984419E-05 | 4.3486545635443 |
| 44 | 40 | Protein processing in endoplasmic reticulum | 80 | 45 | 4.57048539672092E-05 | 35 | 1.02469388983873E-05 | 4.46034219784431 |
| 45 | 39 | Phenylalanine, tyrosine and tryptophan biosynthesis | 21 | 16 | 6.74649165981001E-05 | 5 | 1.46782499143288E-05 | 4.596250710532 |

| | | | | | | | | |
|---|---|---|---|---|---|---|---|---|
| 46 | 38 | Carbon metabolism | 131 | 77 | 5.11354114806906E-05 | 54 | 1.09875501657145E-05 | 4.6539411160327 |
| 47 | 37 | Glutathione metabolism | 32 | 18 | 9.77371293331979E-05 | 14 | 2.07595905937399E-05 | 4.70804705381189 |
| 48 | 36 | Glycine, serine and threonine metabolism | 35 | 21 | 5.27812076571822E-05 | 14 | 1.07852587780657E-05 | 4.89382858059232 |
| 49 | 35 | RNA transport | 89 | 55 | 4.04787482994702E-05 | 34 | 7.95713290432183E-06 | 5.08710219952271 |
| 50 | 34 | Basal transcription factors | 20 | 11 | 4.96816583055217E-05 | 9 | 9.41328342735687E-06 | 5.27782454325521 |
| 51 | 33 | Arginine biosynthesis | 21 | 11 | 7.8116200179021E-05 | 10 | 1.37565966525879E-05 | 5.67845391936569 |
| 52 | 32 | Spliceosome | 106 | 72 | 3.50500733094166E-05 | 34 | 5.42196367938373E-06 | 6.46446110339871 |
| 53 | 31 | Purine metabolism | 101 | 62 | 6.8441892104463E-05 | 39 | 1.03861508112397E-05 | 6.58972639126289 |
| 54 | 30 | Biosynthesis of unsaturated fatty acids | 16 | 6 | 0.000100095772727 | 10 | 1.41556367045121E-05 | 7.07108940531341 |
| 55 | 29 | RNA degradation | 53 | 30 | 5.88916489082065E-05 | 23 | 8.06309370527E-06 | 7.30385272215244 |
| 56 | 28 | Nicotinate and nicotinamide metabolism | 17 | 9 | 7.96574468720383E-05 | 8 | 1.06647231774838E-05 | 7.46924655674304 |
| 57 | 27 | Proteasome | 34 | 24 | 6.54707102457124E-06 | 10 | 8.10772959540296E-07 | 8.07509790198652 |
| 58 | 26 | Homologous recombination | 13 | 6 | 5.34300669218936E-05 | 7 | 5.80274998408841E-06 | 9.20771480218914 |
| 59 | 25 | Inositol phosphate metabolism | 24 | 12 | 0.000151634631205 | 12 | 1.62994204805232E-05 | 9.30306886590089 |
| 60 | 24 | Amino sugar and nucleotide sugar metabolism | 36 | 21 | 8.87686991839296E-05 | 15 | 8.33788635932352E-06 | 10.646427086964 |
| 61 | 23 | Oxidative phosphorylation | 66 | 47 | 2.45507724794666E-05 | 19 | 2.21578396397889E-06 | 11.0799486225095 |
| 62 | 22 | Pyrimidine metabolism | 76 | 45 | 7.86468694260353E-05 | 31 | 6.97415119086303E-06 | 11.2769091569268 |
| 63 | 21 | Aminoacyl-tRNA biosynthesis | 42 | 34 | 7.59466408182134E-05 | 8 | 6.30445596291615E-06 | 12.0465019130824 |
| 64 | 20 | Sphingolipid metabolism | 15 | 8 | 9.53524929417924E-05 | 7 | 7.90159321503573E-06 | 12.0675021286022 |
| 65 | 19 | Phosphatidylinositol signaling system | 35 | 19 | 0.000111135921137 | 16 | 9.13587189491506E-06 | 12.1647854102092 |
| 66 | 18 | Biosynthesis of amino acids | 120 | 79 | 8.44770130293984E-05 | 41 | 6.34289640461107E-06 | 13.3183655605642 |
| 67 | 17 | Ascorbate and aldarate metabolism | 17 | 10 | 9.19563005787522E-05 | 7 | 6.43172251340224E-06 | 14.2973053310581 |
| 68 | 16 | Protein export | 29 | 22 | 4.78269360695944E-05 | 7 | 3.17830168812098E-06 | 15.047953518179 |
| 69 | 15 | Sulfur metabolism | 21 | 14 | 6.37188166222858E-05 | 7 | 4.02922526450064E-06 | 15.8141608968052 |
| 70 | 14 | Pentose phosphate pathway | 24 | 19 | 0.000106860728166 | 5 | 5.75543464795044E-06 | 18.5669258192087 |
| 71 | 13 | Glycolysis / Gluconeogenesis | 45 | 34 | 9.47992236237634E-05 | 11 | 4.58773195553766E-06 | 20.6636360935027 |
| 72 | 12 | Terpenoid backbone biosynthesis | 21 | 14 | 0.000131423957674 | 7 | 6.30495258837856E-06 | 20.8445592305032 |
| 73 | 11 | Steroid biosynthesis | 11 | 8 | 0.000151371825159 | 3 | 6.59698999615227E-06 | 22.9455896169092 |
| 74 | 10 | **Porphyrin and chlorophyll metabolism** | 40 | 31 | 0.00023502101665 | 9 | 9.80016265223729E-06 | 23.9813383705813 |
| 75 | 9 | **DNA replication** | 28 | 15 | 9.61736514076376E-05 | 13 | 3.92971382633096E-06 | 24.4734491257934 |
| 76 | 8 | **Carotenoid biosynthesis** | 12 | 11 | 4.81795322217881E-05 | 1 | 1.79159484444206E-06 | 26.8919797192162 |
| 77 | 7 | **N-Glycan biosynthesis** | 20 | 16 | 0.000104388928645 | 4 | 3.69991613604256E-06 | 28.2138634517583 |
| 78 | 6 | **RNA polymerase** | 26 | 18 | 5.85100042637253E-05 | 8 | 1.77798477333666E-06 | 32.908045749977 |
| 79 | 5 | **Ubiquinone and other terpenoid-quinone biosynthesis** | 17 | 13 | 0.000183713857725 | 4 | 5.05361047219889E-06 | 36.3529913387764 |
| 80 | 4 | **Lysine biosynthesis** | 10 | 8 | 0.00014362695846 | 2 | 3.68935052376957E-06 | 38.9301470637196 |
| 81 | 3 | **Histidine metabolism** | 11 | 6 | 0.000146351790137 | 5 | 3.33003018761552E-06 | 43.9490881137679 |
| 82 | 2 | **Photosynthesis - antenna proteins** | 23 | 22 | 0.000942091209597 | 1 | 2.22239770512546E-07 | 4239.07569479725 |
| 83 | 1 | **Photosynthesis** | 33 | 32 | 0.000484202173356 | 1 | 9.36900361348169E-09 | 51681.2879290016 |

**Table S7. KEGG pathways ranked from low SR (low acetate) to high SR (high acetate) from the second constraint.** Bold pathways denote the top 10 pathways contributing the most to either state. SR values <0.5 or >2 (grey background) indicate KEGG pathways where the sum of the positive contributions ($G^2$) of containing genes are at least double or less than half of the sum of the negative contributions.

| Rank Low Acetate | Rank High acetate | names | Set size | Positive G subset | Positive sum($G^2$) | Negative G subsetsize | Negative sum($G^2$) | SR |
|---|---|---|---|---|---|---|---|---|
| 1 | 83 | **alpha-Linolenic acid metabolism** | 10 | 0 | 0 | 10 | 3.71606353666472E-05 | 0 |
| 2 | 82 | **Photosynthesis - antenna proteins** | 23 | 1 | 1.29977127077725E-06 | 22 | 0.000222967613709 | 0.005829417327284 |
| 3 | 81 | **Fatty acid biosynthesis** | 19 | 4 | 2.53568479025119E-06 | 15 | 8.81899569903032E-05 | 0.02875253460584 |
| 4 | 80 | **Carotenoid biosynthesis** | 12 | 3 | 2.07406766610321E-06 | 9 | 4.22282095973124E-05 | 0.049115690337845 |
| 5 | 79 | **Photosynthesis** | 33 | 2 | 1.4921138092381E-05 | 31 | 0.000174360180435 | 0.085576523579996 |
| 6 | 78 | **Ribosome** | 124 | 19 | 4.45724858226991E-06 | 105 | 3.89963681019487E-05 | 0.114299069354799 |
| 7 | 77 | **Pyruvate metabolism** | 45 | 17 | 4.53309118929998E-06 | 28 | 3.14976778080882E-05 | 0.143918266512204 |
| 8 | 76 | **Lysine biosynthesis** | 10 | 3 | 5.84030696034789E-06 | 7 | 3.25485714319654E-05 | 0.179433588124001 |
| 9 | 75 | **Porphyrin and chlorophyll metabolism** | 40 | 17 | 1.0297009733194E-05 | 23 | 4.88237249390238E-05 | 0.210901764378978 |
| 10 | 74 | **Carbon fixation in photosynthetic organisms** | 37 | 9 | 1.17010884172259E-05 | 28 | 5.41218403961604E-05 | 0.216199011925249 |
| 11 | 73 | Fatty acid metabolism | 35 | 13 | 1.32076669015663E-05 | 22 | 6.00524708061381E-05 | 0.21993544519098 |
| 12 | 72 | Regulation of autophagy | 11 | 6 | 4.25400853413254E-06 | 5 | 1.86755847317052E-05 | 0.227784489495025 |
| 13 | 71 | Propanoate metabolism | 18 | 8 | 1.1932998985184E-05 | 10 | 4.60004111880338E-05 | 0.259410702578419 |
| 14 | 70 | Biosynthesis of unsaturated fatty acids | 16 | 6 | 1.73401497461809E-05 | 10 | 4.40435833069922E-05 | 0.393704336573088 |
| 15 | 69 | Terpenoid backbone biosynthesis | 21 | 9 | 1.6001447923201E-05 | 12 | 3.31754678620265E-05 | 0.482327724502619 |
| 16 | 68 | Fatty acid degradation | 17 | 6 | 6.89061770839154E-06 | 11 | 1.41958523488842E-05 | 0.485396546754951 |
| 17 | 67 | Carbon metabolism | 131 | 55 | 1.98249280513014E-05 | 76 | 3.64555133915484E-05 | 0.543811517297064 |
| 18 | 66 | Nicotinate and nicotinamide metabolism | 17 | 10 | 1.73595362729729E-05 | 7 | 2.93785618483568E-05 | 0.590891288776406 |
| 19 | 65 | Ribosome biogenesis in eukaryotes | 57 | 21 | 8.8664405149457E-06 | 36 | 1.33819706782274E-05 | 0.662566129319914 |
| 20 | 64 | Valine, leucine and isoleucine degradation | 20 | 7 | 1.43168479624328E-05 | 13 | 1.96903915699469E-05 | 0.727098184491384 |
| 21 | 63 | Inositol phosphate metabolism | 24 | 13 | 1.00497919583491E-05 | 11 | 1.30826127100369E-05 | 0.768179275890273 |
| 22 | 62 | Glyoxylate and dicarboxylate metabolism | 36 | 12 | 2.64156194001291E-05 | 24 | 3.26242869472674E-05 | 0.809691854501715 |
| 23 | 61 | Glycerolipid metabolism | 23 | 11 | 1.82161364409318E-05 | 12 | 2.15286708449432E-05 | 0.8461338171841 |
| 24 | 60 | Pentose phosphate pathway | 24 | 11 | 4.05101575742417E-05 | 13 | 4.35952728686872E-05 | 0.929232802287117 |
| 25 | 59 | Oxidative phosphorylation | 66 | 38 | 1.2654558380013E-05 | 28 | 1.33415234842045E-05 | 0.948509245963943 |
| 26 | 58 | Citrate cycle (TCA cycle) | 33 | 15 | 1.73802258623453E-05 | 18 | 1.72334562041166E-05 | 1.00851655387581 |
| 27 | 57 | Phosphatidylinositol signaling system | 35 | 18 | 1.25378475447452E-05 | 17 | 1.22614055535686E-05 | 1.02254570163012 |
| 28 | 56 | Glycolysis / Gluconeogenesis | 45 | 20 | 3.34775825552621E-05 | 25 | 3.26922069801772E-05 | 1.02402332689136 |
| 29 | 55 | Glycine, serine and threonine metabolism | 35 | 15 | 3.18792776785898E-05 | 20 | 2.98115862446294E-05 | 1.06935865193463 |
| 30 | 54 | Peroxisome | 47 | 21 | 2.59107706126714E-05 | 26 | 2.40176979736783E-05 | 1.07881990360057 |
| 31 | 53 | Steroid biosynthesis | 11 | 5 | 9.19795390821098E-06 | 6 | 7.60473907550286E-06 | 1.20950289245825 |
| 32 | 52 | Histidine metabolism | 11 | 4 | 1.79318496103497E-05 | 7 | 1.44540798585274E-05 | 1.24060817332281 |
| 33 | 51 | Valine, leucine and isoleucine biosynthesis | 12 | 5 | 1.50461873422053E-05 | 7 | 1.10567439275E-05 | 1.36081539383244 |
| 34 | 50 | Phenylalanine, tyrosine and tryptophan biosynthesis | 21 | 7 | 1.93802677188277E-05 | 14 | 1.39722997860493E-05 | 1.38704923424117 |
| 35 | 49 | Ubiquinone and other terpenoid-quinone biosynthesis | 17 | 9 | 1.84486663681333E-05 | 8 | 1.26749002869749E-05 | 1.4555275347682 |
| 36 | 48 | Basal transcription factors | 20 | 13 | 9.56108494399651E-06 | 7 | 6.02392488322312E-06 | 1.58718528689236 |
| 37 | 47 | Alanine, aspartate and glutamate metabolism | 27 | 15 | 4.65416897560201E-05 | 12 | 2.81035735751866E-05 | 1.65607728253865 |
| 38 | 46 | Biosynthesis of amino acids | 120 | 54 | 3.8702745120734E-05 | 66 | 2.10845192275101E-05 | 1.83560007715217 |
| 39 | 45 | RNA degradation | 53 | 36 | 1.98840269097261E-05 | 17 | 1.03138538558489E-05 | 1.92789496415542 |
| 40 | 44 | Tyrosine metabolism | 14 | 7 | 4.39964781414467E-05 | 7 | 2.20922920315559E-05 | 1.99148545015626 |
| 41 | 43 | Fructose and mannose metabolism | 13 | 7 | 7.84342169885776E-05 | 6 | 3.46627928036226E-05 | 2.2627783465959 |
| 42 | 42 | Phenylalanine metabolism | 10 | 5 | 5.79014643844681E-05 | 5 | 2.30607313402472E-05 | 2.5108251568507 |
| 43 | 41 | One carbon pool by folate | 14 | 7 | 4.89636967101774E-05 | 7 | 1.85169729363939E-05 | 2.64426031610937 |
| 44 | 40 | Aminoacyl-tRNA biosynthesis | 42 | 26 | 1.51209291839449E-05 | 16 | 5.61174390936557E-06 | 2.69451518603855 |

| | | | | | | | | |
|---|---|---|---|---|---|---|---|---|
| 45 | 39 | Nitrogen metabolism | 10 | 5 | 5.22351166445619E-05 | 5 | 1.83810312611197E-05 | 2.84179466878182 |
| 46 | 38 | Ascorbate and aldarate metabolism | 17 | 11 | 6.57964858240712E-05 | 6 | 1.92975801577917E-05 | 3.40957183678311 |
| 47 | 37 | Selenocompound metabolism | 14 | 6 | 2.58646413187865E-05 | 8 | 6.70376042067926E-06 | 3.85822876948305 |
| 48 | 36 | Protein processing in endoplasmic reticulum | 80 | 61 | 6.86595453139012E-05 | 19 | 1.74238374181285E-05 | 3.94055245502146 |
| 49 | 35 | 2-Oxocarboxylic acid metabolism | 30 | 16 | 3.66002783870423E-05 | 14 | 8.87999674118398E-06 | 4.12165448409414 |
| 50 | 34 | Purine metabolism | 101 | 71 | 7.57549990959627E-05 | 30 | 1.75600313383829E-05 | 4.31405830867606 |
| 51 | 33 | Starch and sucrose metabolism | 41 | 24 | 8.60466687377767E-05 | 17 | 1.70989174039547E-05 | 5.03228752469881 |
| 52 | 32 | Pyrimidine metabolism | 76 | 57 | 7.79230072202996E-05 | 19 | 1.49822221170585E-05 | 5.20103136981114 |
| 53 | 31 | Arginine and proline metabolism | 32 | 21 | 5.01546437693255E-05 | 11 | 9.47920084455454E-06 | 5.29102026550451 |
| 54 | 30 | Cysteine and methionine metabolism | 42 | 23 | 7.7751811058141E-05 | 19 | 1.42095712137113E-05 | 5.4717915050889 |
| 55 | 29 | Protein export | 29 | 20 | 4.62900354431182E-05 | 9 | 8.24128646775005E-06 | 5.61684581943137 |
| 56 | 28 | RNA polymerase | 26 | 22 | 3.03102824124316E-05 | 4 | 5.23314425209737E-06 | 5.7919829747257 |
| 57 | 27 | Spliceosome | 106 | 85 | 2.10129752054405E-05 | 21 | 3.41578562480917E-06 | 6.15172540478572 |
| 58 | 26 | Sulfur metabolism | 21 | 12 | 5.59796181839274E-05 | 9 | 8.69441657830765E-06 | 6.43857097020117 |
| 59 | 25 | Arachidonic acid metabolism | 10 | 6 | 7.70841630425342E-05 | 4 | 1.13588487920981E-05 | 6.78626544409663 |
| 60 | 24 | Arginine biosynthesis | 21 | 14 | 0.000141834094132 | 7 | 1.8716321775867E-05 | 7.5780965849172 |
| 61 | 23 | Pantothenate and CoA biosynthesis | 19 | 7 | 8.61623073843592E-05 | 12 | 1.1259388152816E-05 | 7.65248574921987 |
| 62 | 22 | Phagosome | 29 | 16 | 3.39860369101962E-05 | 13 | 4.01004596601329E-06 | 8.47522377504925 |
| 63 | 21 | Pentose and glucuronate interconversions | 10 | 7 | 9.82960924208138E-05 | 3 | 1.13131499736608E-05 | 8.68865812348166 |
| 64 | 20 | Glycerophospholipid metabolism | 32 | 21 | 0.000121941488685 | 11 | 1.33257729688922E-05 | 9.15080040530887 |
| 65 | 19 | RNA transport | 89 | 71 | 3.47716591582055E-05 | 18 | 3.54546918141468E-06 | 9.80735055898335 |
| 66 | 18 | Glutathione metabolism | 32 | 23 | 0.00010155938134 | 9 | 8.47754299851394E-06 | 11.9798131790626 |
| 67 | 17 | ABC transporters | 12 | 7 | 2.56353356554724E-05 | 5 | 1.84415341980058E-06 | 13.9008692987401 |
| 68 | 16 | mRNA surveillance pathway | 42 | 32 | 1.87233218082622E-05 | 10 | 1.25322288901998E-06 | 14.9401371234959 |
| 69 | 15 | Plant hormone signal transduction | 11 | 8 | 1.73100930290953E-05 | 3 | 1.08625225832246E-06 | 15.9356106249463 |
| 70 | 14 | Amino sugar and nucleotide sugar metabolism | 36 | 29 | 0.000143682703211 | 7 | 6.50540254597104E-06 | 22.0866736832287 |
| 71 | 13 | Endocytosis | 53 | 47 | 5.80242121689035E-05 | 6 | 2.60810005145992E-06 | 22.2476941160381 |
| 72 | 12 | Mismatch repair | 17 | 16 | 0.000138524725787 | 1 | 4.91576275271565E-06 | 28.1797012498592 |
| 73 | 11 | Galactose metabolism | 13 | 12 | 0.000114525358523 | 1 | 3.96671931989986E-06 | 28.871555884629 |
| 74 | 10 | **Nucleotide excision repair** | 30 | 28 | 8.52340177432852E-05 | 2 | 2.32634066581331E-06 | 36.6386656072517 |
| 75 | 9 | **Sphingolipid metabolism** | 15 | 13 | 6.57564028834992E-05 | 2 | 1.78192936050591E-06 | 36.9018011268585 |
| 76 | 8 | **Ubiquitin mediated proteolysis** | 54 | 46 | 5.30105246036578E-05 | 8 | 1.17865534727564E-06 | 44.9754245175971 |
| 77 | 7 | **N-Glycan biosynthesis** | 20 | 17 | 0.000103380592831 | 3 | 2.18623598004475E-06 | 47.2870238047137 |
| 78 | 6 | **Base excision repair** | 13 | 12 | 9.49449634465281E-05 | 1 | 8.16144217818975E-07 | 116.333561365238 |
| 79 | 5 | **beta-Alanine metabolism** | 12 | 9 | 0.000168933865656 | 3 | 9.18839776430373E-07 | 183.855629663883 |
| 80 | 4 | **Homologous recombination** | 13 | 12 | 9.3113922773353E-05 | 1 | 2.06205852160388E-07 | 451.558099820215 |
| 81 | 3 | **DNA replication** | 28 | 28 | 0.000299068177796 | 0 | 0 | Inf |
| 82 | 2 | **Proteasome** | 34 | 34 | 7.31794255700302E-05 | 0 | 0 | Inf |
| 83 | 1 | **SNARE interactions in vesicular transport** | 14 | 14 | 3.89955852497964E-05 | 0 | 0 | Inf |

**Table S8. The 100 genes contributing most to the stable state including annotation.**

| identifier | G0 | Gene Name | defline | description |
|---|---|---|---|---|
| | | | **Most stable** | |
| Cre03.g207050.v5.5 | -0.042413480576196 | RPL29 | Ribosomal protein L29, component of cytosolic 80S ribosome and 60S large subunit | Cytosolic 80S ribosomal protein L29; Cytosolic 60S large ribosomal subunit protein L29 |
| Cre10.g420750.v5.5 | -0.041379104310284 | RPL30 | Ribosomal protein L30, component of cytosolic 80S ribosome and 60S large subunit | Cytosolic 80S ribosomal protein L30; Cytosolic 60S large ribosomal subunit protein L30 |
| Cre12.g514500.v5.5 | -0.041144011219975 | RPS11 | Ribosomal protein S11, component of cytosolic 80S ribosome and 40S small subunit | Cytosolic 80S ribosomal protein S11; Cytosolic 40S small ribosomal subunit protein S11 |
| Cre03.g203450.v5.5 | -0.039587294504537 | RPS21 | Ribosomal protein S21, component of cytosolic 80S ribosome and 40S small subunit | Cytosolic 80S ribosomal protein S21; Cytosolic 40S small ribosomal subunit protein S21 |
| Cre16.g682300.v5.5 | -0.039132107680568 | RPS26 | Ribosomal protein S26, component of cytosolic 80S ribosome and 40S small subunit | Cytosolic 80S ribosomal protein S26; Cytosolic 40S small ribosomal subunit protein S26 |
| Cre06.g310700.v5.5 | -0.038952974045301 | RPL36a | Ribosomal protein L36a, component of cytosolic 80S ribosome and 60S large subunit | This gene has also been annotated as RPL41 (according to the nomenclature used for the yeast ribosomal subunits) and shown to be the site of the cycloheximide resistance mutation ACT2. See PMID: 11254126. Cytosolic 80S ribosomal protein L36a; Cytosolic |
| Cre12.g494050.v5.5 | -0.038932448380835 | RPL9 | Ribosomal protein L9, component of cytosolic 80S ribosome and 60S large subunit | Cytosolic 80S ribosomal protein L9; Cytosolic 60S large ribosomal subunit protein L9 |
| Cre12.g529400.v5.5 | -0.038903314666547 | RPS27e1 | Ribosomal protein S27e isoform 1, component of 80S ribosome and 40S small subunit | Cytosolic 80S ribosomal protein S27e, isoform 1; Cytosolic 40S small ribosomal subunit protein S27e, isoform 1. Previously annoated as RPS27-A |
| Cre04.g211800.v5.5 | -0.038865984694867 | RPL23 | Ribosomal protein L23, component of cytosolic 80S ribosome and 60S large subunit | Cytosolic 80S ribosomal protein L23; Cytosolic 60S large ribosomal subunit protein L23 |
| Cre08.g359750.v5.5 | -0.038845210540143 | RPS9 | Ribosomal protein S9, component of cytosolic 80S ribosome and 40S small subunit | Cytosolic 80S ribosomal protein S9; Cytosolic 40S small ribosomal subunit protein S9 |
| Cre12.g498250.v5.5 | -0.038528701001097 | RPS17 | Ribosomal protein S17, component of cytosolic 80S ribosome and 40S small subunit | Cytosolic 80S ribosomal protein S17; Cytosolic 40S small ribosomal subunit protein S17 |
| Cre06.g282500.v5.5 | -0.038515638730758 | RPL23a | Ribosomal protein L23a, component of cytosolic 80S ribosome and 60S large subunit | Cytosolic 80S ribosomal protein L23a; Cytosolic 60S large ribosomal subunit protein L23a |
| Cre07.g325746.v5.5 | -0.038513828597268 | RPL38 | Ribosomal protein L38, component of cytosolic 80S ribosome and 60S large subunit | Cytosolic 80S ribosomal protein L38; Cytosolic 60S large ribosomal subunit protein L38 |
| Cre02.g120150.v5.5 | -0.038503986885013 | RBCS2 | Ribulose-1,5-bisphosphate carboxylase/oxygenase small subunit 2 | RuBisCO small subunit 2, chloroplast precursor [PMID: 3820291] |
| Cre16.g666301.v5.5 | -0.038314522325419 | RPS30 | Ribosomal protein S30, component of cytosolic 80S ribosome and 40S small subunit | Cytosolic 80S ribosomal protein S30; Cytosolic 40S small ribosomal subunit protein S30 |
| Cre02.g143050.v5.5 | -0.038242700621377 | RPP2 | Acidic ribosomal protein P2 | Cytosolic 80S ribosomal protein P2; Cytosolic 60S large ribosomal subunit protein P2 |
| Cre13.g568900.v5.5 | -0.038132207964292 | RPL17 | Ribosomal protein L17, component of cytosolic 80S ribosome and 60S large subunit | Cytosolic 80S ribosomal protein L17; Cytosolic 60S large ribosomal subunit protein L17 |
| Cre08.g382500.v5.5 | -0.037980841499137 | RPS25 | Ribosomal protein S25, component of cytosolic 80S ribosome and 40S small subunit | Cytosolic 80S ribosomal protein S25; Cytosolic 40S small ribosomal subunit protein S25 |
| Cre12.g489153.v5.5 | -0.037976726888621 | #N/A | #N/A | #N/A |
| Cre08.g360900.v5.5 | -0.037617077547882 | RPS15 | Ribosomal protein S15, component of cytosolic 80S ribosome and 40S small subunit | Cytosolic 80S ribosomal protein S15; Cytosolic 40S small ribosomal subunit protein S15 |
| Cre12.g532550.v5.5 | -0.037077567747368 | RPL13a | Ribosomal protein L13a, component of cytosolic 80S ribosome and 60S large subunit | Cytosolic 80S ribosomal protein L13a; Cytosolic 60S large ribosomal subunit protein L13a |
| Cre01.g027000.v5.5 | -0.037001845227908 | RPL11 | Ribosomal protein L11, component of cytosolic 80S ribosome and 60S large subunit | Cytosolic 80S ribosomal protein L11; Cytosolic 60S large ribosomal subunit protein L11 |
| Cre06.g273600.v5.5 | -0.036944474092164 | RPS27a | Ribosomal protein S27a, component of cytosolic 80S ribosome and 40S small subunit | Cytosolic 80S ribosomal protein S27a; Cytosolic 40S small ribosomal subunit protein S27a; ubiquitin region ends with amino acid residue 74. Has homology to ubiquitin superfamily |
| Cre04.g214503.v5.5 | -0.036763936390851 | #N/A | #N/A | #N/A |
| Cre09.g391097.v5.5 | -0.036670114320081 | RPL24 | Ribosomal protein L24, component of cytosolic 80S ribosome and 60S large subunit | Cytosolic 80S ribosomal protein L24; Cytosolic 60S large ribosomal subunit protein L24 |
| Cre10.g456200.v5.5 | -0.03664927693843 | RPS24 | Ribosomal protein S24, component of cytosolic 80S ribosome and 40S small subunit | Cytosolic 80S ribosomal protein S24; Cytosolic 40S small ribosomal subunit protein S24. Possible locus for PR1 (paromomycin resistance) genetic marker |
| Cre14.g617900.v5.5 | -0.036604397300632 | RPL35 | Ribosomal protein L35, component of cytosolic 80S ribosome and 60S large subunit | Cytosolic 80S ribosomal protein L35; Cytosolic 60S large ribosomal subunit protein L35 |
| Cre10.g459250.v5.5 | -0.036601471139178 | RPL35a | Ribosomal protein L35a, component of cytosolic 80S ribosome and 60S large subunit | Cytosolic 80S ribosomal protein L35a; Cytosolic 60S large ribosomal subunit protein L35a |
| Cre10.g430400.v5.5 | -0.036549933605961 | RPL37 | Ribosomal protein L37, component of cytosolic 80S ribosome and 60S large subunit | Cytosolic 80S ribosomal protein L37; Cytosolic 60S large ribosomal subunit protein L37 |
| Cre02.g106600.v5.5 | -0.03646274041787 | RPS19 | Ribosomal protein S19, component of cytosolic 80S ribosome and 40S small subunit | Cytosolic 80S ribosomal protein S19; Cytosolic 40S small ribosomal subunit protein S19 |

| | | | | |
|---|---|---|---|---|
| Cre12.g528750.v5.5 | -0.036435012799071 | RPL12 | Ribosomal protein L12, component of cytosolic 80S ribosome and 60S large subunit | Cytosolic 80S ribosomal protein L12; Cytosolic 60S large ribosomal subunit protein L12 |
| Cre08.g358556.v5.5 | -0.036248750146842 | RPS29 | Ribosomal protein S29, component of cytosolic 80S ribosome and 40S small subunit | Cytosolic 80S ribosomal protein S29; Cytosolic 40S small ribosomal subunit protein S29 |
| Cre06.g272800.v5.5 | -0.035916232912405 | RPS8 | Ribosomal protein S8, component of cytosolic 80S ribosome and 40S small subunit | Cytosolic 80S ribosomal protein S8; Cytosolic 40S small ribosomal subunit protein S8 |
| Cre17.g738300.v5.5 | -0.03584918683167 | RPP1 | Acidic ribosomal protein P1 | Cytosolic 80S ribosomal protein P1; Cytosolic 60S large ribosomal subunit protein P1 |
| Cre16.g660150.v5.5 | -0.035737613566746 | #N/A | #N/A | #N/A |
| Cre02.g102250.v5.5 | -0.035614299533238 | RPS3 | Ribosomal protein S3, component of cytosolic 80S ribosome and 40S small subunit | Cytosolic 80S ribosomal protein S3; Cytosolic 40S small ribosomal subunit protein S3 |
| Cre06.g290950.v5.5 | -0.03559280766374 | RPS5 | Ribosomal protein S5, component of cytosolic 80S ribosome and 40S small subunit | Cytosolic 80S ribosomal protein S5; Cytosolic 40S small ribosomal subunit protein S5 |
| Cre06.g257150.v5.5 | -0.03554370524704 | RPL37a | Ribosomal protein L37a, component of cytosolic 80S ribosome and 60S large subunit | Cytosolic 80S ribosomal protein L37a; Cytosolic 60S large ribosomal subunit protein L37a |
| Cre14.g621450.v5.5 | -0.035398032897396 | RPL5 | Ribosomal protein L5, component of cytosolic 80S ribosome and 60S large subunit | Cytosolic 80S ribosomal protein L5; Cytosolic 60S large ribosomal subunit protein L5 |
| Cre12.g512600.v5.5 | -0.035038175500325 | RPL18 | Ribosomal protein L18, component of cytosolic 80S ribosome and 60S large subunit | Cytosolic 80S ribosomal protein L18; Cytosolic 60S large ribosomal subunit protein L18 |
| Cre02.g115200.v5.5 | -0.034996292191571 | RPL27a | Ribosomal protein L27a, component of cytosolic 80S ribosome and 60S large subunit | Cytosolic 80S ribosomal protein L27a; Cytosolic 60S large ribosomal subunit protein L27a. Candidate gene for the cycloheximide resistance mutation act1. |
| Cre02.g075700.v5.5 | -0.034978863304563 | RPL19 | Ribosomal protein L19, component of cytosolic 80S ribosome and 60S large subunit | Cytosolic 80S ribosomal protein L19; Cytosolic 60S large ribosomal subunit protein L19 |
| Cre01.g007051.v5.5 | -0.034947921258339 | #N/A | #N/A | #N/A |
| Cre07.g331900.v5.5 | -0.034920690387241 | RPS13 | Ribosomal protein S13, component of cytosolic 80S ribosome and 40S small subunit | Cytosolic 80S ribosomal protein S13; Cytosolic 40S small ribosomal subunit protein S13 |
| Cre17.g701650.v5.5 | -0.034874742420666 | RPL27 | Ribosomal protein L27, component of cytosolic 80S ribosome and 60S large subunit | Cytosolic 80S ribosomal protein L27; Cytosolic 60S large ribosomal subunit protein L27 |
| Cre09.g388200.v5.5 | -0.034794257456671 | RPL10 | Ribosomal protein L10, component of cytosolic 80S ribosome and 60S large subunit | Cytosolic 80S ribosomal protein L10; Cytosolic 60S large ribosomal subunit protein L10 |
| Cre12.g498900.v5.5 | -0.034719764220557 | RPS7 | Ribosomal protein S7, component of cytosolic 80S ribosome and 40S small subunit | Cytosolic 80S ribosomal protein S7; Cytosolic 40S small ribosomal subunit protein S7 |
| Cre02.g091100.v5.5 | -0.034719444120971 | RPL15 | Ribosomal protein L15, component of cytosolic 80S ribosome and 60S large subunit | Cytosolic 80S ribosomal protein L15; Cytosolic 60S large subunit ribosomal protein L15 |
| Cre10.g417700.v5.5 | -0.034652304538003 | RPL3 | Ribosomal protein L3, component of cytosolic 80S ribosome and 60S large subunit | Cytosolic 80S ribosomal protein L3; Cytosolic 60S large ribosomal subunit protein L3 |
| Cre09.g405106.v5.5 | -0.034634630358681 | #N/A | #N/A | #N/A |
| Cre12.g537800.v5.5 | -0.03458260358644 | RPL7 | Ribosomal protein L7, component of cytosolic 80S ribosome and 60S large subunit | Cytosolic 80S ribosomal protein L7; Cytosolic 60S large ribosomal subunit protein L7 |
| Cre12.g504200.v5.5 | -0.034550459414828 | RPS23 | Ribosomal protein S23, component of cytosolic 80S ribosome and 40S small subunit | Cytosolic 80S ribosomal protein S23; Cytosolic 40S small ribosomal subunit protein S23 |
| Cre06.g278135.v5.5 | -0.034524745728077 | RPL21 | Ribosomal protein L21, component of cytosolic 80S ribosome and 60S large subunit | Cytosolic 80S ribosomal protein L21; Cytosolic 60S large ribosomal subunit protein L21 |
| Cre06.g278222.v5.5 | -0.034360678909814 | RCK1 | Receptor of activated protein kinase C | Receptor of activated protein kinase C 1, component of 40S small ribosomal subunit; Also cytosolic 40S small ribosomal subunit protein RACK1. Previously annotated as RACK1 and CBLP. Initially described [PMID: 2116589] as CBLP. Smith and Lee 2008 [PMID: |
| Cre16.g661050.v5.5 | -0.033918227742958 | RPL34 | Ribosomal protein, L34e superfamily, component of cytosolic 80S ribosome and 60S large subunit | Cytosolic 80S ribosomal protein L34; Cytosolic 60S large ribosomal subunit protein L34. Belongs to Ribosomal_L34e superfamily |
| Cre09.g402219.v5.5 | -0.033849999760451 | LCI3 | Low-CO2-inducible protein | Regulated by CCM1 [PMID: 15235119]. Acclimation to changing CO2 concentrations and light intensities was studied by Yamano et al. 2008 [PMID: 18322145]. |
| Cre12.g529651.v5.5 | -0.033764842424319 | #N/A | #N/A | #N/A |
| Cre06.g272950.v5.5 | -0.03370671310797 | RPS18 | Ribosomal protein S18, component of cytosolic 80S ribosome and 40S small subunit | Cytosolic 80S ribosomal protein S18; Cytosolic 40S small ribosomal subunit protein S18 |
| Cre12.g520500.v5.5 | -0.03352743568126 | RPP0 | Acidic ribosomal protein P0, Ribosomal protein L10 | Cytosolic 80S acidic ribosomal protein P0; Cytosolic 60S large ribosomal subunit protein P0. Ribosomal protein L10 |
| Cre13.g568650.v5.5 | -0.033525675237866 | RPS3a | Ribosomal protein S3a, component of cytosolic 80S ribosome and 40S small subunit | Cytosolic 80S ribosomal protein S3a; Cytosolic 40S small ribosomal subunit protein S3a |
| Cre07.g357850.v5.5 | -0.033435738345439 | RPL22 | Ribosomal protein L22, component of cytosolic 80S ribosome and 60S large subunit | Cytosolic 80S ribosomal protein L22; Cytosolic 60S large ribosomal subunit protein L22 |
| Cre06.g249250.v5.5 | -0.033424997844404 | RPL7ae | Ribosomal protein L7Ae | Cytosolic 80S ribosomal protein L7ae; Cytosolic 60S large ribosomal subunit protein L7ae |
| Cre06.g261000.v5.5 | -0.033370970149946 | PSBR | 10 kDa photosystem II polypeptide | Similar to At1g79040, chloroplast-targeted |
| Cre11.g467578.v5.5 | -0.03331154318676 | #N/A | #N/A | #N/A |

| | | | | |
|---|---|---|---|---|
| Cre01.g040000.v5.5 | -0.033138221316172 | RPL26 | Ribosomal protein L26, component of cytosolic 80S ribosome and 60S large subunit | Cytosolic 80S ribosomal protein L26; Cytosolic 60S large ribosomal subunit protein L26 |
| Cre17.g701200.v5.5 | -0.033058019581648 | RPL14 | Ribosomal protein L14, component of cytosolic 80S ribosome and 60S large subunit | Cytosolic 80S ribosomal protein L14; Cytosolic 60S large ribosomal subunit protein L14 |
| Cre01.g047750.v5.5 | -0.033045067712998 | RPL18a | Ribosomal protein L18a, component of cytosolic 80S ribosome and 60S large subunit | Cytosolic 80S ribosomal protein L18a; Cytosolic 60S large ribosomal subunit protein L18a |
| Cre06.g308250.v5.5 | -0.033024373977157 | RPS4 | Ribosomal protein S4, component of cytosolic 80S ribosome and 40S small subunit | Cytosolic 80S ribosomal protein S4; Cytosolic 40S small ribosomal subunit protein S4 |
| Cre12.g484050.v5.5 | -0.032758780612405 | RPL36 | Ribosomal protein L36, component of cytosolic 80S ribosome and 60S large subunit | Cytosolic 80S ribosomal protein L36; Cytosolic 60S large ribosomal subunit protein L36 |
| Cre03.g182551.v5.5 | -0.03250281882078 | PCY1 | Pre-apoplastocyanin | Pre-apoplastocyanin copper binding protein, PETE [PMID: 2165059; PMID: 8940133]; structure of plastocyanin PDB: 2PLT; mutant = ac208 [PMID: 8463310] |
| Cre01.g039250.v5.5 | -0.032452807781255 | RPS2 | Ribosomal protein S2, component of cytosolic 80S ribosome and 40S small subunit | Cytosolic 80S ribosomal protein S2; Cytosolic 40S small ribosomal subunit protein S2 |
| Cre14.g626700.v5.5 | -0.032402639703491 | PETF | Ferredoxin | 2Fe-2S containing redox protein involved in photosynthetic electron transfer, chloroplast localization [PMID: 16656453] |
| Cre09.g400650.v5.5 | -0.032209166822848 | RPS6 | Ribosomal protein S6, component of cytosolic 80S ribosome and 40S small subunit | Cytosolic 80S ribosomal protein S6; Cytosolic 40S small ribosomal subunit protein S6 |
| Cre02.g101350.v5.5 | -0.032187682328782 | RPL10a | Ribosomal protein L10a, component of cytosolic 80S ribosome and 60S large subunit | Cytosolic 80S ribosomal protein L10a; Cytosolic 60S large ribosomal subunit protein L10a |
| Cre09.g411100.v5.5 | -0.031850354389245 | RPS10 | Ribosomal protein S10, component of cytosolic 80S ribosome and 40S small subunit | Cytosolic 80S ribosomal protein S10; Cytosolic 40S small ribosomal subunit protein S10 |
| Cre12.g498600.v5.5 | -0.031848443989171 | #N/A | Eukaryotic translation elongation factor 1 alpha | Flagellar Associated Protein, found in the flagellar proteome [PMID: 15998802]. Previously annotated as just EEF1 |
| Cre12.g535851.v5.5 | -0.031698709415264 | #N/A | #N/A | #N/A |
| Cre10.g432800.v5.5 | -0.03140255910603 | RPSa | Ribosomal protein Sa, component of cytosolic 80S ribosome and 40S small subunit | Cytosolic 80S ribosomal protein Sa; Cytosolic 40S small ribosomal subunit protein Sa |
| Cre06.g289550.v5.5 | -0.031357587761394 | RPL32 | Ribosomal protein L32, component of cytosolic 80S ribosome and 60S large subunit | Cytosolic 80S ribosomal protein L32; Cytosolic 60S large ribosomal subunit protein L32 |
| Cre01.g011000.v5.5 | -0.0313480234275 | RPL6 | Ribosomal protein L6, component of cytosolic 80S ribosome and 60S large subunit | Cytosolic 80S ribosomal protein L6; Cytosolic 60S large ribosomal subunit protein L6 |
| Cre01.g066917.v5.5 | -0.031269557867695 | LHCBM1 | Chlorophyll a/b binding protein of LHCII | Chlorophyll a-b binding protein of LHCII |
| Cre03.g190950.v5.5 | -0.031200648389157 | TUA1 | Alpha tubulin 1 | Alpha tubulin 1 |
| Cre10.g452350.v5.5 | -0.031153722520479 | #N/A | #N/A | #N/A |
| Cre11.g480150.v5.5 | -0.031026695131996 | RPS14 | Ribosomal protein S14, component of cytosolic 80S ribosome and 40S small subunit | Cytosolic 80S ribosomal protein S14; Cytosolic 40S small ribosomal subunit protein S14 |
| Cre05.g234637.v5.5 | -0.03096127351989 | #N/A | #N/A | #N/A |
| Cre08.g372200.v5.5 | -0.030773403723922 | #N/A | #N/A | #N/A |
| Cre05.g234550.v5.5 | -0.03067184285381 | FBA3 | Fructose-1,6-bisphosphate aldolase | Fructose-1,6-bisphosphate aldolase 1 (EC:4.1.2.13); probably plastidic, but predicted mitochondrial by Target-P; 87% identical to cDNA (Q42690); glycolytic enzyme that catalyses the reversible aldol cleavage or condensation of fructose-1,6-bisphosphate in |
| Cre06.g263450.v5.5 | -0.030619375917674 | #N/A | #N/A | #N/A |
| Cre16.g659700.v5.5 | -0.030458018527192 | #N/A | #N/A | #N/A |
| Cre13.g580000.v5.5 | -0.030163619982343 | #N/A | #N/A | #N/A |
| Cre07.g337100.v5.5 | -0.030146050506178 | #N/A | #N/A | #N/A |
| Cre10.g420350.v5.5 | -0.030119630592311 | PSAE | Photosystem I 8.1 kDa reaction center subunit IV | Photosystem I reaction center subunit IV, chloroplast precursor (PSI-E) (Photosystem I 8.1 kDa protein) [PMID: 2693938] |
| Cre07.g323800.v5.5 | -0.029959977080676 | #N/A | #N/A | #N/A |
| Cre06.g282800.v5.5 | -0.029680494422761 | ICL1 | Isocitrate lyase | Isocitrate lyase (EC 4.1.3.1); isocitrase; 98% identical to cDNA (AAB61446) [PMID: 9049260]; PMID: 19847013; Listed by Rolland et al. 2008 [PMID: 19451016] in a table of putative redox-regulated proteins identified by proteomic methods |
| Cre17.g724300.v5.5 | -0.029565205566247 | PSAK | Photosystem I reaction center subunit psaK | 8.4 kD subunit of photosystem I (polypeptide 37) [PMID: 2693938] |
| Cre13.g579450.v5.5 | -0.029524400126888 | CST1 | Chlamy-specific membrane transporter of unknown function | Transmembrane protein; shows similarity to various transporters and permeases; highly expressed; small gene family appears specific to Chlamydomonas |
| Cre06.g298650.v5.5 | -0.029339168655577 | #N/A | #N/A | #N/A |
| Cre13.g573351.v5.5 | -0.029311290088974 | #N/A | #N/A | #N/A |
| Cre09.g397697.v5.5 | -0.029287916533883 | RPL4 | Ribosomal protein L4, component of cytosolic 80S ribosome and 60S large subunit | Cytosolic 80S ribosomal protein L4; Cytosolic 60S large ribosomal subunit protein L4 |
| Cre12.g546150.v5.5 | -0.029236218915832 | PETM | Cytochrome b6f complex PetM subunit | Cytochrome b6f complex chain PetM, chloroplast precursor; GI:2493687; PMID: 8631873, PMID: 8616155, PMID: 7493968 |

**Table S9. The 100 genes contributing the most to the exponential state including annotation.**

**100 genes contributing most to the exponential state**

| identifier | G1 | Gene Name | defline | description |
|---|---|---|---|---|
| Cre06.g284250.v5.5 | 0.055996613203082 | LHCBM8 | Chlorophyll a/b binding protein of LHCII | Chlorophyll a-b binding protein of LHCII |
| Cre06.g278213.v5.5 | 0.050465962662738 | LHCA6 | Light-harvesting protein of photosystem I | #N/A |
| Cre16.g675550.v5.5 | 0.050058582384222 | #N/A | Peptidyl-prolyl cis-trans isomerase, FKBP-type | FKBP-type peptidyl-prolyl cis-trans isomerase (EC 5.2.1.8) (PPiase) (Rotamase), orthologous to At5g45680; isoform a of gene FKB16-2, probably directed to thylakoid lumen (TP length 26, RR motif); other splicing isoforms possibly directed to stroma or mito |
| Cre12.g546550.v5.5 | 0.049154804201197 | FEA1 | Fe-assimilating protein | High-CO2 inducible, iron-deficiency inducible, periplasmic protein. Changes in expression with light intensity were studied by Long and Merchant 2008 [PMID: 19067961]. Cis-acting regulatory elements were characterized by Fei et al. 2009 [PMID: 19351705]. |
| Cre06.g257601.v5.5 | 0.048187442158047 | PRX1 | 2-cys peroxiredoxin, chloroplastic | 2-cys Peroxiredoxin, thioredoxin dependent peroxidase, chloroplast precursor, PMID: 11784321. Protein processed at aa 39 to produce N-terminus of SHAEKPL as determined by Edman degradation |
| Cre07.g334550.v5.5 | 0.04807240094068 | PSAO | Photosystem I subunit O | Photosystem I subunit O; similar to 10-kDa subunit of eukaryotic photosystem I [PMID: 11801243]; microarray element: 894019E07 |
| Cre17.g700950.v5.5 | 0.047246862010625 | FDX5 | Apoferredoxin | Fe2S2 containing redox protein, predicted chloroplast localization |
| Cre03.g207800.v5.5 | 0.044642888826623 | #N/A | #N/A | #N/A |
| Cre12.g548950.v5.5 | 0.044459325609396 | LHCBM7 | Chlorophyll a/b binding protein of LHCII | Chlorophyll a-b binding protein of LHCII |
| Cre12.g516700.v5.5 | 0.044074477466568 | #N/A | #N/A | #N/A |
| Cre02.g082750.v5.5 | 0.04400684293239 | PSBX | 4.1 kDa photosystem II subunit | Encoding a 4.1 kDa subunit for PS2 with a single TMH [PMID: 16143838]. |
| Cre12.g559800.v5.5 | 0.04378386752357 | #N/A | #N/A | #N/A |
| Cre06.g283950.v5.5 | 0.043524311561079 | LHCBM4 | Chlorophyll a/b binding protein of LHCII | PSII-associated chlorophyll a/b-binding protein. Identified in the eyespot phosphoproteome by Wagner et al. 2008 [PMID: 18065559] |
| Cre02.g145050.v5.5 | 0.043109825830372 | CMK1 | 4-diphosphocytidyl-2-C-methyl-D-erythritol kinase, chloroplast precursor | 4-diphosphocytidyl-2-C-methyl-D-erythritol kinase (4-(cytidine-5'-diphospho)-2-C-methyl-D-erythritol kinase) (CDPMEK) (ispE) (ychB), chloroplast precursor |
| Cre09.g407600.v5.5 | 0.041749395967091 | #N/A | #N/A | #N/A |
| Cre12.g548955.v5.5 | 0.041409361376581 | #N/A | #N/A | #N/A |
| Cre12.g516717.v5.5 | 0.040259081203751 | #N/A | #N/A | #N/A |
| Cre02.g101700.v5.5 | 0.040116763680551 | #N/A | #N/A | #N/A |
| Cre01.g013100.v5.5 | 0.040086588029322 | PRL10 | Predicted extracellular protein | Similar to pathogenesis-related protein 1, extracellular |
| Cre04.g226600.v5.5 | 0.039958289511076 | #N/A | #N/A | #N/A |
| Cre06.g268600.v5.5 | 0.039908483445275 | NAB1 | Nucleic acid binding protein | Nucleic acid binding protein; RNA binding protein involved in the light-regulated differential expression of the light-harvesting antenna of Chlamydomonas reinhardtii [PMID: 16284312, Genbank entry AY157846]. Studied by Wobbe et al. 2009 [PMID: 19666611] |
| Cre06.g280750.v5.5 | 0.039745072457031 | #N/A | #N/A | #N/A |
| Cre12.g497300.v5.5 | 0.039533890589178 | #N/A | Rhodanese-like Ca-sensing receptor | Rhodanese-like, Ca-sensing receptor, chloroplast location proposed; present in thylakoid-enriched fraction based on mass spectrometric peptide identification [PMID: 17078018]. Busch et al. (2010) (Chlamy 2010 Meeting abstract) proposed that this protein |
| Cre04.g232104.v5.5 | 0.038910075857458 | LHCBM3 | Light-harvesting complex II chlorophyll a/b binding protein M3 | #N/A |
| Cre02.g089000.v5.5 | 0.038755308995787 | #N/A | #N/A | #N/A |
| Cre17.g708750.v5.5 | 0.038713873474805 | #N/A | #N/A | #N/A |
| Cre08.g370401.v5.5 | 0.038576546257935 | #N/A | #N/A | #N/A |
| Cre06.g302950.v5.5 | 0.03837884156655 | #N/A | #N/A | #N/A |
| Cre07.g330250.v5.5 | 0.038295956000591 | PSAH | Subunit H of photosystem I | Formerly called protein P28. See PMID: 2693938. |
| Cre12.g503900.v5.5 | 0.038050905747892 | #N/A | #N/A | #N/A |
| Cre02.g076300.v5.5 | 0.038002231932874 | UPD2 | Uroporphyrinogen decarboxylase | Uroporphyrinogen decarboxylase, chloroplast precursor (URO-D) (UPD) (HemE). Previously annotated as UROD2 |
| Cre06.g300700.v5.5 | 0.037865179312019 | #N/A | #N/A | #N/A |
| Cre02.g089650.v5.5 | 0.037860567143789 | #N/A | #N/A | #N/A |
| Cre01.g011630.v5.5 | 0.037668718712836 | #N/A | #N/A | #N/A |
| Cre03.g158000.v5.5 | 0.037441522612527 | GSA1 | Glutamate-1-semialdehyde aminotransferase | Glutamate-1-semialdehyde aminotransferase (GSA-AT); Glutamate-1-semialdehyde 2,1-aminomutase, chloroplast precursor; PMID: 16126849; PMID: 15890644; PMID: 12228605; PMID: 10872234; PMID: 10080695; PMID: 8989881; PMID: 8155881; PMID: 1985889; predicted chl |
| Cre13.g602650.v5.5 | 0.037406078310922 | #N/A | #N/A | #N/A |
| Cre14.g626750.v5.5 | 0.037381936460096 | #N/A | #N/A | #N/A |
| Cre05.g240300.v5.5 | 0.037210154464508 | #N/A | #N/A | #N/A |
| Cre17.g720350.v5.5 | 0.037153349383248 | MINE1 | Chloroplast division site-determinant MinE | Helps define position of the chloroplast division ring (PMID: 11743109); conserved in plants and eubacteria. Characterized by Adams et al. 2008 [PMID: 18270733]. |

| | | | | |
|---|---|---|---|---|
| Cre12.g519850.v5.5 | 0.037095363639312 | #N/A | #N/A | #N/A |
| Cre12.g551950.v5.5 | 0.037085722779803 | CGL38 | Predicted protein | Conserved expressed protein of unknown function; similar to protein of unknown function in Arabidopsis and rice. cd00204. Contains 4 consecutive ankyrin repeats |
| Cre01.g004700.v5.5 | 0.036847423640864 | #N/A | #N/A | #N/A |
| Cre10.g442750.v5.5 | 0.036835435315033 | #N/A | #N/A | #N/A |
| Cre02.g143450.v5.5 | 0.036745385184473 | #N/A | #N/A | #N/A |
| Cre06.g266550.v5.5 | 0.036581419186997 | #N/A | #N/A | #N/A |
| Cre07.g334800.v5.5 | 0.036465372420429 | FDX4 | Ferredoxin | Fe2S2 containing redox protein, predicted chloroplast localization |
| Cre07.g313143.v5.5 | 0.036365423967029 | #N/A | #N/A | #N/A |
| Cre02.g080250.v5.5 | 0.036283300772534 | #N/A | #N/A | #N/A |
| Cre09.g412400.v5.5 | 0.036218450020991 | #N/A | #N/A | #N/A |
| Cre16.g673650.v5.5 | 0.036190978660225 | LHCB5 | Minor chlorophyll a/b binding protein of photosystem II | Minor chlorophyll a-b binding protein of photosystem II. Included among genes whose expression levels changed significantly during hydrogen production in sulfur-deficient cells; Nguyen et al. 2008 [PMID: 18708561]. Protein product also known as CP26 |
| Cre17.g696350.v5.5 | 0.036080622900095 | #N/A | #N/A | #N/A |
| Cre09.g397623.v5.5 | 0.036007137767625 | #N/A | #N/A | #N/A |
| Cre12.g522950.v5.5 | 0.03582923308604 | MIND1 | Chloroplast septum site-determining protein | Putative chloroplast septum site-determining protein MinD. Chloroplast-encoded in other Chlorophyte algae. Characterized by Adams et al. 2008 [PMID: 18270733]. |
| Cre03.g170550.v5.5 | 0.035814790741702 | #N/A | #N/A | #N/A |
| Cre02.g078507.v5.5 | 0.035802445018855 | #N/A | #N/A | #N/A |
| Cre06.g302500.v5.5 | 0.035777715077089 | #N/A | #N/A | #N/A |
| Cre13.g602450.v5.5 | 0.03575673401548 | #N/A | #N/A | #N/A |
| Cre16.g674300.v5.5 | 0.035720655932985 | FAP269 | Flagellar Associated Protein | Conserved uncharacterized Flagellar Associated Protein with ankyrin repeats; found in the flagellar proteome [PMID: 15998802] |
| Cre02.g085450.v5.5 | 0.035697346180366 | CPX1 | Coproporphyrinogen III oxidase | Coproporphyrinogen III oxidase, chloroplast precursor; GI:837299; CPO1; HEM13; HEM6 [PMID: 15849308]; Predicted chloroplast transit peptide 1-31 |
| Cre09.g414000.v5.5 | 0.035672135809172 | #N/A | #N/A | #N/A |
| Cre07.g325736.v5.5 | 0.035563644694878 | #N/A | #N/A | #N/A |
| Cre08.g372550.v5.5 | 0.035563502305416 | CDKB1 | Plant specific cyclin dependent kinase | Plant specific cyclin dependent kinase; CDKB subfamily; PSTTLRE motif; mitotic regulator. Putative function in G2/M |
| Cre14.g625000.v5.5 | 0.035147448461403 | #N/A | #N/A | #N/A |
| Cre01.g049000.v5.5 | 0.035032939569305 | #N/A | Pterin dehydratase | 4a-hydroxytetrahydrobiopterin dehydratase. Green-lineage conserved expressed protein. Previously annotated as CGL31 |
| Cre06.g259450.v5.5 | 0.03480485696617 | #N/A | #N/A | #N/A |
| Cre16.g678650.v5.5 | 0.034741292189895 | #N/A | #N/A | #N/A |
| Cre03.g199450.v5.5 | 0.034718282134375 | MINE2 | Chloroplast division site determinant | Putative MinE homolog, chloroplast division site-determinant |
| Cre09.g393173.v5.5 | 0.034631473744295 | ELI2 | Early light-inducible protein | A homolog of ELIP (early light-inducible protein) gene which was originally identified as transiently expressed during the early phase of greening in etiolated pea seedlings [PMID: 6692824]. It has three TMH, whose sequences are closely related to LHC pro |
| Cre02.g086100.v5.5 | 0.034619268349099 | CGL40 | Predicted protein | Conserved expressed protein of unidentified function; some similarity to Arabidopsis protein gi\|18402865\|ref\|NP_564561.1\| - Also a domain COG0741: Soluble lytic murein transglycosylase and related regulatory proteins in Rickettsia |
| Cre12.g501250.v5.5 | 0.034498413144327 | #N/A | #N/A | #N/A |
| Cre05.g237150.v5.5 | 0.034492306250579 | #N/A | #N/A | #N/A |
| Cre03.g201500.v5.5 | 0.034452458193624 | #N/A | #N/A | #N/A |
| Cre12.g523900.v5.5 | 0.034444764717794 | #N/A | #N/A | #N/A |
| Cre16.g693200.v5.5 | 0.034355236761613 | #N/A | #N/A | #N/A |
| Cre14.g627850.v5.5 | 0.034266018343334 | #N/A | #N/A | #N/A |
| Cre03.g179820.v5.5 | 0.034216235085678 | #N/A | #N/A | #N/A |
| Cre07.g354976.v5.5 | 0.034111588271749 | #N/A | #N/A | #N/A |
| Cre05.g238600.v5.5 | 0.034110524484057 | #N/A | #N/A | #N/A |
| Cre12.g520050.v5.5 | 0.034092236484209 | #N/A | #N/A | #N/A |
| Cre12.g497850.v5.5 | 0.034085974668822 | #N/A | #N/A | #N/A |
| Cre09.g403071.v5.5 | 0.034084338406364 | #N/A | #N/A | #N/A |
| Cre16.g688800.v5.5 | 0.034052616582133 | #N/A | #N/A | #N/A |
| Cre10.g441250.v5.5 | 0.033988880568631 | #N/A | #N/A | #N/A |
| Cre01.g003850.v5.5 | 0.03397038688887 | #N/A | Cytochrome P450, CYP197 superfamily | Cytochrome P450, CYP197 superfamily, CYP197B family. Previously annotated as CYP747A1 |
| Cre14.g619450.v5.5 | 0.033969577948411 | #N/A | #N/A | #N/A |
| Cre17.g732250.v5.5 | 0.033933393338647 | #N/A | #N/A | #N/A |

| | | | | | |
|---|---|---|---|---|---|
| Cre09.g386167.v5.5 | 0.033773497208234 | #N/A | #N/A | | #N/A |
| Cre17.g726400.v5.5 | 0.033763914731162 | #N/A | #N/A | | #N/A |
| Cre11.g482700.v5.5 | 0.033612210891776 | #N/A | #N/A | | #N/A |
| Cre06.g278104.v5.5 | 0.033607118426563 | #N/A | #N/A | | #N/A |
| Cre03.g181550.v5.5 | 0.033601138820279 | #N/A | #N/A | | #N/A |
| Cre10.g439250.v5.5 | 0.033499830723557 | #N/A | #N/A | | #N/A |
| Cre01.g066917.v5.5 | 0.033474616717733 | LHCBM1 | Chlorophyll a/b binding protein of LHCII | Chlorophyll a-b binding protein of LHCII | |
| Cre06.g278204.v5.5 | 0.033427881822105 | #N/A | #N/A | | #N/A |
| Cre03.g156050.v5.5 | 0.033353830595088 | #N/A | #N/A | | #N/A |
| Cre16.g667850.v5.5 | 0.033303898546312 | #N/A | #N/A | | #N/A |
| Cre06.g273450.v5.5 | 0.03327151925858 | #N/A | #N/A | | #N/A |
| Cre11.g467672.v5.5 | 0.033159917703381 | #N/A | #N/A | | #N/A |
| Cre10.g436500.v5.5 | 0.033098193533785 | #N/A | #N/A | | #N/A |
| Cre09.g386155.v5.5 | 0.033023182733131 | #N/A | #N/A | | #N/A |

## Table S10. The 100 genes contributing the most to the stationary state including annotation.

**100 genes contributing most to the stationary state**

| identifier | G0 | Gene Name | defline | description |
|---|---|---|---|---|
| Cre02.g144650.v5.5 | -0.039092277585641 | PTB12 | Sodium/phosphate symporter | Putative phosphate transporter, similar to yeast Pho89 and Neurospora PHO4 probable Na+/Pi symporters; present in a cluster of four highly related PTB family members on scaffold 24, including PTB9, PTB5 and PTB4. |
| Cre24.g755547.v5.5 | -0.037814401767473 | #N/A | #N/A | #N/A |
| Cre17.g713700.v5.5 | -0.035629644054225 | #N/A | #N/A | #N/A |
| Cre02.g085500.v5.5 | -0.032200883433109 | #N/A | #N/A | #N/A |
| Cre08.g379800.v5.5 | -0.030624807628227 | #N/A | #N/A | #N/A |
| Cre01.g030350.v5.5 | -0.028948703123221 | CGL41 | Predicted protein | Conserved expressed protein of unknown function |
| Cre12.g495952.v5.5 | -0.027695403816197 | #N/A | #N/A | #N/A |
| Cre03.g163950.v5.5 | -0.027496470120439 | CDO2 | Cysteine dioxygenase | Cysteine dioxygenase |
| Cre07.g328800.v5.5 | -0.027066349440318 | NSG13 | Protein expressed during nitrogen-starved gametogenesis | Predicted to contain 3 transmembrane helices and 4 Armadillo repeats; expressed exclusively during gametogenesis, at a late stage (PMID: 15459796). Identified by Abe et al. 2004 [PMID: 15459796] as NSG13, a gene expressed during nitrogen-starved gametoge |
| Cre12.g518800.v5.5 | -0.026556562718769 | #N/A | #N/A | #N/A |
| Cre14.g630350.v5.5 | -0.026442135732279 | #N/A | #N/A | #N/A |
| Cre16.g690319.v5.5 | -0.026043091119337 | #N/A | #N/A | #N/A |
| Cre08.g380800.v5.5 | -0.026009818158272 | #N/A | #N/A | #N/A |
| Cre11.g468800.v5.5 | -0.025713632066336 | #N/A | #N/A | #N/A |
| Cre12.g545101.v5.5 | -0.025358074085579 | #N/A | #N/A | #N/A |
| Cre06.g279450.v5.5 | -0.024469338365996 | MUT68 | Polyadenylate polymerase beta nucleotidyltransferase | Noncanonical polyadenylate polymerase that adds untemplated adenines to the 5' RNA fragments after siRNA-mediated cleavage and appears to stimulate their exosome-dependent degradation. Discussed by Zimmer et al. 2008 [PMID: 18493045]. The role of MUT68 i |
| Cre02.g096250.v5.5 | -0.023618451139756 | FMO9 | Flavin-containing monooxygenase | Flavin-containing monooxygenase. This family includes diverse enzymes that utilise FAD - 2-polyprenyl-6-methoxyphenol hydroxylase and related FAD-dependent oxidoreductases [Coenzyme metabolism/Energy production and conversion - this gene is about 20 kb aw |
| Cre02.g144700.v5.5 | -0.023536524058637 | PTB5 | Sodium/phosphate symporter | Putative phosphate transporter, similar to yeast Pho89 and Neurospora PHO4 probable Na+/Pi symporters; present in a cluster of four highly related PTB family members on scaffold 24, including PTB9, PTB12 and PTB4. |
| Cre03.g204465.v5.5 | -0.022973589795245 | #N/A | #N/A | #N/A |
| Cre09.g400750.v5.5 | -0.022956997799386 | AMT5 | Ammonium transporter | Belongs to AMT1 family (Amt1;5 or Amt1.5) two different splicing variants have been found for this gene, this one is AMT5b, the shorter one with additional intron (AY588243); was AMT1Eb; belongs to AMT1 family (Amt1;5 or Amt1.5); two different splicing va |
| Cre02.g095500.v5.5 | -0.022608409238979 | #N/A | #N/A | #N/A |
| Cre12.g488450.v5.5 | -0.022415726479985 | VSP3 | Extracellular matrix protein | Extracellular matrix protein (vegetative cell wall protein) similar to ISG (Volvox carteri) [PMID: 1600938], both are glycoproteins with a hydroxyproline-rich (HR) domain. [PMID: 8000007. ISG Genbank entry X65165; VSP3, L29029; C. incerta ortholog, AY7950 |
| Cre12.g531000.v5.5 | -0.022344377616321 | AMT8 | Ammonium transporter | Belongs to AMT1 family (Amt1;8); corresponds to AY548754, mRNA complete cds (PMID: 15821986). Previously annotated as AMT1H |
| Cre06.g284150.v5.5 | -0.022310191578858 | RHP2 | Rh protein | Similar to Rh50 gene product of Drosophila melanogaster |
| Cre17.g707750.v5.5 | -0.02208774284382 | #N/A | #N/A | #N/A |
| Cre03.g145787.v5.5 | -0.021966383347445 | HSP22C | Heat shock protein 22C | Small heat shock protein containing an alpha-crystallin domain; N-terminus by TargetP weakly predicted to be chloroplast transit peptide; PMID: 16143837 |
| Cre06.g271900.v5.5 | -0.02183714786302 | #N/A | #N/A | #N/A |
| Cre17.g701000.v5.5 | -0.021691986731389 | #N/A | #N/A | #N/A |
| Cre15.g636950.v5.5 | -0.021589869279906 | #N/A | #N/A | #N/A |
| Cre12.g528600.v5.5 | -0.020953907848077 | #N/A | #N/A | #N/A |
| Cre01.g053300.v5.5 | -0.020862794824919 | AIH1 | Agmatine iminohydrolase | Converts agmatin to N-carbamoyl-putrescine; involved in agmatine pathway of polyamine (putrescine) biosynthesis; could be organelle-targeted |
| Cre08.g380700.v5.5 | -0.020424054650379 | #N/A | #N/A | #N/A |
| Cre09.g413350.v5.5 | -0.020422473627258 | #N/A | #N/A | #N/A |
| Cre06.g260700.v5.5 | -0.020335186921844 | XUV1 | Xanthine/uracil/vitamin C permease-like | Xanthine/uracil/vitamin C permease-like. Related to plants and fungi; also related to bacterial inner membrane proteins |
| Cre12.g541500.v5.5 | -0.020089454421164 | #N/A | #N/A | #N/A |
| Cre10.g448400.v5.5 | -0.020034684258556 | #N/A | #N/A | #N/A |
| Cre12.g551700.v5.5 | -0.01985844789748 | #N/A | #N/A | #N/A |
| Cre10.g424750.v5.5 | -0.019811343425543 | PPD1 | Pyruvate phosphate dikinase | Pyruvate phosphate dikinase (PPDK) (EC 2.7.9.1); Pyruvate orthophosphate dikinase (PODK); Target-P predicts no organelle |

| | | | | | |
|---|---|---|---|---|---|
| | | | | | targeting sequence; similar to PPDK from Phytophthora (GenBank AAK74150) |
| Cre10.g429500.v5.5 | -0.019784618414886 | #N/A | | #N/A | #N/A |
| Cre05.g243358.v5.5 | -0.019602737549601 | #N/A | | #N/A | #N/A |
| Cre10.g427896.v5.5 | -0.019422697764296 | #N/A | | #N/A | #N/A |
| Cre12.g488050.v5.5 | -0.019382291007925 | #N/A | | #N/A | #N/A |
| Cre10.g426152.v5.5 | -0.019286634853558 | #N/A | | #N/A | #N/A |
| Cre03.g152600.v5.5 | -0.019274381175217 | #N/A | | #N/A | #N/A |
| Cre06.g297049.v5.5 | -0.019020794649341 | #N/A | | #N/A | #N/A |
| Cre06.g285950.v5.5 | -0.019018567393163 | #N/A | | #N/A | #N/A |
| Cre03.g202785.v5.5 | -0.019009776425972 | #N/A | | #N/A | #N/A |
| Cre08.g368300.v5.5 | -0.018914752574756 | #N/A | | #N/A | #N/A |
| Cre08.g360550.v5.5 | -0.018893806271714 | ERM3 | ERD4-related membrane protein | | NO-forming, copper-containing nitrite reductase. N-terminus: transmembrane protein with a DUF221 domain of unknown function; this domain is present in many eukaryotes, in particular in Arabidopsis ERD4 (early response to dehydration); belongs to a cluster |
| Cre17.g714300.v5.5 | -0.018768629837689 | #N/A | | #N/A | #N/A |
| Cre16.g694206.v5.5 | -0.018723517437475 | #N/A | | #N/A | #N/A |
| Cre09.g395213.v5.5 | -0.01869721389458 | #N/A | | #N/A | #N/A |
| Cre02.g073600.v5.5 | -0.018692213822661 | #N/A | | #N/A | #N/A |
| Cre09.g392208.v5.5 | -0.018668745590115 | #N/A | | #N/A | #N/A |
| Cre03.g194700.v5.5 | -0.018568347907587 | AGL1 | Alpha glucosidase | | Putative alpha-glucosidase (EC 3.2.1.20); similarity to arabidopsis [Aglu1; GenBank AAB82656] and sugar beet[GenBank BAA20343; PMID: 9178565] secreted alpha-glucosidase gene products |
| Cre12.g507300.v5.5 | -0.018535977372492 | LCI30 | Low-CO2-inducible protein | | Hypothetical protein highly conserved with protein on chromosome 5 of Arabidopsis and also with mammalian protein. Putative TGF (transforming growth factor) beta inducible nuclear protein TINP1 [Oryza sativa (japonica cultivar-group)].; May be associated |
| Cre03.g166150.v5.5 | -0.018484614597907 | #N/A | | #N/A | #N/A |
| Cre08.g384285.v5.5 | -0.018394553932041 | #N/A | | #N/A | #N/A |
| Cre06.g311050.v5.5 | -0.018353092980533 | #N/A | | #N/A | #N/A |
| Cre24.g755597.v5.5 | -0.018250815226267 | #N/A | | #N/A | #N/A |
| Cre17.g714750.v5.5 | -0.018226207681113 | #N/A | | #N/A | #N/A |
| Cre06.g278137.v5.5 | -0.018204881705646 | #N/A | | #N/A | #N/A |
| Cre14.g627950.v5.5 | -0.018187150949366 | #N/A | | #N/A | #N/A |
| Cre17.g732802.v5.5 | -0.01818019212802 | #N/A | | #N/A | #N/A |
| Cre09.g399912.v5.5 | -0.018052928316054 | #N/A | | #N/A | #N/A |
| Cre16.g668700.v5.5 | -0.017961039819676 | #N/A | | #N/A | #N/A |
| Cre12.g541450.v5.5 | -0.017868208201403 | #N/A | | #N/A | #N/A |
| Cre01.g029250.v5.5 | -0.017640238731458 | AAH1 | Aromatic amino acid hydroxylase-related protein | | Related to phenylalanine hydroxylase and tryptophan monooxygenase, not usually found in angiosperms |
| Cre07.g342552.v5.5 | -0.017631386684605 | #N/A | | #N/A | #N/A |
| Cre07.g323800.v5.5 | -0.017619830970618 | #N/A | | #N/A | #N/A |
| Cre17.g739350.v5.5 | -0.017581331654029 | #N/A | | #N/A | #N/A |
| Cre13.g605500.v5.5 | -0.017546639306976 | #N/A | | #N/A | #N/A |
| Cre02.g118100.v5.5 | -0.017546311115597 | #N/A | | #N/A | #N/A |
| Cre12.g498750.v5.5 | -0.017454886359882 | LIP2 | Triacylglycerol lipase | | Triacylglycerol lipase. Related to gastric ligase. Previously annotated as LIPG2 |
| Cre01.g013500.v5.5 | -0.01741983587509 | #N/A | | #N/A | #N/A |
| Cre08.g360200.v5.5 | -0.01735897614015 | DUR3 | Urea active transporter | | Na+/solute symporter. Previously annotated as DUR3A |
| Cre06.g310950.v5.5 | -0.017290198191368 | #N/A | | #N/A | #N/A |
| Cre10.g428966.v5.5 | -0.017192513633351 | #N/A | | #N/A | #N/A |
| Cre08.g364600.v5.5 | -0.017124153488005 | #N/A | | #N/A | #N/A |
| Cre12.g522250.v5.5 | -0.01710798041914 | #N/A | | #N/A | #N/A |
| Cre06.g249500.v5.5 | -0.017030661942123 | #N/A | Cytosine-C5 specific DNA methyltransferase | | Dnmt1 class (cytosine-5-)DNA methyltransferase, ChromDB DMT3403; allelic to the previously published sequence AB108536 at the DMT1 locus [also published as MET1; PMID: 11983892, PMID: 15514055], which was sequenced from strain CC-683, a strain carrying th |
| Cre08.g358600.v5.5 | -0.017019688005888 | #N/A | | #N/A | #N/A |
| Cre02.g074200.v5.5 | -0.016917876146775 | #N/A | | #N/A | #N/A |
| Cre05.g234653.v5.5 | -0.016904274739522 | #N/A | | #N/A | #N/A |
| Cre02.g106250.v5.5 | -0.016868605691789 | LAL2 | La-like RNA-binding protein | | La-like RNA-binding protein. Contains an N-terminal La domain and a RRM motif; its closest homologues in Arabidopsis are LA-like proteins |

| | | | | | |
|---|---|---|---|---|---|
| | | | | with a single RRM and an N-terminal extension (AT5G46250, AT3G19090), believed not to fulfill the stabilization func | |
| Cre06.g269908.v5.5 | -0.016826262114682 | #N/A | #N/A | | #N/A |
| Cre17.g710871.v5.5 | -0.016797543379032 | #N/A | #N/A | | #N/A |
| Cre16.g691150.v5.5 | -0.016691564055034 | #N/A | #N/A | | #N/A |
| Cre08.g368850.v5.5 | -0.016660880915618 | #N/A | #N/A | | #N/A |
| Cre06.g301750.v5.5 | -0.016637028161296 | #N/A | #N/A | | #N/A |
| Cre07.g325748.v5.5 | -0.016547462531392 | #N/A | #N/A | | #N/A |
| Cre08.g377550.v5.5 | -0.016537525967757 | #N/A | #N/A | | #N/A |
| Cre01.g029050.v5.5 | -0.016397388226313 | #N/A | #N/A | | #N/A |
| Cre06.g307750.v5.5 | -0.016394596414928 | #N/A | #N/A | | #N/A |
| Cre06.g278098.v5.5 | -0.016317510342748 | MCC1 | Methylcrotonoyl-CoA carboxylase alpha subunit | Methylcrotonoyl-CoA carboxylase subunit alpha, mitochondrial precursor; 3-methylcrotonyl-CoA carboxylase; biotin-dependent carboxylase. Previously annotated as MCCA. Contains a very short LSU-rRNA motif on the opposite strand (4456221-4456270), in intro | |
| Cre02.g099900.v5.5 | -0.016306662791335 | #N/A | #N/A | | #N/A |
| Cre11.g469800.v5.5 | -0.016249167382437 | #N/A | #N/A | | #N/A |
| Cre01.g004750.v5.5 | -0.016152860428728 | #N/A | #N/A | | #N/A |
| Cre17.g730350.v5.5 | -0.016093309341154 | #N/A | #N/A | | #N/A |
| Cre07.g355500.v5.5 | -0.01597037299039 | #N/A | #N/A | | #N/A |

**Table S11. The 100 genes contributing the most to the high acetate state including annotation.**

| 100 genes contributing most to the high acetate state | | | | |
|---|---|---|---|---|
| identifier | G0 | Gene Name | defline | description |
| Cre13.g569850.v5.5 | 0.061692255360913 | AMT4 | Ammonium transporter | Belongs to AMT1 family (AMT1;4 or AMT1.4); corresponds to AY542491, mRNA complete cds (PMID: 15821986). Previously annotated as AMT1D |
| Cre03.g177800.v5.5 | 0.057760166294872 | #N/A | #N/A | #N/A |
| Cre13.g563950.v5.5 | 0.052615672164696 | #N/A | #N/A | #N/A |
| Cre16.g690319.v5.5 | 0.050997473486197 | #N/A | #N/A | #N/A |
| Cre03.g204500.v5.5 | 0.050778844806041 | #N/A | #N/A | #N/A |
| Cre12.g551352.v5.5 | 0.050382528068324 | #N/A | #N/A | #N/A |
| Cre07.g321800.v5.5 | 0.048700331031005 | #N/A | #N/A | #N/A |
| Cre06.g253000.v5.5 | 0.048148326339707 | MTA1 | Gamete-specific protein | Protein of unknown function; the mRNA is induced in plus gametes; the gene exists only in the mt+ locus [PMID: 11805055]. The gene was originally called A1. Independently sequenced, see AF417571. |
| Cre04.g217944.v5.5 | 0.047981880431855 | #N/A | #N/A | #N/A |
| Cre01.g008300.v5.5 | 0.047883621966319 | #N/A | #N/A | #N/A |
| Cre17.g704100.v5.5 | 0.047395374636271 | #N/A | #N/A | #N/A |
| Cre02.g089500.v5.5 | 0.047209429364108 | #N/A | #N/A | #N/A |
| Cre10.g423850.v5.5 | 0.04714687361269 | SRR26 | Scavenger receptor cysteine rich (SRCR) protein | Scavenger receptor cysteine-rich protein; contains ligand-binding SRCR domain found in speract/scavenger receptor (PMID: 14995912) proteins. |
| Cre08.g382575.v5.5 | 0.046643645346606 | #N/A | #N/A | #N/A |
| Cre17.g714100.v5.5 | 0.046630335458602 | HTA21 | Histone H2A | Replication-linked Histone H2A |
| Cre09.g400750.v5.5 | 0.045659061228502 | AMT5 | Ammonium transporter | Belongs to AMT1 family (Amt1;5 or Amt1.5) two different splicing variants have been found for this gene, this one is AMT5b, the shorter one with additional intron (AY588243); was AMT1Eb; belongs to AMT1 family (Amt1;5 or Amt1.5); two different splicing va |
| Cre02.g081700.v5.5 | 0.045657504854803 | #N/A | #N/A | #N/A |
| Cre02.g077750.v5.5 | 0.045508174837251 | FAP211 | Flagellar Associated Protein | Flagellar Associated Protein, found in the flagellar proteome [PMID: 15998802] |
| Cre02.g102200.v5.5 | 0.045194819032043 | #N/A | #N/A | #N/A |
| Cre15.g635376.v5.5 | 0.04493704580035 | #N/A | #N/A | #N/A |
| Cre05.g235850.v5.5 | 0.044298841443097 | #N/A | #N/A | #N/A |
| Cre16.g686750.v5.5 | 0.043982211792315 | PTA3 | Proton/phosphate symporter | Putative phosphate transporter homolog type A-4, similar to yeast Pho84 H+/Pi symporter; clusters with PTA2 and PTA4 on scaffold 14. gi\|21218043\|dbj\|AB074876.1 |
| Cre12.g516700.v5.5 | 0.043981747920903 | #N/A | #N/A | #N/A |
| Cre10.g442800.v5.5 | 0.043964241655341 | XUV6 | Xanthine/uracil/vitamin C permease-like | Xanthine/uracil/vitamin C permease-like. Previously annotated as UAPA1 |
| Cre09.g390875.v5.5 | 0.043953908290605 | #N/A | #N/A | #N/A |
| Cre04.g220700.v5.5 | 0.04369856428454 | ALK2 | Aurora-like kinase | Possible role in mitotic regulation. Note that the ALK1 model (originally called CALK) and several other kinase models in the genome are much weaker hits to the aurora family. |
| Cre12.g559900.v5.5 | 0.043672195877131 | #N/A | #N/A | #N/A |
| Cre05.g238450.v5.5 | 0.043634396138197 | #N/A | #N/A | #N/A |
| Cre03.g204129.v5.5 | 0.043515039196616 | #N/A | #N/A | #N/A |
| Cre12.g549500.v5.5 | 0.043413626262504 | #N/A | #N/A | #N/A |
| Cre08.g360200.v5.5 | 0.04333950831122 | DUR3 | Urea active transporter | Na+/solute symporter. Previously annotated as DUR3A |
| Cre16.g668850.v5.5 | 0.043250290885929 | MTA2 | Hydroxyproline-rich glycoprotein | Hydroxyproline-rich glycoprotein, putative component of the zygote ECM; contains PPSPX repeats, described in PMID: 11258910. This gene is called A2 in some publications. The mRNA is gamete-specific; there is no protein localization data. A pseudogene copy |
| Cre12.g545703.v5.5 | 0.043078366443266 | #N/A | #N/A | #N/A |
| Cre01.g013769.v5.5 | 0.04269225070094 | #N/A | #N/A | #N/A |
| Cre13.g569150.v5.5 | 0.042076113255347 | #N/A | #N/A | #N/A |
| Cre05.g239700.v5.5 | 0.042009967190307 | #N/A | #N/A | #N/A |
| Cre14.g629920.v5.5 | 0.041970658842226 | #N/A | #N/A | #N/A |
| Cre09.g404201.v5.5 | 0.041825977180628 | #N/A | #N/A | #N/A |
| Cre08.g365150.v5.5 | 0.04165818651621 | #N/A | #N/A | #N/A |
| Cre02.g142366.v5.5 | 0.041384860459346 | #N/A | #N/A | #N/A |
| Cre08.g365065.v5.5 | 0.041279103338676 | #N/A | #N/A | #N/A |
| Cre11.g479950.v5.5 | 0.041231584367213 | #N/A | Triose phosphate transporter | Triose phosphate transporter. Conserved protein related to chloroplast P translocator |
| Cre48.g761247.v5.5 | 0.041043002732586 | #N/A | #N/A | #N/A |

| | | | | |
|---|---|---|---|---|
| Cre07.g320800.v5.5 | 0.040997674659227 | #N/A | #N/A | #N/A |
| Cre03.g207900.v5.5 | 0.040914039960444 | CYCA1 | A-type cyclin | Cyclin A homolog; A-type cyclin homolog. Putative function in S/G2/M |
| Cre17.g714500.v5.5 | 0.040783531336268 | HTA1 | Histone H2A | Replication-linked Histone H2A. PMID: 8590479. Identified as nitrogen-controlled-gene NCG10 expressed upon nitrogen starvation. PMID:11469597 |
| Cre07.g338000.v5.5 | 0.040726710631976 | MCM2 | Minichromosome maintenance protein | Homologous to MCM2 DNA replication protein |
| Cre14.g620702.v5.5 | 0.040481933781484 | #N/A | #N/A | #N/A |
| Cre08.g365100.v5.5 | 0.040358328093199 | FAP242 | Flagellar Associated Protein | Coiled-Coil Flagellar Associated Protein, found in the flagellar proteome [PMID: 15998802] |
| Cre11.g468800.v5.5 | 0.040308728638527 | #N/A | #N/A | #N/A |
| Cre01.g010300.v5.5 | 0.038957731407512 | #N/A | #N/A | #N/A |
| Cre09.g399363.v5.5 | 0.038820527992884 | #N/A | #N/A | #N/A |
| Cre17.g735550.v5.5 | 0.038142448918727 | #N/A | #N/A | #N/A |
| Cre17.g719050.v5.5 | 0.038129850925043 | #N/A | #N/A | #N/A |
| Cre02.g090150.v5.5 | 0.037935194072724 | PAN3 | Pantothenate synthetase | Pantothenate synthetase; Pantoate-beta-alanine ligase. Previously annotated as PANC |
| Cre17.g742250.v5.5 | 0.037889614494085 | CDKG2 | Cyclin dependent kinase | Cyclin dependent kinase; Chlamydomonas specific subfamily; AASTLRE motif. Novel CDK subfamily. Also identified from nitrogen starvation experiments as nitrogen-controlled-gene NCG9 [PMID: 11469597] |
| Cre03.g199090.v5.5 | 0.037755503845625 | #N/A | #N/A | #N/A |
| Cre09.g394065.v5.5 | 0.037447529418686 | #N/A | #N/A | #N/A |
| Cre06.g281750.v5.5 | 0.037388884671213 | #N/A | #N/A | #N/A |
| Cre06.g249500.v5.5 | 0.037359519462329 | #N/A | Cytosine-C5 specific DNA methyltransferase | Dnmt1 class (cytosine-5-)DNA methyltransferase, ChromDB DMT3403; allelic to the previously published sequence AB108536 at the DMT1 locus [also published as MET1; PMID: 11983892, PMID: 15514055], which was sequenced from strain CC-683, a strain carrying th |
| Cre02.g145950.v5.5 | 0.037327378657702 | #N/A | #N/A | #N/A |
| Cre17.g699800.v5.5 | 0.037291317274302 | #N/A | #N/A | #N/A |
| Cre09.g412400.v5.5 | 0.037263595226669 | #N/A | #N/A | #N/A |
| Cre05.g233350.v5.5 | 0.037159463750446 | #N/A | #N/A | #N/A |
| Cre11.g481750.v5.5 | 0.036997912664017 | GAS30 | Hydroxyproline-rich glycoprotein, stress-induced | Belongs to the large pherophorin-family, a family of extracellular matrix glycoproteins (cell wall glycoproteins) with a central hydroxyproline-rich (HR) domain. The mRNA is up-regulated in gametes, in zygotes, by agglutination/cAMP treatment, by osmotic |
| Cre06.g302050.v5.5 | 0.036948046300439 | #N/A | 1,3-beta-D-glucan synthase | 1,3-beta-D-glucan synthase and glycosyl transferase. Previously annotated as GTR12 |
| Cre11.g467586.v5.5 | 0.036887593916565 | #N/A | #N/A | #N/A |
| Cre07.g322250.v5.5 | 0.036867365815112 | #N/A | #N/A | #N/A |
| Cre17.g714150.v5.5 | 0.036716039049479 | KUP1 | Potassium ion uptake transporter | Potassium ion uptake transporter. Linked to related KUP2 and KUP3 |
| Cre12.g489700.v5.5 | 0.036685890670675 | OTC1 | Ornithine carbamoyltransferase | Ornithine carbamoyltransferase (OTC) (ARG3 in yeast); chloroplast-targeted (by homology to At1g75330); arg4 is probably a mutant in that gene; note however that arg4 is not rescued by citrulline, whereas an OTC mutant should be. |
| Cre06.g258850.v5.5 | 0.036684665678723 | #N/A | #N/A | #N/A |
| Cre03.g159254.v5.5 | 0.036604164877308 | #N/A | #N/A | #N/A |
| Cre04.g226550.v5.5 | 0.036508096315657 | #N/A | #N/A | #N/A |
| Cre06.g288100.v5.5 | 0.036206286196298 | #N/A | #N/A | #N/A |
| Cre04.g212450.v5.5 | 0.036185666064818 | #N/A | #N/A | #N/A |
| Cre06.g278250.v5.5 | 0.036002756917705 | #N/A | #N/A | #N/A |
| Cre10.g420200.v5.5 | 0.035891435729852 | #N/A | #N/A | #N/A |
| Cre17.g706000.v5.5 | 0.035854384890403 | #N/A | #N/A | #N/A |
| Cre12.g551353.v5.5 | 0.035799147330193 | #N/A | #N/A | #N/A |
| Cre13.g589450.v5.5 | 0.035664542073721 | #N/A | #N/A | #N/A |
| Cre12.g544450.v5.5 | 0.035627281537301 | #N/A | #N/A | #N/A |
| Cre08.g368650.v5.5 | 0.035520820478213 | #N/A | #N/A | #N/A |
| Cre03.g160250.v5.5 | 0.035518899708229 | #N/A | #N/A | #N/A |
| Cre01.g053000.v5.5 | 0.03548085387363 | GPD2 | Glycerol-3-phosphate dehydrogenase/dihydroxyacetone-3-phosphate reductase | Glycerol-3-phosphate dehydrogenase/dihydroxyacetone-3-phosphate reductase |
| Cre12.g524350.v5.5 | 0.035449666691746 | HUS1 | DNA damage checkpoint protein | Forms a sliding clamp complex with Rad9 and Rad1 that mediates repair at sites of DNA damage. |
| Cre11.g483351.v5.5 | 0.035364833585524 | #N/A | #N/A | #N/A |
| Cre08.g365103.v5.5 | 0.035035813567334 | #N/A | #N/A | #N/A |
| Cre13.g563050.v5.5 | 0.034963935381537 | #N/A | #N/A | #N/A |
| Cre07.g324500.v5.5 | 0.034812923576204 | #N/A | #N/A | #N/A |

| | | | | | |
|---|---|---|---|---|---|
| Cre07.g332275.v5.5 | 0.034789675800128 | | #N/A | #N/A | #N/A |
| Cre12.g488500.v5.5 | 0.034749173404391 | | #N/A | #N/A | #N/A |
| Cre12.g523900.v5.5 | 0.034587000479747 | | #N/A | #N/A | #N/A |
| Cre09.g405350.v5.5 | 0.034565146851294 | | #N/A | #N/A | #N/A |
| Cre03.g177600.v5.5 | 0.034470672864949 | | #N/A | #N/A | #N/A |
| Cre13.g605200.v5.5 | 0.034380590353668 | | #N/A | #N/A | #N/A |
| Cre07.g342551.v5.5 | 0.034246479004594 | | #N/A | #N/A | #N/A |
| Cre01.g053150.v5.5 | 0.034225330740729 | GPD3 | | Glycerol-3-phosphate dehydrogenase/dihydroxyacetone-3-phosphate reductase | Glycerol-3-phosphate dehydrogenase/dihydroxyacetone-3-phosphate reductase |
| Cre03.g190800.v5.5 | 0.034184717197988 | | #N/A | #N/A | #N/A |
| Cre12.g488450.v5.5 | 0.034165995801455 | VSP3 | | Extracellular matrix protein | Extracellular matrix protein (vegetative cell wall protein) similar to ISG (Volvox carteri) [PMID: 1600938], both are glycoproteins with a hydroxyproline-rich (HR) domain. [PMID: 8000007. ISG Genbank entry X65165; VSP3, L29029; C. incerta ortholog, AY7950 |
| Cre08.g385550.v5.5 | 0.034154628165592 | | #N/A | #N/A | #N/A |

## Table S12. The 100 genes contributing the most to the low acetate state including annotation.

**100 genes contributing most to the low acetate state**

| identifier | G0 | Gene Name | defline | description |
|---|---|---|---|---|
| Cre16.g661750.v5.5 | -0.054019122079341 | #N/A | #N/A | #N/A |
| Cre06.g274750.v5.5 | -0.040920443227048 | #N/A | Histone H2B | Replication-linked Histone H2B. Also annotated as HTB26 |
| Cre04.g223300.v5.5 | -0.040082055187707 | CCP1 | Low-CO2-inducible chloroplast envelope protein | Low-CO2-inducible 36kDa chloroplast envelope protein (LIP36-1), CO2-responsive gene; related to mitochondrial carriers; Chen et al. 1997 [PMID: 9159951]. CCP1 and LCIE are located in head-to-head orientation; regulated by CCM1 [PMID: 15235119]. Acclimatio |
| Cre05.g236650.v5.5 | -0.039720630250885 | #N/A | #N/A | #N/A |
| Cre04.g223100.v5.5 | -0.037952321087351 | CAH1 | Carbonic anhydrase | Carbonate dehydratase 1 in Chlamydomonas reinhardtii, also known as CA1; gi:115447; Carbonic anhydrase 1 precursor (Carbonate dehydratase 1) (CA1); alpha type - Carbonic anhydrase 1 localized in the periplasmic space and low-CO2 inducible gene regulated b |
| Cre11.g467615.v5.5 | -0.037922370340832 | #N/A | #N/A | #N/A |
| Cre06.g271550.v5.5 | -0.036248101030833 | #N/A | #N/A | #N/A |
| Cre16.g661850.v5.5 | -0.03594431122288 | #N/A | #N/A | #N/A |
| Cre05.g248400.v5.5 | -0.035911736348512 | CAH4 | Mitochondrial carbonic anhydrase, beta type | Mitochondrial carbonic anhydrase, beta type. Probably regulated by CCM1 [PMID: 15235119]; Acclimation to changing CO2 concentrations and light intensities was studied by Yamano et al. 2008 [PMID: 18322145], identified in their table as MCA. The inorganic |
| Cre17.g717400.v5.5 | -0.035665935540261 | #N/A | #N/A | #N/A |
| Cre05.g239600.v5.5 | -0.035124737862376 | #N/A | #N/A | #N/A |
| Cre14.g608093.v5.5 | -0.03445216943993 | #N/A | #N/A | #N/A |
| Cre09.g395288.v5.5 | -0.033847285887188 | NHA1 | Sodium ion/proton transporter protein | Sodium:hydrogen antiporter NhaD. Previously annotated as NHAD1 |
| Cre09.g393953.v5.5 | -0.032110499918333 | #N/A | #N/A | #N/A |
| Cre12.g554100.v5.5 | -0.031127312035748 | #N/A | #N/A | #N/A |
| Cre06.g265500.v5.5 | -0.030488940019009 | #N/A | #N/A | #N/A |
| Cre08.g367400.v5.5 | -0.030246260273157 | LHCSR3 | Stress-related chlorophyll a/b binding protein 3 | Stress-related chlorophyll a/b binding protein, similar to LI818r-1/LHCSR1. Peers et al. (2009) [PMID: 19940928] call this gene LHCSR3.2; this apparently codes for the same protein as LHCSR3.1 |
| Cre02.g085500.v5.5 | -0.029500384828609 | #N/A | #N/A | #N/A |
| Cre16.g683954.v5.5 | -0.029462157345945 | #N/A | #N/A | #N/A |
| Cre10.g436400.v5.5 | -0.029208393263428 | #N/A | #N/A | #N/A |
| Cre06.g257601.v5.5 | -0.028480704657127 | PRX1 | 2-cys peroxiredoxin, chloroplastic | 2-cys Peroxiredoxin, thioredoxin dependent peroxidase, chloroplast precursor, PMID: 11784321. Protein processed at aa 39 to produce N-terminus of SHAEKPL as determined by Edman degradation |
| Cre05.g248450.v5.5 | -0.027947979045768 | CAH5 | Mitochondrial carbonic anhydrase | Mitochondrial carbonic anhydrase, also called CA2. Matches exons 5 and 6 of CRU41190 Chlamydomonas reinhardtii carbonic anhydrase precursor (beta-CA2). The inorganic carbon-concentrating mechanism has been reviewed by Spalding (2008) [PMID: 17597098] |
| Cre02.g084350.v5.5 | -0.027802545686327 | CGLD1 | Predicted protein | Putative plastid protein |
| Cre09.g402219.v5.5 | -0.027739427116643 | LCI3 | Low-CO2-inducible protein | Regulated by CCM1 [PMID: 15235119]. Acclimation to changing CO2 concentrations and light intensities was studied by Yamano et al. 2008 [PMID: 18322145]. |
| Cre06.g268600.v5.5 | -0.027302269714369 | NAB1 | Nucleic acid binding protein | Nucleic acid binding protein; RNA binding protein involved in the light-regulated differential expression of the light-harvesting antenna of Chlamydomonas reinhardtii [PMID: 16284312, Genbank entry AY157846]. Studied by Wobbe et al. 2009 [PMID: 19666611] |
| Cre16.g649433.v5.5 | -0.027185128671149 | #N/A | #N/A | #N/A |
| Cre05.g244400.v5.5 | -0.027053516245648 | #N/A | #N/A | #N/A |
| Cre13.g590500.v5.5 | -0.026816023498735 | #N/A | Omega-6-fatty acid desaturase, chloroplast isoform | Omega-6-FAD, chloroplast isoform [PMID: 9498569]. Also known as DES6 |
| Cre10.g422300.v5.5 | -0.02676157159526 | PRX6 | Thioredoxin dependent peroxidase | Peroxiredoxin Q, chloroplastic thioredoxin dependent peroxidase, chloroplast precursor. Attached to thylakoids in Arabidopsis [PMID: 16507087; PMID: 15531707; PMID: 14976238; PMID: 12529539; PMID: 15890615] |
| Cre08.g367500.v5.5 | -0.026749524569554 | LHCSR2 | Stress-related chlorophyll a/b binding protein 2 | Stress-related chlorophyll a/b binding protein, similar to LI818r-1/LHCSR1. Peers et al. (2009) [PMID: 19940928] call this gene LHCSR3.1; this apparently codes for the same protein as LHCSR3.2 |
| Cre03.g205100.v5.5 | -0.026668440061125 | #N/A | #N/A | #N/A |
| Cre02.g082750.v5.5 | -0.02588328417187 | PSBX | 4.1 kDa photosystem II subunit | Encoding a 4.1 kDa subunit for PS2 with a single TMH [PMID: 16143838]. |
| Cre17.g730550.v5.5 | -0.025589387617479 | #N/A | #N/A | #N/A |
| Cre06.g309000.v5.5 | -0.025221321480339 | NAR1.2 | Anion transporter | Nitrite transporter-homologue, possibly chloroplastic [PMID: 11912227, AY612639]; significant similarities with two anion transporters, the chloroplastic nitrite transporter NAR1 and formate transporters in bacteria; also known as LCIA. nduced by CO2-limi |

| | | | | |
|---|---|---|---|---|
| Cre10.g436550.v5.5 | -0.025219816823229 | LCI5 | Low-CO2-inducible protein | Regulated by CCM1 [PMID: 15235119]. Acclimation to changing CO2 concentrations and light intensities was studied by Yamano et al. 2008 [PMID: 18322145]. |
| Cre08.g361150.v5.5 | -0.025199379733876 | #N/A | #N/A | #N/A |
| Cre13.g570851.v5.5 | -0.02511429017738 | #N/A | #N/A | #N/A |
| Cre06.g284600.v5.5 | -0.025100404062781 | RBD2 | Rubredoxin | Rubredoxin |
| Cre09.g393173.v5.5 | -0.024886886710214 | ELI2 | Early light-inducible protein | A homolog of ELIP (early light-inducible protein) gene which was originally identified as transiently expressed during the early phase of greening in etiolated pea seedlings [PMID: 6692824]. It has three TMH, whose sequences are closely related to LHC pro |
| Cre14.g621650.v5.5 | -0.024085396477217 | #N/A | #N/A | #N/A |
| Cre06.g271250.v5.5 | -0.023900399899252 | HTR8 | Histone H3 | Replication-linked Histone H3 |
| Cre13.g573250.v5.5 | -0.023885616757612 | #N/A | #N/A | #N/A |
| Cre04.g227700.v5.5 | -0.023723543686113 | #N/A | #N/A | #N/A |
| Cre12.g540351.v5.5 | -0.023656334169586 | #N/A | #N/A | #N/A |
| Cre09.g399908.v5.5 | -0.023274356939811 | #N/A | #N/A | #N/A |
| Cre02.g117175.v5.5 | -0.023145502197924 | #N/A | #N/A | #N/A |
| Cre02.g141786.v5.5 | -0.023070884389193 | PDE25 | 3',5'-cyclic-nucleotide phosphodiesterase | Calmodulin dependent PDE1B1 protein from Homo Sapiens is closest relative |
| Cre03.g195410.v5.5 | -0.022893590308439 | #N/A | #N/A | #N/A |
| Cre26.g756747.v5.5 | -0.022446823611193 | #N/A | #N/A | #N/A |
| Cre14.g610400.v5.5 | -0.02242292916985 | #N/A | #N/A | #N/A |
| Cre06.g288900.v5.5 | -0.022366681834002 | #N/A | #N/A | #N/A |
| Cre17.g732250.v5.5 | -0.022356458174544 | #N/A | #N/A | #N/A |
| Cre03.g180450.v5.5 | -0.02222906564093 | #N/A | 5'-nucleotidase and Flagellar Associated Protein | 5'-nucleotidase and conserved uncharacterized Flagellar Associated Protein; found in the flagellar proteome [PMID: 15998802]. Previously annotated as FAP215 |
| Cre16.g650250.v5.5 | -0.022144510134672 | HFO39 | Histone H4 | Replication-linked Histone H4 |
| Cre03.g195200.v5.5 | -0.022082119500335 | #N/A | Haloalkane dehalogenase-like hydrolase | Haloalkane dehalogenase, an alpha/beta hydrolast. Green-lineage conserved expressed protein with epoxide hydrolase / esterase motif. Previously annotated as CGL76 |
| Cre12.g485438.v5.5 | -0.021937903206539 | #N/A | #N/A | #N/A |
| Cre16.g672609.v5.5 | -0.021779493531215 | #N/A | #N/A | #N/A |
| Cre13.g571520.v5.5 | -0.021591768598954 | #N/A | #N/A | #N/A |
| Cre07.g334550.v5.5 | -0.021435755434016 | PSAO | Photosystem I subunit O | Photosystem I subunit O; similar to 10-kDa subunit of eukaryotic photosystem I [PMID: 11801243]; microarray element: 894019E07 |
| Cre14.g626700.v5.5 | -0.021202150904888 | PETF | Ferredoxin | 2Fe-2S containing redox protein involved in photosynthetic electron transfer, chloroplast localization [PMID: 16656453] |
| Cre09.g413200.v5.5 | -0.021046649492915 | #N/A | Serine/threonine protein kinase | Serine/Threonine protein kinase. Previously annotated as STPK22 |
| Cre13.g603550.v5.5 | -0.020905830560223 | #N/A | #N/A | #N/A |
| Cre11.g477733.v5.5 | -0.02075707991942 | #N/A | #N/A | #N/A |
| Cre08.g358558.v5.5 | -0.020743283567865 | #N/A | #N/A | #N/A |
| Cre14.g613800.v5.5 | -0.020727379700084 | #N/A | #N/A | #N/A |
| Cre01.g025150.v5.5 | -0.020586154775489 | CPLD15 | Predicted protein | Conserved organelle protein with lipase active site |
| Cre13.g565311.v5.5 | -0.020485822017577 | #N/A | #N/A | #N/A |
| Cre24.g755197.v5.5 | -0.020425338769253 | RBD3 | Putative rubredoxin-like protein | Putative rubredoxin-like protein |
| Cre12.g546150.v5.5 | -0.020400170256475 | PETM | Cytochrome b6f complex PetM subunit | Cytochrome b6f complex chain PetM, chloroplast precursor; GI:2493687; PMID: 8631873, PMID: 8616155, PMID: 7493968 |
| Cre13.g590750.v5.5 | -0.020144350381864 | HTB37 | Histone H2B | Replication-linked Histone H2B |
| Cre06.g273150.v5.5 | -0.020063931852885 | #N/A | #N/A | #N/A |
| Cre06.g274700.v5.5 | -0.020048876896628 | #N/A | #N/A | #N/A |
| Cre01.g010900.v5.5 | -0.020047832755341 | GAP3 | Glyceraldehyde-3-Phosphate Dehydrogenase | Glyceraldehyde 3-phosphate dehydrogenase A, chloroplast precursor (NADP-dependent glyceraldehyde phosphate dehydrogenase subunit A); corresponds to G3PA_CHLRE; found in the flagellar proteome [PMID: 15998802]. This enzyme is bispecific for NADP and NAD, |
| Cre16.g694500.v5.5 | -0.019843518083654 | #N/A | DnaJ-like protein | DnaJ-like protein |
| Cre10.g435800.v5.5 | -0.019521949980616 | #N/A | NAD-dependent epimerase/dehydratase | NAD-dependent epimerase/dehydratase, possibly with UDP-glucose 4-epimerase activity. Possible chloroplast stem-loop-binding protein 41b, cp ribosome-associated protein RAP38. Previously annotated as CSP41b |
| Cre16.g662600.v5.5 | -0.019495986597625 | #N/A | #N/A | #N/A |
| Cre09.g392867.v5.5 | -0.019489822746428 | FMG1-B | Flagella membrane glycoprotein, major form | Flagella membrane protein, major form. Only one expressed in Chlamydomonas during vegetative growth (and likely even in gametes). A gene model for this was present in v2, lost in v3, and recovered with Augustus (first noticed in Au5 update). This g |
| Cre02.g118550.v5.5 | -0.019384856416699 | #N/A | #N/A | #N/A |
| Cre11.g479900.v5.5 | -0.01935289596692 | #N/A | #N/A | #N/A |
| Cre02.g076250.v5.5 | -0.019315977311994 | EFG1 | Chloroplast elongation factor G | Putative chloroplast elongation factor G, (fusA), GTP binding; likely locus for previously described C. reinhardtii fsr1 (fus-1) mutant |

| | | | | |
|---|---|---|---|---|
| | | | | resistant to fusidic acid (Carbonera et al. 1981, FEBS Lett. 132, 227-230) |
| Cre13.g580000.v5.5 | -0.019286458448033 | #N/A | #N/A | #N/A |
| Cre08.g362100.v5.5 | -0.019226279510258 | #N/A | Similar to Flagellar Associated Protein FAP154 | Similar to Flagellar Associated Protein FAP154 with PAS sensory domain that was found in the flagellar proteome [PMID: 15998802]. This may be a duplication of FAP154; overlaps FAP154 gene model from v3 |
| Cre17.g716613.v5.5 | -0.019217126718573 | #N/A | #N/A | #N/A |
| Cre12.g494850.v5.5 | -0.019102669807173 | ADK3 | Adenylate kinase | Adenylate kinase; probably chloroplast-targeted (by homology to KADD_ARATH); contains a C-terminal domain with two Cys residues, homologous to that found in CP12 linking GAPDH and PRK [PMID: 8980547] |
| Cre17.g734725.v5.5 | -0.018925232552117 | #N/A | #N/A | #N/A |
| Cre12.g550850.v5.5 | -0.018901346748483 | PSBP1 | Oxygen-evolving enhancer protein 2 of photosystem II | Oxygen-evolving enhancer protein 2, chloroplast precursor (OEE2) (23 kDa subunit of oxygen evolving complex of photosystem II) (OEC 23 kDa subunit) (23 kDa thylakoid membrane protein) (OEC23);PMID: 3468511; targeted to thylakoid lumen by TAT pathway |
| Cre14.g630500.v5.5 | -0.018868137929669 | #N/A | #N/A | #N/A |
| Cre16.g687900.v5.5 | -0.018829786157859 | LHCA7 | Light-harvesting protein of photosystem I | #N/A |
| Cre06.g272650.v5.5 | -0.0188227595825 | LHCA8 | Light-harvesting protein of photosystem I | #N/A |
| Cre17.g715250.v5.5 | -0.01872897479201 | BCC1 | Acetyl-CoA biotin carboxyl carrier | Biotin-containing subunit of the chloroplastic acetyl-coenzyme A carboxylase; N-terminal sequence identified by Edman degradation is AAKT.... |
| Cre04.g229536.v5.5 | -0.018668394080494 | #N/A | #N/A | #N/A |
| Cre30.g758247.v5.5 | -0.018613225768048 | #N/A | #N/A | #N/A |
| Cre17.g724300.v5.5 | -0.018595939640892 | PSAK | Photosystem I reaction center subunit psaK | 8.4 kD subunit of photosystem I (polypeptide 37) [PMID: 2693938] |
| Cre10.g439450.v5.5 | -0.018577484500267 | #N/A | #N/A | #N/A |
| Cre12.g517681.v5.5 | -0.018503104482783 | #N/A | #N/A | #N/A |
| Cre11.g479100.v5.5 | -0.018446195005692 | #N/A | #N/A | #N/A |
| Cre03.g156050.v5.5 | -0.018364483020047 | #N/A | #N/A | #N/A |
| Cre12.g486300.v5.5 | -0.018341711035851 | PSAL | Photosystem I reaction center subunit XI | #N/A |
| Cre17.g720250.v5.5 | -0.018333822887126 | LHCB4 | Chlorophyll a/b binding protein of photosystem II | Chlorophyll a-b binding protein of photosystem II. Protein product also known as CP29 |
| Cre16.g679750.v5.5 | -0.018283244057131 | #N/A | #N/A | #N/A |

**Table S13. The 100 most stable regulatory genes (Protein Kinase (PPC), transcriptional regulators and transcription factors from the iTAK database) including annotation.**

**$G_0$ sorted**

| identifier | $G_0$ | geneName | defline | description | name | type |
|---|---|---|---|---|---|---|
| Cre17.g742250.v5.5 | 0.01041097245589 | CDKG2 | Cyclin dependent kinase | Cyclin dependent kinase; Chlamydomonas specific subfamily; AASTLRE motif. Novel CDK subfamily. Also identified from nitrogen starvation experiments as nitrogen-controlled-gene NCG9 [PMID: 11469597] | CDC2 Like Kinase Family | PPC |
| Cre12.g537400.v5.5 | 0.008975762021363 | | | | Unknown Function Kinase | PPC |
| Cre02.g087900.v5.5 | 0.008517079870007 | | | | GmPK6/AtMRK1 Family | PPC |
| Cre03.g199050.v5.5 | 0.007667285114936 | | | | IRE/NPH/PI dependent/S6 Kinase | PPC |
| Cre09.g404351.v5.5 | 0.004374692184443 | | | | SET | transcriptional regulator |
| Cre11.g467585.v5.5 | 0.004098069082157 | | | | Unknown Function Kinase | PPC |
| Cre01.g040150.v5.5 | 0.002847075293308 | | | | WNK like kinase - with no lysine kinase | PPC |
| Cre03.g187300.v5.5 | 0.002735088965977 | | | | Unknown Function Kinase | PPC |
| Cre11.g475100.v5.5 | 0.002397327965001 | | | | GNAT | transcriptional regulator |
| Cre17.g710000.v5.5 | 0.00219402281228 | | | | GmPK6/AtMRK1 Family | PPC |
| Cre09.g392300.v5.5 | 0.002049160165836 | | | | GNAT | transcriptional regulator |
| Cre03.g182700.v5.5 | 0.001995879628658 | | | | Others | transcriptional regulator |
| Cre04.g221900.v5.5 | 0.001988289255229 | | | | TRAF | transcriptional regulator |
| Cre09.g396065.v5.5 | 0.001477899973287 | ALK5 | Aurora-like kinase | Aurora-like kinase | Unknown Function Kinase | PPC |
| Cre03.g193900.v5.5 | 0.001431393329984 | | High mobility group protein, histone-like transcription factor | High mobility group protein, histone-like transcription factor. Has homology to DNA polymerase epsilon subunit | HMG | transcriptional regulator |
| Cre01.g036900.v5.5 | 0.001406308390395 | | | | CTR1/EDR1 Kinase | PPC |
| Cre01.g009550.v5.5 | 0.001398986719746 | | | | GNAT | transcriptional regulator |
| Cre13.g564100.v5.5 | 0.001227531617411 | PTK2 | Putative tyrosine kinase | Putative tyrosine kinase identified through multi-level HMM library for kinase classification (PMID: 17557329) | GmPK6/AtMRK1 Family | PPC |
| Cre02.g141886.v5.5 | 0.001120333419833 | | | | Receptor Like Cytoplasmic Kinase VI | PPC |
| Cre07.g346550.v5.5 | 0.00110089267122 | | | | GmPK6/AtMRK1 Family | PPC |
| Cre03.g212977.v5.5 | 0.001097555154075 | | | | TAZ | transcriptional regulator |
| Cre12.g522400.v5.5 | 0.000968871816103 | | | | MYB->MYB | transcription factor |
| Cre09.g404050.v5.5 | 0.00096155299405 | | | | GmPK6/AtMRK1 Family | PPC |
| Cre03.g149400.v5.5 | 0.000867665028949 | RWP11 | RWP-RK transcription factor | Putative RWP-RK domain transcription factor [PMID: 15785851]. Tandem pair with RWP4 with which there is amino acid homology around the RWP-RK domain and some intron conservation. Part of the model is tentative. | RWP-RK | transcription factor |
| Cre03.g153050.v5.5 | 0.000832014625406 | RWP1 | RWP-RK transcription factor | Putative RWP-RK domain transcription factor [PMID: 15785851]; model is very tentative | RWP-RK | transcription factor |
| Cre11.g467586.v5.5 | 0.000802498836637 | | | | Leucine Rich Repeat Kinase XI & XII | PPC |
| Cre03.g174150.v5.5 | 0.000758383160517 | | | | TRAF | transcriptional regulator |
| Cre06.g296200.v5.5 | 0.000680302938973 | | | | Calcium Dependent Protein Kinase | PPC |

| | | | | | | |
|---|---|---|---|---|---|---|
| Cre16.g674065.v5.5 | 0.000659419919695 | | | | Unknown Function Kinase | PPC |
| Cre17.g741900.v5.5 | 0.000611332851719 | | | | GmPK6/AtMRK1 Family | PPC |
| Cre16.g695600.v5.5 | 0.000603702350472 | DNJ10 | DnaJ-like protein and Myb-like transcription factor | DnaJ-like protein and Myb-like transcription factor; probably nuclear/cytosolic; similar to Chlamy Q6JX09_CHLRE GlsA-related protein and to Volvox GLSA; contains J-domain (pfam00226) at the N-terminus and Myb_DNA-binding-Domain (pfam00249) at the C-termin | MYB->MYB-related | transcription factor |
| Cre03.g198800.v5.5 | 0.00059970392788 | | | | MYB->MYB-related | transcription factor |
| Cre12.g500900.v5.5 | 0.000572617608828 | | | | PHD | transcriptional regulator |
| Cre06.g281000.v5.5 | 0.000554160134835 | | | | SET | transcriptional regulator |
| Cre11.g479800.v5.5 | 0.000499613088264 | | | | TRAF | transcriptional regulator |
| Cre13.g583217.v5.5 | 0.000445885477054 | | | | GNAT | transcriptional regulator |
| Cre14.g632700.v5.5 | 0.000445486354914 | | | | GmPK6/AtMRK1 Family | PPC |
| Cre03.g149201.v5.5 | 0.000414498173279 | | | | MYB->MYB | transcription factor |
| Cre03.g151050.v5.5 | 0.000399184765747 | | | | TRAF | transcriptional regulator |
| Cre06.g278265.v5.5 | 0.000394102435955 | | | | Unknown Function Kinase | PPC |
| Cre06.g249050.v5.5 | 0.000369830647426 | | | | MYB->MYB-related | transcription factor |
| Cre01.g048501.v5.5 | 0.000363116408629 | | | | TRAF | transcriptional regulator |
| Cre09.g413050.v5.5 | 0.00034191802918 | | | | bZIP | transcription factor |
| Cre04.g223500.v5.5 | 0.000303538132762 | | | | IRE/NPH/PI dependent/S6 Kinase | PPC |
| Cre10.g435750.v5.5 | 0.000277896379824 | | Serine/Threonine protein kinase | Serine/Threonine protein kinase. Previously annotated as two models, STPK21 and PTK26 (for protein tyrosine kinase) | S Domain Kinase (Type 2) | PPC |
| Cre07.g339100.v5.5 | 0.000263287665261 | | | | Unknown Function Kinase | PPC |
| Cre10.g423200.v5.5 | 0.000250619291515 | | | | GmPK6/AtMRK1 Family | PPC |
| Cre16.g679893.v5.5 | 0.00024222034113 | | | | GmPK6/AtMRK1 Family | PPC |
| Cre08.g361400.v5.5 | 0.000211782404239 | | | | CPP | transcription factor |
| Cre16.g679781.v5.5 | 0.000202345081449 | | | | SET | transcriptional regulator |
| Cre16.g676300.v5.5 | 0.000189597638901 | | | | CTR1/EDR1 Kinase | PPC |
| Cre14.g623926.v5.5 | 0.000181476854579 | | | | GmPK6/AtMRK1 Family | PPC |
| Cre06.g289900.v5.5 | 0.00015150859582 | | | | MYB->MYB-related | transcription factor |
| Cre09.g398750.v5.5 | 0.000144576027389 | | | | GmPK6/AtMRK1 Family | PPC |
| Cre10.g446850.v5.5 | 0.00012999954551 | | | | Jumonji | transcriptional regulator |
| Cre13.g579650.v5.5 | 0.000116187358545 | | | | GmPK6/AtMRK1 Family | PPC |
| Cre12.g558300.v5.5 | 0.000112337818414 | | | | GmPK6/AtMRK1 Family | PPC |
| Cre17.g742700.v5.5 | 6.00227749121812E-05 | | Histone-lysine N-methyltransferase | Histone-lysine N-methyltransferase. Green-lineage-and-diatoms conserved protein contains a SET domain with similarity to Arabidopsis thaliana ATXR3, SET2, SDG2 (At4g15180) and Oryza sativa SDG701. Previously annotated as CGLD30. ChromoDB SDG3401. Poss | SET | transcriptional regulator |
| Cre12.g485600.v5.5 | 3.92449728784609E-05 | | | | SNF1 Related Protein Kinase (SnRK) | PPC |
| Cre02.g084550.v5.5 | 1.83366549863245E-05 | | | | GNAT | transcriptional regulator |
| Cre17.g745447.v5.5 | 1.1736317940689E-05 | MAPK4 | Mitogen-Activated Protein Kinase | Hypothetical Mitogen-Activated Protein Kinase Homolog 4 | MAPK Family | PPC |
| Cre16.g649700.v5.5 | -3.70053686262777E-05 | | | | SET | transcriptional regulator |
| Cre06.g297150.v5.5 | -3.72629725971905E-05 | RWP9 | RWP-RK transcription factor | Putative RWP-RK domain transcription factor; model is very tentative | RWP-RK | transcription factor |
| Cre08.g385300.v5.5 | -5.22266199978947E-05 | | | | SET | transcriptional regulator |

| | | | | | | |
|---|---|---|---|---|---|---|
| Cre03.g169100.v5.5 | -5.7092248941272E-05 | | | | Calcium Dependent Protein Kinase | PPC |
| Cre11.g482700.v5.5 | -6.20517468967167E-05 | | | | HMG | transcriptional regulator |
| Cre12.g541777.v5.5 | -6.79695198535387E-05 | | | | SET | transcriptional regulator |
| Cre07.g353000.v5.5 | -8.22281675553122E-05 | | | | CDC2 Like Kinase Family | PPC |
| Cre02.g111950.v5.5 | -0.000106612282938 | | | | GmPK6/AtMRK1 Family | PPC |
| Cre17.g747397.v5.5 | -0.000113598036014 | | | | SNF1 Related Protein Kinase (SnRK) | PPC |
| Cre04.g228850.v5.5 | -0.000159677180812 | | | | Unknown Function Kinase | PPC |
| Cre07.g340250.v5.5 | -0.00016443144292 | | Serine/threonine protein kinase | Serine/Threonine protein kinase. Previously annotated as STPK8 | GmPK6/AtMRK1 Family | PPC |
| Cre17.g738600.v5.5 | -0.000169517826765 | RLS5 | RegA/RlsA-like protein | Member of a family of proteins [PMID: 16720695, 16622701] related to Volvox carteri RegA, which is a putative transcription repressor [PMID: 9895312] containing a SAND domain (IPR000770 SAND; PF01342). | VARL | transcription factor |
| Cre12.g514250.v5.5 | -0.000184383320232 | | | | ARID | transcriptional regulator |
| Cre11.g467577.v5.5 | -0.00018927664782 | | | | MADS->MADS-M-type | transcription factor |
| Cre13.g583150.v5.5 | -0.000195184448698 | | | | TRAF | transcriptional regulator |
| Cre12.g495100.v5.5 | -0.000201080193084 | PSR1 | Phosphorus starvation response protein, Myb-like transcriptional regulator | Myb-like DNA binding domain and coiled-coil domain containing transcription factor required for phosphate deficiency-inducible gene expression; nuclear-localized [AF174480 (mRNA), AF174532 (gene); PMID: 10611385] | GARP->GARP-G2-like | transcription factor |
| Cre01.g023100.v5.5 | -0.000265974543283 | | | | AP2/ERF->AP2/ERF-ERF | transcription factor |
| Cre06.g302150.v5.5 | -0.0002948867918 | | | | VARL | transcription factor |
| Cre10.g464264.v5.5 | -0.000304949630244 | | | | GNAT | transcriptional regulator |
| Cre02.g083750.v5.5 | -0.000308953213645 | | | | GARP->GARP-G2-like | transcription factor |
| Cre14.g633789.v5.5 | -0.000336585456635 | | | | MYB->MYB-related | transcription factor |
| Cre03.g160700.v5.5 | -0.000342610305972 | | | | C2C2->C2C2-GATA | transcription factor |
| Cre09.g393062.v5.5 | -0.000352145182434 | | | | GmPK6/AtMRK1 Family | PPC |
| Cre06.g264400.v5.5 | -0.000389891953958 | | | | MYB->MYB-related | transcription factor |
| Cre10.g425350.v5.5 | -0.000391465295682 | | | | Legume Lectin Domain Kinase | PPC |
| Cre04.g219400.v5.5 | -0.000429581454051 | | | | TRAF | transcriptional regulator |
| Cre09.g390300.v5.5 | -0.000440910377274 | | | | GmPK6/AtMRK1 Family | PPC |
| Cre03.g201332.v5.5 | -0.000452380211715 | | | | SET | transcriptional regulator |
| Cre17.g709900.v5.5 | -0.00045463495611 | | | | GmPK6/AtMRK1 Family | PPC |
| Cre03.g186450.v5.5 | -0.000488355403593 | | Lysine N-methylase | Putative SET domain lysine N-methylase, possibly involved in histone modification. ChromoDB SDG3414 | SET | transcriptional regulator |
| Cre16.g653258.v5.5 | -0.000492505083718 | | | | TRAF | transcriptional regulator |
| Cre09.g387950.v5.5 | -0.000500106323684 | | | | GmPK6/AtMRK1 Family | PPC |
| Cre14.g617151.v5.5 | -0.000501050154693 | | | | VARL | transcription factor |
| Cre13.g581150.v5.5 | -0.000519148538138 | | | | GNAT | transcriptional regulator |
| Cre12.g552150.v5.5 | -0.000599821271365 | | | | GmPK6/AtMRK1 Family | PPC |
| Cre13.g604350.v5.5 | -0.000604134233214 | | | | GmPK6/AtMRK1 Family | PPC |
| Cre16.g694550.v5.5 | -0.000604879259672 | | | | CTR1/EDR1 Kinase | PPC |
| Cre09.g394400.v5.5 | -0.000613320827055 | | | | GmPK6/AtMRK1 Family | PPC |
| Cre11.g481104.v5.5 | -0.000657628702137 | | | | Calcium Dependent Protein Kinase | PPC |

**Table S14. The 100 regulatory genes (Protein Kinase (PPC), transcriptional regulators and transcription factors from iTAK) contributing the most to the stationary state including annotation.**

**$G_1$ sorted**

| identifier | $G_1$ | geneName | defline | description | name | type |
|---|---|---|---|---|---|---|
| Cre12.g537400.v5.5 | -0.0153139799596 | | | | Unknown Function Kinase | PPC |
| Cre14.g623926.v5.5 | -0.014037006930749 | | | | GmPK6/AtMRK1 Family | PPC |
| Cre17.g700550.v5.5 | -0.014028236140407 | | | | TRAF | transcriptional regulator |
| Cre02.g087850.v5.5 | -0.013976687167056 | PTK19 | Protein tyrosine kinase | Putative protein tyrosine kinase identified through multi-level HMM library for kinase classification (PMID: 17557329) | GmPK6/AtMRK1 Family | PPC |
| Cre01.g022950.v5.5 | -0.013813258535296 | | | | TRAF | transcriptional regulator |
| Cre02.g146629.v5.5 | -0.013582671144331 | | | | MYB->MYB-related | transcription factor |
| Cre09.g396065.v5.5 | -0.013195390689521 | ALK5 | Aurora-like kinase | Aurora-like kinase | Unknown Function Kinase | PPC |
| Cre07.g330650.v5.5 | -0.013065600052316 | | | | mTERF | transcriptional regulator |
| Cre07.g322450.v5.5 | -0.013048875869342 | | | | SET | transcriptional regulator |
| Cre03.g144747.v5.5 | -0.013037898584037 | | | | MYB->MYB | transcription factor |
| Cre04.g221900.v5.5 | -0.012742978771397 | | | | TRAF | transcriptional regulator |
| Cre08.g384250.v5.5 | -0.012361622963537 | | | | SNF1 Related Protein Kinase (SnRK) | PPC |
| Cre13.g579200.v5.5 | -0.012246720946781 | S6K1 | Ribosomal protein S6 kinase | Protein kinase that phosphorylates rpS6, mediating ribosomal processivity; activation is TOR-dependent | IRE/NPH/PI dependent/S6 Kinase | PPC |
| Cre12.g558300.v5.5 | -0.012222969092559 | | | | GmPK6/AtMRK1 Family | PPC |
| Cre06.g278265.v5.5 | -0.012045943763679 | | | | Unknown Function Kinase | PPC |
| Cre03.g173165.v5.5 | -0.012043120975247 | | | | TRAF | transcriptional regulator |
| Cre06.g262800.v5.5 | -0.0115004632112 | | | | PHD | transcriptional regulator |
| Cre16.g666300.v5.5 | -0.010748454059189 | | | | CTR1/EDR1 Kinase | PPC |
| Cre09.g395658.v5.5 | -0.010360815447916 | | | | CDC2 Like Kinase Family | PPC |
| Cre12.g554051.v5.5 | -0.010351788575901 | | | | Leucine Rich Repeat Kinase XI & XII | PPC |
| Cre03.g212641.v5.5 | -0.0102192552022 | | | | TAZ | transcriptional regulator |
| Cre17.g713750.v5.5 | -0.010091968072999 | | Serine/threonine protein kinase | Serine/Threonine protein kinase. Previously annotated as STPK16 | Leucine Rich Repeat Kinase XI & XII | PPC |
| Cre17.g715700.v5.5 | -0.009973716207746 | | Phosphoprotein phosphatase 2C | Phosphoprotein phosphatase 2C. Previously annotated as PP2C5 | Leucine Rich Repeat Kinase XI & XII | PPC |
| Cre02.g074850.v5.5 | -0.009973296925905 | | | | Unknown Function Kinase | PPC |
| Cre10.g425350.v5.5 | -0.009916285411942 | | | | Legume Lectin Domain Kinase | PPC |
| Cre02.g108350.v5.5 | -0.00974188645036 | | | | MYB->MYB | transcription factor |
| Cre03.g174200.v5.5 | -0.009720532250253 | | | | TRAF | transcriptional regulator |
| Cre02.g111200.v5.5 | -0.009568225446873 | | | | MYB->MYB-related | transcription factor |
| Cre06.g253250.v5.5 | -0.009446911674628 | | | | MADS->MADS-M-type | transcription factor |
| Cre16.g674291.v5.5 | -0.009368281300386 | | | | Unknown Function Kinase | PPC |
| Cre02.g141966.v5.5 | -0.009342031776893 | | | | Other Protein Kinase | PPC |
| Cre09.g410450.v5.5 | -0.009276865481252 | | | | GARP->GARP-G2-like | transcription factor |
| Cre10.g423200.v5.5 | -0.009236112009984 | | | | GmPK6/AtMRK1 Family | PPC |
| Cre03.g146067.v5.5 | -0.009201916377806 | | | | Receptor Like Cytoplasmic Kinase VII | PPC |
| Cre03.g169100.v5.5 | -0.009160690696399 | | | | Calcium Dependent Protein Kinase | PPC |
| Cre13.g566200.v5.5 | -0.00910756841831 | | | | C3H | transcription factor |
| Cre16.g684450.v5.5 | -0.009016392387422 | | | | SNF1 Related Protein Kinase (SnRK) | PPC |
| Cre11.g481104.v5.5 | -0.008988630319524 | | | | Calcium Dependent Protein Kinase | PPC |
| Cre03.g187300.v5.5 | -0.008964876052803 | | | | Unknown Function Kinase | PPC |
| Cre04.g228800.v5.5 | -0.008948543205183 | | | | Unknown Function Kinase | PPC |
| Cre12.g527000.v5.5 | -0.008947861378061 | | | | Calcium Dependent Protein Kinase | PPC |

| | | | | | | |
|---|---|---|---|---|---|---|
| Cre01.g040150.v5.5 | -0.008831965899736 | | | | WNK like kinase - with no lysine kinase | PPC |
| Cre12.g489000.v5.5 | -0.008797969618033 | | | | bZIP | transcription factor |
| Cre02.g113600.v5.5 | -0.008742108768305 | | | | Unknown Function Kinase | PPC |
| Cre02.g108650.v5.5 | -0.008653709081121 | | | | GmPK6/AtMRK1 Family | PPC |
| Cre08.g378800.v5.5 | -0.008640000779984 | | | | C2C2->C2C2-GATA | transcription factor |
| Cre07.g349000.v5.5 | -0.00850084482242 | | | | GmPK6/AtMRK1 Family | PPC |
| Cre16.g674403.v5.5 | -0.008475010356836 | | | | Unknown Function Kinase | PPC |
| Cre03.g167850.v5.5 | -0.008442356799696 | | | | TRAF | transcriptional regulator |
| Cre05.g236216.v5.5 | -0.008419516724107 | | | | CTR1/EDR1 Kinase | PPC |
| Cre02.g111550.v5.5 | -0.008347964848881 | | | | GmPK6/AtMRK1 Family | PPC |
| Cre01.g016570.v5.5 | -0.008320275341583 | | | | MAP3K | PPC |
| Cre02.g096700.v5.5 | -0.00830655193286 | | | | TRAF | transcriptional regulator |
| Cre09.g391245.v5.5 | -0.008282322230684 | | | | APG1 Like Kinase | PPC |
| Cre07.g314500.v5.5 | -0.008180370305148 | | | | GmPK6/AtMRK1 Family | PPC |
| Cre09.g388875.v5.5 | -0.008146633058495 | | | | GmPK6/AtMRK1 Family | PPC |
| Cre17.g746247.v5.5 | -0.008110023975363 | | | | SET | transcriptional regulator |
| Cre09.g407373.v5.5 | -0.008068967983454 | | | | GNAT | transcriptional regulator |
| Cre13.g575300.v5.5 | -0.008025247910782 | | | | Leucine Rich Repeat Kinase VII | PPC |
| Cre06.g303150.v5.5 | -0.007952757912858 | PTK4 | Putative tyrosine kinase | Putative tyrosine kinase identified through multi-level HMM library for kinase classification (PMID: 17557329) | Other Protein Kinase | PPC |
| Cre03.g198800.v5.5 | -0.007942807877213 | | | | MYB->MYB-related | transcription factor |
| Cre17.g719450.v5.5 | -0.007915148855948 | | | | Unknown Function Kinase | PPC |
| Cre07.g335150.v5.5 | -0.007864031689273 | | | | SBP | transcription factor |
| Cre13.g572150.v5.5 | -0.007860617988991 | | | | GNAT | transcriptional regulator |
| Cre01.g046237.v5.5 | -0.007853050587333 | | | | SNF2 | transcriptional regulator |
| Cre12.g514250.v5.5 | -0.007778703987475 | | | | ARID | transcriptional regulator |
| Cre02.g113350.v5.5 | -0.007744675509264 | | | | GmPK6/AtMRK1 Family | PPC |
| Cre09.g390171.v5.5 | -0.007732186954417 | | | | GmPK6/AtMRK1 Family | PPC |
| Cre02.g098600.v5.5 | -0.007723510774605 | | | | GmPK6/AtMRK1 Family | PPC |
| Cre14.g611200.v5.5 | -0.007608718306512 | | | | mTERF | transcriptional regulator |
| Cre11.g467586.v5.5 | -0.007564301791407 | | | | Leucine Rich Repeat Kinase XI & XII | PPC |
| Cre16.g687800.v5.5 | -0.007549492200267 | | | | GmPK6/AtMRK1 Family | PPC |
| Cre10.g425000.v5.5 | -0.007546712637035 | | | | GmPK6/AtMRK1 Family | PPC |
| Cre01.g030600.v5.5 | -0.007492223112921 | MAPKKK4 | Mitogen-Activated Protein Kinase Kinase Kinase | MAPKKK4; MAP (Mitogen-Activated) Kinase Kinase Kinase Homolog 4; MEK Kinase 4; MAP3K4 | GmPK6/AtMRK1 Family | PPC |
| Cre02.g117813.v5.5 | -0.007485562450212 | | | | GmPK6/AtMRK1 Family | PPC |
| Cre06.g249700.v5.5 | -0.00744459001675 | | | | mTERF | transcriptional regulator |
| Cre09.g393650.v5.5 | -0.00744205763404 | | | | GmPK6/AtMRK1 Family | PPC |
| Cre16.g687400.v5.5 | -0.007439836681871 | | | | MAPK Family | PPC |
| Cre11.g467581.v5.5 | -0.007403829468578 | | | | C2C2->C2C2-GATA | transcription factor |
| Cre03.g144484.v5.5 | -0.007401156645307 | | | | Calcium Dependent Protein Kinase | PPC |
| Cre02.g102650.v5.5 | -0.007344295373998 | | | | CTR1/EDR1 Kinase | PPC |
| Cre12.g551250.v5.5 | -0.007336626358299 | | | | CDC2 Like Kinase Family | PPC |
| Cre01.g014500.v5.5 | -0.007326156010086 | | | | GmPK6/AtMRK1 Family | PPC |
| Cre10.g446850.v5.5 | -0.007270314828662 | | | | Jumonji | transcriptional regulator |
| Cre12.g516600.v5.5 | -0.007259396365441 | | Serine/threonine protein kinase | Serine/Threonine protein kinase | S Domain Kinase (Type 2) | PPC |
| Cre03.g189650.v5.5 | -0.007242073207127 | | | | TAZ | transcriptional regulator |
| Cre09.g387023.v5.5 | -0.007207878577996 | | | | CTR1/EDR1 Kinase | PPC |
| Cre01.g012200.v5.5 | -0.007189387202965 | | | | SBP | transcription factor |
| Cre02.g145500.v5.5 | -0.007182366018145 | PTK24 | Protein tyrosine kinase | Putative protein tyrosine kinase identified through | GmPK6/AtMRK1 Family | PPC |

| | | | | | | |
|---|---|---|---|---|---|---|
| | | | | multi-level HMM library for kinase classification (PMID: 17557329) | | |
| Cre11.g480950.v5.5 | -0.007126399120634 | | | | HMG | transcriptional regulator |
| Cre05.g236400.v5.5 | -0.007111411456278 | | | | GNAT | transcriptional regulator |
| Cre10.g436100.v5.5 | -0.007086173398982 | MLK1 | Mixed lineage protein kinase | Mixed lineage protein kinase with a GAF domain, which is a domain present in phytochrome and CGMP-specific phosphodiesterases. A serine/threonine protein kinase which has a GAF domain, a potential chromophore binding domain. | CTR1/EDR1 Kinase | PPC |
| Cre03.g176651.v5.5 | -0.007036590984859 | | | | MYB->MYB-related | transcription factor |
| Cre03.g169500.v5.5 | -0.006968449818209 | | | | CDC2 Like Kinase Family | PPC |
| Cre12.g551201.v5.5 | -0.006958075665117 | | | | GNAT | transcriptional regulator |
| Cre14.g630750.v5.5 | -0.006936886965706 | MAPKKK10 | Mitogen-Activated Protein Kinase Kinase Kinase | MAP3K10 | GmPK6/AtMRK1 Family | PPC |
| Cre12.g530000.v5.5 | -0.006935350214384 | | | | MAPK Family | PPC |
| Cre03.g145407.v5.5 | -0.006879727167364 | | | | C3H | transcription factor |
| Cre04.g230438.v5.5 | -0.006876403391352 | | Serine/threonine protein kinase | Serine/threonine protein kinase. Previously annotated as SRR25 | GmPK6/AtMRK1 Family | PPC |
| Cre04.g211600.v5.5 | -0.006824962617891 | | | | SNF1 Related Protein Kinase (SnRK) | PPC |

**Table S14. The 100 regulatory genes (Protein Kinase (PPC), transcriptional regulators and transcription factors from the iTAK database) contributing the most to the exponential state including annotation.**

**$G_1$ sorted**

| identifier | $G_1$ | geneName | defline | description | name | type |
|---|---|---|---|---|---|---|
| Cre06.g268600.v5.5 | 0.039908483445275 | NAB1 | Nucleic acid binding protein | Nucleic acid binding protein; RNA binding protein involved in the light-regulated differential expression of the light-harvesting antenna of Chlamydomonas reinhardtii [PMID: 16284312, Genbank entry AY157846]. Studied by Wobbe et al. 2009 [PMID: 19666611] | CSD | transcription factor |
| Cre08.g372550.v5.5 | 0.035563502305416 | CDKB1 | Plant specific cyclin dependent kinase | Plant specific cyclin dependent kinase; CDKB subfamily; PSTTLRE motif; mitotic regulator. Putative function in G2/M | CDC2 Like Kinase Family | PPC |
| Cre11.g482700.v5.5 | 0.033612210891776 | | | | HMG | transcriptional regulator |
| Cre02.g101850.v5.5 | 0.031070869487763 | | | | GNAT | transcriptional regulator |
| Cre06.g249050.v5.5 | 0.030183593199477 | | | | MYB->MYB-related | transcription factor |
| Cre07.g355250.v5.5 | 0.028273139105171 | WEE1 | CDK inhibitory kinase | WEE1 kinase, CDK inhibitory kinase, dual specificity for serine/threonine and tyrosine. Putative function in G2/M | Unknown Function Kinase | PPC |
| Cre03.g193900.v5.5 | 0.027693079946812 | | High mobility group protein, histone-like transcription factor | High mobility group protein, histone-like transcription factor. Has homology to DNA polymerase epsilon subunit | HMG | transcriptional regulator |
| Cre09.g404351.v5.5 | 0.026791782506516 | | | | SET | transcriptional regulator |
| Cre14.g623800.v5.5 | 0.026162507109669 | | | | GNAT | transcriptional regulator |
| Cre02.g084550.v5.5 | 0.025910433782552 | | | | GNAT | transcriptional regulator |
| Cre16.g664000.v5.5 | 0.025337358268807 | | | | SET | transcriptional regulator |
| Cre09.g386350.v5.5 | 0.025234838850502 | | | | IWS1 | transcriptional regulator |
| Cre09.g392300.v5.5 | 0.024804574025502 | | | | GNAT | transcriptional regulator |
| Cre08.g383150.v5.5 | 0.024464248392691 | NAT5 | N-acetyl-transferase-like protein | Contains similarity to N-acetyl-transferase 5 that catalyzes the first step of melatonin biosynthesis | GNAT | transcriptional regulator |
| Cre11.g475100.v5.5 | 0.023966496934803 | | | | GNAT | transcriptional regulator |
| Cre17.g710000.v5.5 | 0.022629722541365 | | | | GmPK6/AtMRK1 Family | PPC |
| Cre12.g560200.v5.5 | 0.022216320224439 | NAT2 | Glucosamine 6-phosphate N-acetyltransferase | Glucosamine 6-phosphate N-acetyltransferase. N-acetyltransferase related to GCN5, a histone acetyltransferase | GNAT | transcriptional regulator |
| Cre16.g649700.v5.5 | 0.022037439373744 | | | | SET | transcriptional regulator |
| Cre06.g304050.v5.5 | 0.022034103795788 | | | | IRE/NPH/PI dependent/S6 Kinase | PPC |
| Cre11.g467659.v5.5 | 0.021979422748045 | | | | MED6 | transcriptional regulator |
| Cre12.g541777.v5.5 | 0.021190311122856 | | | | SET | transcriptional regulator |
| Cre16.g679893.v5.5 | 0.020610979058654 | | | | GmPK6/AtMRK1 Family | PPC |
| Cre17.g696300.v5.5 | 0.020596959512559 | | | | C2H2 | transcription factor |
| Cre13.g574300.v5.5 | 0.020484673312893 | | | | GmPK6/AtMRK1 Family | PPC |
| Cre03.g185950.v5.5 | 0.020429852944815 | | | | C2H2 | transcription factor |
| Cre10.g446450.v5.5 | 0.020376339125738 | | | | Others | transcriptional regulator |
| Cre01.g062902.v5.5 | 0.019477568346997 | | | | MED7 | transcriptional regulator |
| Cre06.g296200.v5.5 | 0.018912691095976 | | | | Calcium Dependent Protein Kinase | PPC |
| Cre17.g745447.v5.5 | 0.018304189223532 | MAPK4 | Mitogen-Activated Protein Kinase | Hypothetical Mitogen-Activated Protein Kinase Homolog 4 | MAPK Family | PPC |
| Cre13.g583150.v5.5 | 0.018079120923344 | | | | TRAF | transcriptional regulator |

| | | | | | | |
|---|---|---|---|---|---|---|
| Cre09.g399552.v5.5 | 0.017540041480506 | LCR1 | Low-CO2 response regulator, Myb-like transcription factor | Myb-DNA binding transcriptional factor induced under low CO2 stress condition [PMID: 15155888]; regulates CO2-responsive genes, Cah1, Lci1, and Lci6; regulated by CCM1 [PMID: 15235119] | MYB->MYB-related | transcription factor |
| Cre01.g035150.v5.5 | 0.017300174655929 | | | | C3H | transcription factor |
| Cre02.g141886.v5.5 | 0.017039048729257 | | | | Receptor Like Cytoplasmic Kinase VI | PPC |
| Cre06.g281000.v5.5 | 0.016808522946978 | | | | SET | transcriptional regulator |
| Cre01.g036900.v5.5 | 0.016554583618468 | | | | CTR1/EDR1 Kinase | PPC |
| Cre13.g572600.v5.5 | 0.015685849133613 | | | | SET | transcriptional regulator |
| Cre04.g220700.v5.5 | 0.015573876084009 | ALK2 | Aurora-like kinase | Possible role in mitotic regulation. Note that the ALK1 model (originally called CALK) and several other kinase models in the genome are much weaker hits to the aurora family. | Unknown Function Kinase | PPC |
| Cre10.g429880.v5.5 | 0.015236286129495 | | | | ARID | transcriptional regulator |
| Cre16.g653258.v5.5 | 0.014480545791998 | | | | TRAF | transcriptional regulator |
| Cre11.g479800.v5.5 | 0.0144310459798 | | | | TRAF | transcriptional regulator |
| Cre03.g151050.v5.5 | 0.014415456539851 | | | | TRAF | transcriptional regulator |
| Cre07.g346550.v5.5 | 0.014157329633439 | | | | GmPK6/AtMRK1 Family | PPC |
| Cre09.g386753.v5.5 | 0.013175490225302 | | | | S1Fa-like | transcription factor |
| Cre11.g467568.v5.5 | 0.013165977452692 | | | | IRE/NPH/PI dependent/S6 Kinase | PPC |
| Cre03.g174150.v5.5 | 0.012783797897047 | | | | TRAF | transcriptional regulator |
| Cre06.g293750.v5.5 | 0.012525763735153 | | | | C3H | transcription factor |
| Cre01.g038300.v5.5 | 0.011684828924645 | | | | mTERF | transcriptional regulator |
| Cre12.g522400.v5.5 | 0.011160977899072 | | | | MYB->MYB | transcription factor |
| Cre16.g657150.v5.5 | 0.01101988050037 | | | | GNAT | transcriptional regulator |
| Cre12.g483650.v5.5 | 0.010985338671687 | | | | CDC2 Like Kinase Family | PPC |
| Cre08.g364450.v5.5 | 0.010604522469592 | NAT1 | Peptide alpha-N-acetyltransferase | Peptide alpha-N-acetyltransferase | GNAT | transcriptional regulator |
| Cre11.g467577.v5.5 | 0.010084139266638 | | | | MADS->MADS-M-type | transcription factor |
| Cre12.g514400.v5.5 | 0.009959907677424 | | | | MYB->MYB-related | transcription factor |
| Cre03.g184600.v5.5 | 0.009735486613113 | RWP7 | RWP-RK transcription factor | Putative RWP-RK domain transcription factor [PMID: 15785851]. The model is partly based on an unpublished cDNA sequence. | RWP-RK | transcription factor |
| Cre17.g738600.v5.5 | 0.009668530174794 | RLS5 | RegA/RlsA-like protein | Member of a family of proteins [PMID: 16720695, 16622701] related to Volvox carteri RegA, which is a putative transcription repressor [PMID: 9895312] containing a SAND domain (IPR000770 SAND; PF01342). | VARL | transcription factor |
| Cre03.g152150.v5.5 | 0.009628239590313 | | | | C2H2 | transcription factor |
| Cre17.g741900.v5.5 | 0.009523467962478 | | | | GmPK6/AtMRK1 Family | PPC |
| Cre11.g469400.v5.5 | 0.00933063889287 | | | | Others | transcriptional regulator |
| Cre06.g261450.v5.5 | 0.009177111710038 | | | | HMG | transcriptional regulator |
| Cre03.g160700.v5.5 | 0.008641167583391 | | | | C2C2->C2C2-GATA | transcription factor |
| Cre12.g509000.v5.5 | 0.008610992303841 | MAPK3 | Mitogen-Activated Protein Kinase | Mitogen-Activated Protein Kinase Homolog 3; downstream kinase in the canonical MAP kinase pathway | MAPK Family | PPC |
| Cre02.g083750.v5.5 | 0.008418137238237 | | | | GARP->GARP-G2-like | transcription factor |
| Cre13.g564100.v5.5 | 0.008321980401547 | PTK2 | Putative tyrosine kinase | Putative tyrosine kinase identified through multi-level HMM library for kinase classification (PMID: 17557329) | GmPK6/AtMRK1 Family | PPC |
| Cre17.g702650.v5.5 | 0.00816436039123 | | | | HMG | transcriptional regulator |

| | | | | | | |
|---|---|---|---|---|---|---|
| Cre07.g329900.v5.5 | 0.008100937101493 | | | | Others | transcriptional regulator |
| Cre06.g250950.v5.5 | 0.007465533037693 | | | | C3H | transcription factor |
| Cre09.g414650.v5.5 | 0.007453161915058 | | | | MAPK Family | PPC |
| Cre24.g755647.v5.5 | 0.007404580992485 | | | | C3H | transcription factor |
| Cre09.g393728.v5.5 | 0.007153142101581 | | | | SET | transcriptional regulator |
| Cre13.g580450.v5.5 | 0.007098461746361 | | | | TRAF | transcriptional regulator |
| Cre14.g614750.v5.5 | 0.007000082618185 | | | | GNAT | transcriptional regulator |
| Cre01.g009550.v5.5 | 0.00697787481571 | | | | GNAT | transcriptional regulator |
| Cre10.g441000.v5.5 | 0.006615330409794 | | | | IWS1 | transcriptional regulator |
| Cre16.g668200.v5.5 | 0.006569799502889 | | | | PHD | transcriptional regulator |
| Cre16.g679781.v5.5 | 0.006549082382677 | | | | SET | transcriptional regulator |
| Cre16.g695600.v5.5 | 0.006065239052822 | DNJ10 | DnaJ-like protein and Myb-like transcription factor | DnaJ-like protein and Myb-like transcription factor; probably nuclear/cytosolic; similar to Chlamy Q6JX09_CHLRE GlsA-related protein and to Volvox GLSA; contains J-domain (pfam00226) at the N-terminus and Myb_DNA-binding-Domain (pfam00249) at the C-termin | MYB->MYB-related | transcription factor |
| Cre08.g384900.v5.5 | 0.006051425181334 | | | | CTR1/EDR1 Kinase | PPC |
| Cre09.g413200.v5.5 | 0.00601748891563 | | Serine/threonine protein kinase | Serine/Threonine protein kinase. Previously annotated as STPK22 | CDC2 Like Kinase Family | PPC |
| Cre12.g495100.v5.5 | 0.005995003197363 | PSR1 | Phosphorus starvation response protein, Myb-like transcriptional regulator | Myb-like DNA binding domain and coiled-coil domain containing transcription factor required for phosphate deficiency-inducible gene expression; nuclear-localized [AF174480 (mRNA), AF174532 (gene); PMID: 10611385] | GARP->GARP-G2-like | transcription factor |
| Cre07.g351800.v5.5 | 0.005922088588887 | PTK12 | Protein tyrosine kinase | Putative protein tyrosine kinase identified through multi-level HMM library for kinase classification (PMID: 17557329) | Leucine Rich Repeat Kinase XI & XII | PPC |
| Cre04.g217943.v5.5 | 0.005831391164752 | | | | SNF1 Related Protein Kinase (SnRK) | PPC |
| Cre12.g516150.v5.5 | 0.005604776246164 | | | | GNAT | transcriptional regulator |
| Cre10.g464100.v5.5 | 0.005570306505474 | | | | MAP3K | PPC |
| Cre12.g552150.v5.5 | 0.005450527856794 | | | | GmPK6/AtMRK1 Family | PPC |
| Cre03.g152500.v5.5 | 0.005418958848952 | | | | SET | transcriptional regulator |
| Cre02.g087900.v5.5 | 0.005389371956976 | | | | GmPK6/AtMRK1 Family | PPC |
| Cre06.g278184.v5.5 | 0.005262334037598 | | | | TRAF | transcriptional regulator |
| Cre16.g657350.v5.5 | 0.005211155683758 | | | | SNF1 Related Protein Kinase (SnRK) | PPC |
| Cre15.g643700.v5.5 | 0.004881063623466 | RLS6 | RegA/RlsA-like protein | Member of a family of proteins [PMID: 16720695, 16622701] related to Volvox carteri RegA, which is a putative transcription repressor [PMID: 9895312] containing a SAND domain (IPR000770 SAND; PF01342) | VARL | transcription factor |
| Cre02.g114750.v5.5 | 0.004838103865249 | | | | Calcium Dependent Protein Kinase | PPC |
| Cre10.g461150.v5.5 | 0.004730929539725 | | | | Unknown Function Kinase | PPC |
| Cre01.g002250.v5.5 | 0.004729128330598 | | | | GNAT | transcriptional regulator |
| Cre03.g149201.v5.5 | 0.004690048643646 | | | | MYB->MYB | transcription factor |
| Cre05.g241636.v5.5 | 0.004628246484063 | | | | bHLH | transcription factor |
| Cre06.g275500.v5.5 | 0.004571205294254 | | | | AP2/ERF->AP2/ERF-ERF | transcription factor |
| Cre04.g229948.v5.5 | 0.004508260969292 | | | | C3H | transcription factor |
| Cre06.g264400.v5.5 | 0.004493653030072 | | | | MYB->MYB-related | transcription factor |

| | | | |
|---|---|---|---|
| Cre07.g334250.v5.5 | 0.004438307594955 | SET | transcriptional regulator |
| Cre13.g581150.v5.5 | 0.004433757486765 | GNAT | transcriptional regulator |
| Cre10.g441300.v5.5 | 0.004411205748618 | AP2/ERF->AP2/ERF-AP2 | transcription factor |

**Table S16. The 100 regulatory genes (Protein Kinase (PPC), transcriptional regulators and transcription factors from the iTAK database) contributing the most to the low acetate state including annotation.**

| $G_2$ sorted identifier | $G_2$ | geneName | defline | description | name | type |
|---|---|---|---|---|---|---|
| Cre06.g268600.v5.5 | -0.027302269714369 | NAB1 | Nucleic acid binding protein | Nucleic acid binding protein; RNA binding protein involved in the light-regulated differential expression of the light-harvesting antenna of Chlamydomonas reinhardtii [PMID: 16284312, Genbank entry AY157846]. Studied by Wobbe et al. 2009 [PMID: 19666611] | CSD | transcription factor |
| Cre09.g413200.v5.5 | -0.021046649492915 | | Serine/threonine protein kinase | Serine/Threonine protein kinase. Previously annotated as STPK22 | CDC2 Like Kinase Family | PPC |
| Cre17.g736250.v5.5 | -0.018223227537334 | | | | GmPK6/AtMRK1 Family | PPC |
| Cre17.g746547.v5.5 | -0.016249335901147 | | | | bZIP | transcription factor |
| Cre12.g534100.v5.5 | -0.016193518159343 | | | | Others | transcriptional regulator |
| Cre12.g501600.v5.5 | -0.015124413229956 | | | | bZIP | transcription factor |
| Cre01.g036250.v5.5 | -0.014328805837334 | | | | SNF1 Related Protein Kinase (SnRK) | PPC |
| Cre02.g141886.v5.5 | -0.013957054506091 | | | | Receptor Like Cytoplasmic Kinase VI | PPC |
| Cre09.g391245.v5.5 | -0.013424886692029 | | | | APG1 Like Kinase | PPC |
| Cre13.g604350.v5.5 | -0.013331687571319 | | | | GmPK6/AtMRK1 Family | PPC |
| Cre01.g036900.v5.5 | -0.013235999161154 | | | | CTR1/EDR1 Kinase | PPC |
| Cre12.g514400.v5.5 | -0.013135346823895 | | | | MYB->MYB-related | transcription factor |
| Cre02.g120250.v5.5 | -0.01297102375006 | | Calcium/calmodulin-dependent protein kinase | Chloroplast protein kinase required for LHCII phosphorylation and state transition [PMID: 12624266]. Identified in the eyespot phosphoproteome by Wagner et al. 2008 [PMID: 18065559]. | GSK3/Shaggy Like Protein Kinase Family | PPC |
| Cre10.g438850.v5.5 | -0.012691686899621 | | | | bZIP | transcription factor |
| Cre17.g696950.v5.5 | -0.012505381418891 | | | | CTR1/EDR1 Kinase | PPC |
| Cre13.g574300.v5.5 | -0.012405643730999 | | | | GmPK6/AtMRK1 Family | PPC |
| Cre02.g141966.v5.5 | -0.012396296290734 | | | | Other Protein Kinase | PPC |
| Cre13.g579650.v5.5 | -0.012314850197403 | | | | GmPK6/AtMRK1 Family | PPC |
| Cre09.g407373.v5.5 | -0.01225238653361 | | | | GNAT | transcriptional regulator |
| Cre09.g404050.v5.5 | -0.01115596269779 | | | | GmPK6/AtMRK1 Family | PPC |
| Cre16.g677382.v5.5 | -0.011091748087841 | | | | MYB->MYB | transcription factor |
| Cre14.g628237.v5.5 | -0.011020858940682 | | | | GmPK6/AtMRK1 Family | PPC |
| Cre14.g623800.v5.5 | -0.010908756141582 | | | | GNAT | transcriptional regulator |
| Cre12.g521150.v5.5 | -0.010830271484854 | CGL19 | Predicted protein | Conserved expressed protein with Dof type zinc finger | C2C2->C2C2-Dof | transcription factor |
| Cre01.g064362.v5.5 | -0.010746413189694 | | | | GmPK6/AtMRK1 Family | PPC |
| Cre10.g446450.v5.5 | -0.010596984000128 | | | | Others | transcriptional regulator |
| Cre02.g102650.v5.5 | -0.010469666812559 | | | | CTR1/EDR1 Kinase | PPC |
| Cre17.g707650.v5.5 | -0.010402589999402 | | | | SNF1 Related Protein Kinase (SnRK) | PPC |
| Cre08.g368700.v5.5 | -0.010319015633982 | | | | SET | transcriptional regulator |
| Cre01.g030600.v5.5 | -0.010313735045169 | MAPKKK4 | Mitogen-Activated Protein Kinase Kinase Kinase | MAPKKK4; MAP (Mitogen-Activated) Kinase Kinase Kinase Homolog 4; MEK Kinase 4; MAP3K4 | GmPK6/AtMRK1 Family | PPC |
| Cre13.g603600.v5.5 | -0.010292462091995 | | | | GmPK6/AtMRK1 Family | PPC |
| Cre14.g633789.v5.5 | -0.010223854894053 | | | | MYB->MYB-related | transcription factor |
| Cre04.g219600.v5.5 | -0.010223435035738 | | | | TRAF | transcriptional regulator |
| Cre01.g009500.v5.5 | -0.010168612017048 | | | | Calcium Dependent Protein Kinase | PPC |
| Cre06.g293300.v5.5 | -0.009941098217323 | | | | GmPK6/AtMRK1 Family | PPC |
| Cre09.g399552.v5.5 | -0.009689302228528 | LCR1 | Low-CO2 response regulator, Myb-like transcription factor | Myb-DNA binding transcriptional factor induced under low CO2 stress condition [PMID: 15155888]; regulates CO2-responsive genes, Cah1, Lci1, and Lci6; regulated by CCM1 [PMID: 15235119] | MYB->MYB-related | transcription factor |

| | | | | | | |
|---|---|---|---|---|---|---|
| Cre02.g106650.v5.5 | -0.009644378292513 | | | | Calcium Dependent Protein Kinase | PPC |
| Cre16.g674403.v5.5 | -0.009387021995572 | | | | Unknown Function Kinase | PPC |
| Cre16.g684400.v5.5 | -0.009171921358055 | | | | CDC2 Like Kinase Family | PPC |
| Cre17.g719450.v5.5 | -0.009153569432369 | | | | Unknown Function Kinase | PPC |
| Cre02.g094150.v5.5 | -0.009091012961225 | | | | Pseudo ARR-B | transcriptional regulator |
| Cre09.g411950.v5.5 | -0.008985472638715 | | | | MYB->MYB-related | transcription factor |
| Cre06.g278159.v5.5 | -0.008893021565106 | CON1 | CONSTANS-like protein involved in circadian rhythms | B-box zinc finger protein; contains CONSTANS-type zinc finger, conserved in Ostreococcus and involved in circadian rhythms. Previously annotated as CGLD18 for being conserved in the Green lineage and diatoms, as well as CO for CONSTANS by Serrano et al. | C2C2->C2C2-CO-like | transcription factor |
| Cre06.g275350.v5.5 | -0.00888302160813 | | | | MYB->MYB-related | transcription factor |
| Cre16.g649700.v5.5 | -0.008836324387629 | | | | SET | transcriptional regulator |
| Cre07.g346550.v5.5 | -0.008814478568924 | | | | GmPK6/AtMRK1 Family | PPC |
| Cre06.g291500.v5.5 | -0.008785813463926 | RWP6 | RWP-RK transcription factor | Putative RWP-RK domain transcription factor, however the domain is non-canonical in this gene (NWPsRK). The model is based on an unpublished cDNA sequence. | RWP-RK | transcription factor |
| Cre14.g632750.v5.5 | -0.008596876626975 | | | | GmPK6/AtMRK1 Family | PPC |
| Cre04.g212100.v5.5 | -0.008511059293967 | | | | TRAF | transcriptional regulator |
| Cre06.g253200.v5.5 | -0.008508846081655 | | Protein tyrosine kinase | Protein tyrosine kinase. Previously annotated as PKY1 | GmPK6/AtMRK1 Family | PPC |
| Cre10.g418900.v5.5 | -0.008414450733 | | | | Calcium Dependent Protein Kinase | PPC |
| Cre03.g185950.v5.5 | -0.008348510621441 | | | | C2H2 | transcription factor |
| Cre02.g108350.v5.5 | -0.0083204398687 | | | | MYB->MYB | transcription factor |
| Cre12.g535100.v5.5 | -0.008220041356359 | | | | Unknown Function Kinase | PPC |
| Cre07.g334950.v5.5 | -0.008218586476192 | | | | GmPK6/AtMRK1 Family | PPC |
| Cre17.g741900.v5.5 | -0.008126861634032 | | | | GmPK6/AtMRK1 Family | PPC |
| Cre17.g717100.v5.5 | -0.007981998690485 | | | | GmPK6/AtMRK1 Family | PPC |
| Cre03.g168150.v5.5 | -0.007935450648337 | | | | Ankyrin Repeat Domain Kinase | PPC |
| Cre13.g568050.v5.5 | -0.007878854692396 | | | | SNF1 Related Protein Kinase (SnRK) | PPC |
| Cre09.g390134.v5.5 | -0.007863637406822 | | | | GmPK6/AtMRK1 Family | PPC |
| Cre03.g212641.v5.5 | -0.007818519350491 | | | | TAZ | transcriptional regulator |
| Cre06.g275500.v5.5 | -0.00765115277588 | | | | AP2/ERF->AP2/ERF-ERF | transcription factor |
| Cre04.g223500.v5.5 | -0.007638105844873 | | | | IRE/NPH/PI dependent/S6 Kinase | PPC |
| Cre06.g304050.v5.5 | -0.007612500089536 | | | | IRE/NPH/PI dependent/S6 Kinase | PPC |
| Cre04.g228800.v5.5 | -0.007542158896205 | | | | Unknown Function Kinase | PPC |
| Cre07.g345350.v5.5 | -0.007472833742064 | | | | MYB->MYB | transcription factor |
| Cre03.g157200.v5.5 | -0.007368151766934 | | | | GmPK6/AtMRK1 Family | PPC |
| Cre04.g230438.v5.5 | -0.007364921018793 | | Serine/threonine protein kinase | Serine/threonine protein kinase. Previously annotated as SRR25 | GmPK6/AtMRK1 Family | PPC |
| Cre07.g314500.v5.5 | -0.007295585184967 | | | | GmPK6/AtMRK1 Family | PPC |
| Cre06.g278265.v5.5 | -0.00723095279127 | | | | Unknown Function Kinase | PPC |
| Cre02.g141946.v5.5 | -0.007212205384675 | | | | GmPK6/AtMRK1 Family | PPC |
| Cre16.g679893.v5.5 | -0.007101367987538 | | | | GmPK6/AtMRK1 Family | PPC |
| Cre16.g659400.v5.5 | -0.007011279564238 | | | | GmPK6/AtMRK1 Family | PPC |
| Cre17.g710000.v5.5 | -0.006903614474124 | | | | GmPK6/AtMRK1 Family | PPC |
| Cre02.g096300.v5.5 | -0.006888842365639 | CCM1 | Regulator of $CO_2$-responsive genes | Master regulator of $CO_2$-responsive genes including low-$CO_2$ inducible genes necessary for carbon-concentrating mechanism; CCM1-A and CCM1-B, are generated by an alternative splicing between 3rd and 4th exons, CCM1B=CIA5=698aa, CCM1A=699aa, Zn-finger domain | C2H2 | transcription factor |
| Cre08.g381950.v5.5 | -0.006883370556937 | | | | Unknown Function Kinase | PPC |
| Cre08.g378800.v5.5 | -0.006817237538094 | | | | C2C2->C2C2-GATA | transcription factor |

| | | | | | | |
|---|---|---|---|---|---|---|
| Cre02.g107800.v5.5 | -0.006791650764685 | CNK9 | NimA-related protein kinase | Contains a NIMA (never-in-mitosis) kinase domain and a C-terminal region with no database similarity except in Volvox | Unknown Function Kinase | PPC |
| Cre04.g211600.v5.5 | -0.006751758501643 | | | | SNF1 Related Protein Kinase (SnRK) | PPC |
| Cre02.g074150.v5.5 | -0.006710321246721 | COP5 | Chlamoypsin, light-gated proton channel rhodopsin | Chlamyopsin 5, sensory rhodopsin with histidine kinase domain; has histidine kinase, response regulator receiver domains like a two-component hybrid sensor and regulator [PMID: 15143209] | Others | transcriptional regulator |
| Cre10.g462350.v5.5 | -0.006671127727761 | | | | GmPK6/AtMRK1 Family | PPC |
| Cre17.g700166.v5.5 | -0.006594659331304 | | | | WNK like kinase - with no lysine kinase | PPC |
| Cre06.g296200.v5.5 | -0.00658872868935 | | | | Calcium Dependent Protein Kinase | PPC |
| Cre06.g278200.v5.5 | -0.006575462559263 | | | | Others | transcriptional regulator |
| Cre01.g001200.v5.5 | -0.006572241676701 | | | | GmPK6/AtMRK1 Family | PPC |
| Cre16.g664000.v5.5 | -0.00652833780901 | | | | SET | transcriptional regulator |
| Cre12.g529550.v5.5 | -0.006344181846351 | ALK1 | Aurora-like kinase | Aurora-Like Kinase; formerly known as CALK (Chlamydomonas Aurora-Like Kinase [gi:9716372; PMID: 11121779]. Serine/threonine protein kinase; regulates chromosome segregation and cytokinesis in mammals. Previously annotated as CALK1 | Unknown Function Kinase | PPC |
| Cre10.g451450.v5.5 | -0.006327485750599 | | | | GmPK6/AtMRK1 Family | PPC |
| Cre03.g146067.v5.5 | -0.006297562013251 | | | | Receptor Like Cytoplasmic Kinase VII | PPC |
| Cre12.g527000.v5.5 | -0.006291010292426 | | | | Calcium Dependent Protein Kinase | PPC |
| Cre01.g031300.v5.5 | -0.006276252802679 | | | | Calcium Dependent Protein Kinase | PPC |
| Cre02.g083750.v5.5 | -0.006264831531365 | | | | GARP->GARP-G2-like | transcription factor |
| Cre09.g395658.v5.5 | -0.006234290816226 | | | | CDC2 Like Kinase Family | PPC |
| Cre06.g278230.v5.5 | -0.006211933426252 | | | | SNF1 Related Protein Kinase (SnRK) | PPC |
| Cre02.g099550.v5.5 | -0.006141757577895 | | | | CTR1/EDR1 Kinase | PPC |
| Cre03.g181700.v5.5 | -0.006116544444264 | | | | SNF1 Related Protein Kinase (SnRK) | PPC |
| Cre01.g025050.v5.5 | -0.00607780258381 | | | | C2C2->C2C2-GATA | transcription factor |
| Cre13.g571200.v5.5 | -0.006046128199868 | | | | Others | transcriptional regulator |
| Cre19.g750597.v5.5 | -0.006025743752457 | | | | Calcium Dependent Protein Kinase | PPC |
| Cre09.g402478.v5.5 | -0.006015473173604 | | | | GmPK6/AtMRK1 Family | PPC |

**Table S17. The 100 regulatory genes (Protein Kinase (PPC), transcriptional regulators and transcription factors from the iTAK database) contributing the most to the high acetate state including annotation.**

**$G_2$ sorted**

| identifier | $G_2$ | geneName | defline | description | name | type |
|---|---|---|---|---|---|---|
| Cre04.g220700.v5.5 | 0.04369856428454 | ALK2 | Aurora-like kinase | Possible role in mitotic regulation. Note that the ALK1 model (originally called CALK) and several other kinase models in the genome are much weaker hits to the aurora family. | Unknown Function Kinase | PPC |
| Cre17.g735550.v5.5 | 0.038142448918727 | | | | MAP3K | PPC |
| Cre17.g742250.v5.5 | 0.037889614494085 | CDKG2 | Cyclin dependent kinase | Cyclin dependent kinase; Chlamydomonas specific subfamily; AASTLRE motif. Novel CDK subfamily. Also identified from nitrogen starvation experiments as nitrogen-controlled-gene NCG9 [PMID: 11469597] | CDC2 Like Kinase Family | PPC |
| Cre11.g467586.v5.5 | 0.036887593916565 | | | | Leucine Rich Repeat Kinase XI & XII | PPC |
| Cre12.g537400.v5.5 | 0.033665555387022 | | | | Unknown Function Kinase | PPC |
| Cre12.g522400.v5.5 | 0.032256067453098 | | | | MYB->MYB | transcription factor |
| Cre02.g075350.v5.5 | 0.03167216753854 | CNK1 | NimA-related protein kinase | NimA (never-in-mitosis) related kinase homolog 1 [gi:34334391, PMID: 15068267] | Unknown Function Kinase | PPC |
| Cre16.g666300.v5.5 | 0.031519331060252 | | | | CTR1/EDR1 Kinase | PPC |
| Cre08.g372550.v5.5 | 0.031133415301622 | CDKB1 | Plant specific cyclin dependent kinase | Plant specific cyclin dependent kinase; CDKB subfamily; PSTTLRE motif; mitotic regulator. Putative function in G2/M | CDC2 Like Kinase Family | PPC |
| Cre17.g705950.v5.5 | 0.031059150316697 | | | | GNAT | transcriptional regulator |
| Cre03.g187300.v5.5 | 0.030935825910406 | | | | Unknown Function Kinase | PPC |
| Cre13.g572600.v5.5 | 0.030570220382922 | | | | SET | transcriptional regulator |
| Cre12.g498650.v5.5 | 0.029003464255077 | ALK3 | Aurora-like kinase | Aurora-like kinase | Unknown Function Kinase | PPC |
| Cre03.g169100.v5.5 | 0.028742136016912 | | | | Calcium Dependent Protein Kinase | PPC |
| Cre03.g182700.v5.5 | 0.028052873091894 | | | | Others | transcriptional regulator |
| Cre11.g482700.v5.5 | 0.028038098173913 | | | | HMG | transcriptional regulator |
| Cre03.g199050.v5.5 | 0.02800482677449 | | | | IRE/NPH/PI dependent/S6 Kinase | PPC |
| Cre17.g745447.v5.5 | 0.02751613604963 | MAPK4 | Mitogen-Activated Protein Kinase | Hypothetical Mitogen-Activated Protein Kinase Homolog 4 | MAPK Family | PPC |
| Cre02.g087900.v5.5 | 0.027124369689123 | | | | GmPK6/AtMRK1 Family | PPC |
| Cre17.g713750.v5.5 | 0.027024657578258 | | Serine/threonine protein kinase | Serine/Threonine protein kinase. Previously annotated as STPK16 | Leucine Rich Repeat Kinase XI & XII | PPC |
| Cre12.g489000.v5.5 | 0.026820573804038 | | | | bZIP | transcription factor |
| Cre09.g404351.v5.5 | 0.025028037620979 | | | | SET | transcriptional regulator |
| Cre13.g564100.v5.5 | 0.024892825207606 | PTK2 | Putative tyrosine kinase | Putative tyrosine kinase identified through multi-level HMM library for kinase classification (PMID: 17557329) | GmPK6/AtMRK1 Family | PPC |
| Cre03.g144747.v5.5 | 0.024128903679894 | | | | MYB->MYB | transcription factor |
| Cre01.g040150.v5.5 | 0.023806999139333 | | | | WNK like kinase - with no lysine kinase | PPC |
| Cre03.g193900.v5.5 | 0.022721087924103 | | High mobility group protein, histone-like transcription factor | High mobility group protein, histone-like transcription factor. Has homology to DNA polymerase epsilon subunit | HMG | transcriptional regulator |
| Cre07.g355250.v5.5 | 0.022551713493427 | WEE1 | CDK inhibitory kinase | WEE1 kinase, CDK inhibitory kinase, dual specificity for serine/threonine and | Unknown Function Kinase | PPC |

| | | | | | | |
|---|---|---|---|---|---|---|
| | | | | tyrosine. Putative function in G2/M | | |
| Cre06.g249050.v5.5 | 0.022478731610186 | | | | MYB->MYB-related | transcription factor |
| Cre10.g464100.v5.5 | 0.022245176210763 | | | | MAP3K | PPC |
| Cre08.g361400.v5.5 | 0.021409446323135 | | | | CPP | transcription factor |
| Cre08.g384250.v5.5 | 0.020226886646373 | | | | SNF1 Related Protein Kinase (SnRK) | PPC |
| Cre09.g392300.v5.5 | 0.01986259129437 | | | | GNAT | transcriptional regulator |
| Cre11.g475100.v5.5 | 0.019194016407485 | | | | GNAT | transcriptional regulator |
| Cre11.g467585.v5.5 | 0.018165527752851 | | | | Unknown Function Kinase | PPC |
| Cre01.g023100.v5.5 | 0.018017488906327 | | | | AP2/ERF->AP2/ERF-ERF | transcription factor |
| Cre03.g197100.v5.5 | 0.017657577665787 | | | | MYB->MYB | transcription factor |
| Cre07.g351800.v5.5 | 0.016934310833512 | PTK12 | Protein tyrosine kinase | Putative protein tyrosine kinase identified through multi-level HMM library for kinase classification (PMID: 17557329) | Leucine Rich Repeat Kinase XI & XII | PPC |
| Cre09.g393728.v5.5 | 0.016375168970236 | | | | SET | transcriptional regulator |
| Cre10.g460700.v5.5 | 0.016083358935205 | | | | Phosphoenolpyruvate Carboxylase Kinase | PPC |
| Cre06.g261450.v5.5 | 0.015962678575517 | | | | HMG | transcriptional regulator |
| Cre16.g667450.v5.5 | 0.01558522033987 | TLP2 | Tubby-like protein | Tubby-like protein 2. Transcript upregulated during flagellar regeneration [PMID: 15738400]. | TUB | transcription factor |
| Cre06.g283200.v5.5 | 0.015324395523501 | | | | SET | transcriptional regulator |
| Cre17.g746247.v5.5 | 0.015232020701777 | | | | SET | transcriptional regulator |
| Cre02.g117813.v5.5 | 0.015203078587366 | | | | GmPK6/AtMRK1 Family | PPC |
| Cre16.g695600.v5.5 | 0.014654984163563 | DNJ10 | DnaJ-like protein and Myb-like transcription factor | DnaJ-like protein and Myb-like transcription factor; probably nuclear/cytosolic; similar to Chlamy Q6JX09_CHLRE GlsA-related protein and to Volvox GLSA; contains J-domain (pfam00226) at the N-terminus and Myb_DNA-binding-Domain (pfam00249) at the C-termin | MYB->MYB-related | transcription factor |
| Cre04.g224600.v5.5 | 0.014629159987299 | | | | bHLH | transcription factor |
| Cre14.g623926.v5.5 | 0.01446039477115 | | | | GmPK6/AtMRK1 Family | PPC |
| Cre05.g242600.v5.5 | 0.01415835584217 | | | | C2C2->C2C2-GATA | transcription factor |
| Cre11.g467526.v5.5 | 0.013719307845917 | | | | GNAT | transcriptional regulator |
| Cre16.g657150.v5.5 | 0.013555133173296 | | | | GNAT | transcriptional regulator |
| Cre12.g503450.v5.5 | 0.013421546206617 | | | | SET | transcriptional regulator |
| Cre09.g396065.v5.5 | 0.01341461110065 | ALK5 | Aurora-like kinase | Aurora-like kinase | Unknown Function Kinase | PPC |
| Cre04.g221900.v5.5 | 0.013150257227062 | | | | TRAF | transcriptional regulator |
| Cre17.g702650.v5.5 | 0.013073394790848 | | | | HMG | transcriptional regulator |
| Cre01.g004600.v5.5 | 0.0129389265082 | RWP12 | RWP-RK transcription factor | Putative RWP-RK domain transcription factor [PMID: 15785851]; model is very tentative | RWP-RK | transcription factor |
| Cre09.g408051.v5.5 | 0.012931101922438 | | | | mTERF | transcriptional regulator |
| Cre16.g694550.v5.5 | 0.012930777767894 | | | | CTR1/EDR1 Kinase | PPC |
| Cre06.g275450.v5.5 | 0.012562050108774 | | | | Leucine Rich Repeat Kinase XI & XII | PPC |
| Cre14.g614400.v5.5 | 0.012520823209283 | | | | SNF2 | transcriptional regulator |
| Cre01.g009550.v5.5 | 0.01251539694958 | | | | GNAT | transcriptional regulator |
| Cre13.g582650.v5.5 | 0.012426311340379 | LF4 | Mitogen-activated protein kinase, Long-flagella mutant LF4 protein | Mitogen-activated protein kinase 1 (gi:32130552); Long Flagella 4 (MOK homolog). Characterized by Berman et al. 2003 [PMID: 12842015]. Previously annotated as LF4 | CDC2 Like Kinase Family | PPC |
| Cre04.g231124.v5.5 | 0.012339484381277 | | | | C3H | transcription factor |

| | | | | | | |
|---|---|---|---|---|---|---|
| Cre09.g413050.v5.5 | 0.012157585620051 | | | | bZIP | transcription factor |
| Cre03.g174200.v5.5 | 0.012132002166181 | | | | TRAF | transcriptional regulator |
| Cre16.g668200.v5.5 | 0.011942779577514 | | | | PHD | transcriptional regulator |
| Cre12.g554051.v5.5 | 0.011837541293272 | | | | Leucine Rich Repeat Kinase XI & XII | PPC |
| Cre06.g262800.v5.5 | 0.011797399692414 | | | | PHD | transcriptional regulator |
| Cre06.g266150.v5.5 | 0.01176600192014 | | | | Unknown Function Kinase | PPC |
| Cre13.g575650.v5.5 | 0.011682514006965 | | | | CDC2 Like Kinase Family | PPC |
| Cre02.g078831.v5.5 | 0.011551034764689 | | | | CSD | transcription factor |
| Cre06.g267650.v5.5 | 0.011346126996628 | | | | IRE/NPH/PI dependent/S6 Kinase | PPC |
| Cre13.g574350.v5.5 | 0.011308614054252 | | | | GmPK6/AtMRK1 Family | PPC |
| Cre07.g341800.v5.5 | 0.011196114543812 | | | | NF-Y->NF-YB | transcription factor |
| Cre10.g424350.v5.5 | 0.011176784085381 | | | | Others | transcriptional regulator |
| Cre06.g293750.v5.5 | 0.011149197368497 | | | | C3H | transcription factor |
| Cre08.g385350.v5.5 | 0.010933438967516 | | | | AP2/ERF->AP2/ERF-ERF | transcription factor |
| Cre01.g054750.v5.5 | 0.010823019438999 | | | | SNF1 Related Protein Kinase (SnRK) | PPC |
| Cre07.g351850.v5.5 | 0.01081648362717 | | | | GNAT | transcriptional regulator |
| Cre03.g152500.v5.5 | 0.010692920533889 | | | | SET | transcriptional regulator |
| Cre12.g555800.v5.5 | 0.010502245058688 | | Serine/threonine protein kinase | Serine/Threonine protein kinase. Previously annotated as STPK2 | Phosphoenolpyruvate Carboxylase Kinase | PPC |
| Cre11.g481800.v5.5 | 0.010421386653772 | | | | CPP | transcription factor |
| Cre06.g278140.v5.5 | 0.010384656634527 | | | | Unknown Function Kinase | PPC |
| Cre01.g022950.v5.5 | 0.0103564795353 | | | | TRAF | transcriptional regulator |
| Cre02.g104450.v5.5 | 0.010355576639216 | | | | GmPK6/AtMRK1 Family | PPC |
| Cre04.g218050.v5.5 | 0.010344137514302 | RWP8 | RWP-RK transcription factor | Putative RWP-RK domain transcription factor [PMID: 15785851]; model is tentative | RWP-RK | transcription factor |
| Cre16.g674065.v5.5 | 0.010327271131356 | | | | Unknown Function Kinase | PPC |
| Cre06.g281000.v5.5 | 0.010218034899874 | | | | SET | transcriptional regulator |
| Cre08.g380452.v5.5 | 0.010046387101046 | | | | Unknown Function Kinase | PPC |
| Cre03.g149400.v5.5 | 0.010042867680205 | RWP11 | RWP-RK transcription factor | Putative RWP-RK domain transcription factor [PMID: 15785851]. Tandem pair with RWP4 with which there is amino acid homology around the RWP-RK domain and some intron conservation. Part of the model is tentative. | RWP-RK | transcription factor |
| Cre11.g481400.v5.5 | 0.010036797796712 | | | | Tousled like kinase | PPC |
| Cre12.g517000.v5.5 | 0.010030075179578 | MAPKKK7 | Mitogen-Activated Protein Kinase Kinase Kinase | MAP3K7 | S Domain Kinase (Type 2) | PPC |
| Cre09.g387023.v5.5 | 0.009929715119873 | | | | CTR1/EDR1 Kinase | PPC |
| Cre12.g503800.v5.5 | 0.009920099064303 | | | | SET | transcriptional regulator |
| Cre01.g052300.v5.5 | 0.00976963470837 | E2F1 | E2F family transcription factor | E2F family transcription factor. Putative function in G1/S and S/M. Positive regulator that controls commitment and S/M size checkpoints. Mutations at this locus suppress the small-cell phenotype of mutants in the MAT3 gene encoding a retinoblastoma-lik | E2F-DP | transcription factor |
| Cre03.g153050.v5.5 | 0.009688480817284 | RWP1 | RWP-RK transcription factor | Putative RWP-RK domain transcription factor [PMID: 15785851]; model is very tentative | RWP-RK | transcription factor |
| Cre06.g266100.v5.5 | 0.00968760449846 | | | | Unknown Function Kinase | PPC |
| Cre02.g084550.v5.5 | 0.009627351224876 | | | | GNAT | transcriptional regulator |
| Cre02.g116700.v5.5 | 0.009546499621585 | PTK11 | Protein tyrosine kinase | Putative protein tyrosine kinase identified | GmPK6/AtMRK1 Family | PPC |

| | | | | |
|---|---|---|---|---|
| | | through multi-level HMM library for kinase classification (PMID: 17557329) | | |
| Cre11.g467577.v5.5 | 0.00945790317502 | | MADS->MADS-M-type | transcription factor |
| Cre16.g683650.v5.5 | 0.009283807669016 | | CTR1/EDR1 Kinase | PPC |
